# Systematic Review to Inform a World Health Organization (WHO) Clinical Practice Guideline: Benefits and Harms of Structured and Standardized Education or Advice for Chronic Primary low back pain in Adults

**DOI:** 10.1007/s10926-023-10120-8

**Published:** 2023-11-22

**Authors:** Danielle Southerst, Cesar A. Hincapié, Hainan Yu, Leslie Verville, André Bussières, Douglas P. Gross, Paulo Pereira, Silvano Mior, Andrea C. Tricco, Christine Cedraschi, Ginny Brunton, Margareta Nordin, Jessica J. Wong, Gaelan Connell, Heather M. Shearer, Astrid DeSouza, Javier Muñoz Laguna, Joyce G. B. Lee, Daphne To, Rahim Lalji, Kent Stuber, Martha Funabashi, Léonie Hofstetter, Danny Myrtos, Andrew Romanelli, Brett Guist, James J. Young, Sophia da Silva-Oolup, Maja Stupar, Dan Wang, Kent Murnaghan, Carol Cancelliere

**Affiliations:** 1 Institute for Disability and Rehabilitation Research and Faculty of Health Sciences, Ontario Tech University, Oshawa, Canada; 2 EBPI-UWZH Musculoskeletal Epidemiology Research Group, University of Zurich and Balgrist University Hospital, Zurich, Switzerland; 3 Epidemiology, Biostatistics and Prevention Institute (EBPI), University of Zurich, Zurich, Switzerland; 4 University Spine Centre Zurich (UWZH), Balgrist University Hospital and University of Zurich, Zurich, Switzerland; 5 Département chiropratique, Université du Québec à Trois-Rivières, Trois-Rivières (Québec), Canada; 6 School of Physical and Occupational Therapy, Faculty of Medicine and Health Sciences, McGill University, Québec, Canada; 7 Department of Physical Therapy, University of Alberta, Edmonton, Canada; 8 Department of Neurosurgery, Centro Hospitalar Universitário São João, Porto, Portugal; 9 Faculty of Medicine, University of Porto, Porto, Portugal; 10 Department of Research and Innovation, Canadian Memorial Chiropractic College, Toronto, Canada; 11 Li Ka Shing Knowledge Institute, St. Michael’s Hospital, Unity Health Toronto, Toronto, Canada; 12 Epidemiology Division and Institute for Health Policy, Management, and Evaluation, Dalla Lana School of Public Health, University of Toronto, Toronto, Canada; 13 Queen’s Collaboration for Health Care Quality Joanna Briggs Institute Centre of Excellence, Queen’s University, Kingston, Canada; 14 Division of General Medical Rehabilitation, Geneva University and University Hospitals, Geneva, Switzerland; 15 Division of Clinical Pharmacology and Toxicology, Multidisciplinary Pain Centre, Geneva University Hospitals, Geneva, Switzerland; 16 EPPI-Centre, UCL Institute of Education, University College London, England, United Kingdom; 17 Department of Health Research Methods, Evidence and Impact, Faculty of Health Sciences, McMaster University, Hamilton, Canada; 18 Departments of Orthopedic Surgery and Environmental Medicine, NYU Grossman School of Medicine, New York University, New York, United States; 19 Bloorview Research Institute, Holland Bloorview Kids Rehabilitation Hospital, Toronto, Canada; 20 Department of Clinical Education, Canadian Memorial Chiropractic College, Toronto, Canada; 21 Parker University Research Center, Dallas, United States; 22 Department of Undergraduate Education, Canadian Memorial Chiropractic College, Toronto, Canada; 23 Center for Muscle and Joint Health, Department of Sports Science and Clinical Biomechanics, University of Southern Denmark, Odense, Denmark; 24 Schroeder Arthritis Institute, Krembil Research Institute, University Health Network, Toronto, Canada; 25 Department of Graduate Education, Canadian Memorial Chiropractic College, Toronto, Canada; 26 Library and Information Services, Canadian Memorial Chiropractic College, Toronto, Canada

**Keywords:** Low back pain, Systematic review, Meta-analysis, Education, Advice

## Abstract

**Purpose:**

Evaluate benefits and harms of education/advice for chronic primary low back pain (CPLBP) in adults to inform a World Health Organization (WHO) standard clinical guideline.

**Methods:**

Electronic databases were searched for randomized controlled trials (RCTs) assessing education/advice compared with placebo/sham, usual care, or no intervention (including comparison interventions where the attributable effect of education/advice could be isolated). We conducted meta-analyses and graded the certainty of evidence.

**Results:**

We screened 2514 citations and 86 full text RCTs and included 15 RCTs. Most outcomes were assessed 3 to 6 months post-intervention. Compared with no intervention, education/advice improved pain (10 RCTs, MD = -1.1, 95% CI -1.63 to -0.56), function (10 RCTs, SMD = -0.51, 95% CI -0.89 to -0.12), physical health-related quality of life (HRQoL) (2 RCTs, MD = 24.27, 95% CI 12.93 to 35.61), fear avoidance (5 RCTs, SMD = -1.4, 95% CI -2.51 to -0.29), depression (1 RCT; MD = 2.10, 95% CI 1.05 to 3.15), and self-efficacy (1 RCT; MD = 4.4, 95% CI 2.77 to 6.03). Education/advice conferred less benefit than sham Kinesio taping for improving fear avoidance regarding physical activity (1 RCT, MD = 5.41, 95% CI 0.28 to 10.54). Compared with usual care, education/advice improved pain (1 RCT, MD = -2.10, 95% CI -3.13 to -1.07) and function (1 RCT, MD = -7.80, 95% CI -14.28 to -1.32). There was little or no difference between education/advice and comparisons for other outcomes. For all outcomes, the certainty of evidence was very low.

**Conclusion:**

Education/advice in adults with CPLBP was associated with improvements in pain, function, HRQoL, and psychological outcomes, but with very low certainty.

**Supplementary Information:**

The online version contains supplementary material available at 10.1007/s10926-023-10120-8.

## Introduction

Guidelines for the management of low back pain (LBP) recommend education or advice (education/advice) as part of the first line of treatment [1]. Education/advice is defined as the provision of information delivered by a healthcare professional to improve a patient’s understanding of pain, guide management, or both [2]. It can be widely available globally where a suitably qualified workforce exists, or with access to online information and communication technologies. However, guidelines are inconsistent with respect to the content of the education/advice (e.g., reassurance of good prognosis, and advice on self-management) and modes of delivery (e.g., verbal or written, structured or unstructured).

Jones and colleagues recently published a systematic review (2021) [2] investigating the effect of education/advice compared with placebo or no education/advice in people with non-specific spinal pain (27 randomized controlled trials [RCTs]; 7,006 participants). The authors found that education/advice had a small effect on pain and disability in the short term (more than 2 weeks but less than or equal to 3 months) compared with placebo or no education/advice in people with non-specific spinal pain. However, little is known about the effects of education/advice over the long-term or effects versus other comparison interventions such as usual care. Furthermore, the benefits and harms specific to people with chronic primary LBP (CPLBP) – pain between the lower costal margin and the gluteal fold with no specific underlying cause of more than three months duration – remain unclear.

To develop clinical practice guideline recommendations for the management of CPLBP in adults, the World Health Organization (WHO) commissioned the current systematic review to update the evidence and expand the aims of the Jones review [2] by assessing additional comparators (i.e., usual care) and fundamental selected outcomes (i.e., health-related quality of life (HRQoL), psychological outcomes, social participation including work, health literacy, and change in medication use) that are often the specific target of education interventions. Furthermore, the WHO was interested in conducting pertinent subgroup analyses (e.g., gender/sex, race/ethnicity), disaggregating findings by educational content type and modes of delivery and focusing on people with CPLBP.

The objectives of this systematic review of RCTs were to determine: (1) the benefits and harms (as reported in RCTs) of education/advice compared with placebo/sham, usual care, or no intervention in the management of CPLBP in adults, including older adults (aged ≥ 60 years); and (2) whether the benefits and harms of education/advice vary by age, gender/sex, presence of leg pain, race/ethnicity, or national economic development of the countries where the RCTs were conducted.

## Methods

This systematic review was conducted as part of a series of reviews to inform the WHO guideline on the management of CPLBP in adults. Guideline development was ongoing at the time of submission of this manuscript. The methods are detailed in the methodology article of this series [3].

Briefly, we updated and expanded the scope of the previous high-quality systematic review by Jones et al. (2021) [2]. We registered our review protocol with Prospero (CRD42022314804) on 7 March 2022. We searched MEDLINE (Ovid), Cochrane Central Register of Controlled Trials (Wiley), Embase (Ovid), CINAHL (EBSCO), PEDRO, and the WHO International Clinical Trials Registry Platform (ICTRP) from the period of 1 September 2020 (end date of previous Cochrane review) to 9 March 2022 (see Online Resource 1). We also searched the reference lists of systematic reviews and included RCTs.

We included RCTs that compared education/advice to placebo/sham, usual care, or no intervention (including comparison interventions where the attributable effect of education/advice could be isolated e.g., education/advice + medication vs. same medication alone) in adults (aged ≥ 20 years) with CPLBP. Placebo or sham education/advice was operationalized as contact with a health professional but not the provision of information on LBP and its management (e.g., using a reflective and non-directive approach) [2]. Detuned ultrasound, as well as other sham interventions, could be included. We considered education/advice to include any education, advice, or information given by a healthcare practitioner to improve a patient’s understanding of pain or its appropriate management [2]. This included education on being physically active and how to self-manage LBP, reassurance about the positive prognosis and self-limiting nature of LBP, and pain management education, including pain neuroscience (‘explain pain’) education interventions [2, 4]. Education/advice could be structured (e.g., following a specific book, presentation) or unstructured (e.g., general improvised advice on exercises and lifestyle modifications to manage back pain without following a specific education program). It could be delivered in any care setting (e.g., primary healthcare, workplace); using any modality (verbal, written, electronic, or a combination of these); over single or multiple sessions; to groups or individuals; and by any health practitioner [2]. We excluded RCTs of interventions providing public education, such as mass media campaigns, social marketing, or other public-facing education including websites that are not provided in the context of a clinical encounter. Further details on the eligibility criteria can be found in the methodology article in this series [3].

In addition to the main critical outcomes requested by the WHO Guideline Development Group (GDG) and assessed for all reviews in this series (pain, function, HRQoL, harms, psychological functioning, social participation including work), we also assessed additional critical outcomes requested by the WHO GDG for this review - the change in use of medications (all adults and older adults aged ≥ 60 years), health literacy (all adults), and falls (older adults only). We reported outcomes based on post-intervention follow-up intervals including: (1) immediate term (closest to 2 weeks after the intervention period); (2) short term (closest to 3 months after the intervention period); (3) intermediate term (closest to 6 months after the intervention period); (4) long term (closest to 12 months after the intervention period); and (5) extra-long term (more than 12 months after the intervention period).

We assessed between-group differences to determine the magnitude of the effect of an intervention and to assess its effectiveness [5, 6] (details in the methodology article in this series) [3]. Briefly, we considered a mean difference (MD) of ≥ 10% of the scale range or ≥ 10% difference in risk for dichotomous outcomes to be a minimally important difference (MID) [7, 8]. If the standardized mean difference (SMD) was calculated, SMD ≥ 0.2 was considered a MID [9].

Pairs of reviewers independently screened studies for eligibility, and critically appraised risk of bias (ROB) using the Cochrane ROB 1 tool [10], modified from the Cochrane Back and Neck Methods Guidelines [11]. One reviewer extracted data for all included RCTs, which was then verified by a second reviewer. Any disagreements were resolved by consensus between paired reviewers or with a third reviewer when necessary. Forms and guidance for screening, ROB assessment, and data extraction were adapted from those used by Hayden et al. in the conduct of the ‘exercise for chronic low back pain’ collaborative review, in which members of our team participated [12]. The forms were modified and completed using a web-based electronic systematic review software DistillerSR Inc. [13].

In addition to the main sub-group analyses conducted for all reviews in this series of papers (age, gender/sex, presence of leg pain, race/ethnicity, national economic development of country where RCT was conducted), we planned to conduct the following pre-specified sub-group and sensitivity analyses: education/advice content type (i.e., mixed content or pain neuroscience), delivery mode (i.e., verbal, written, or electronic), and removal of RCTs rated as high ROB.

We conducted random-effects meta-analyses and narrative synthesis where meta-analysis was not appropriate [14], and graded the certainty of evidence using Grading of Recommendations Assessment, Development and Evaluation (GRADE) [15]. Comparisons to no intervention and sets of interventions where the specific attributable effect of education/advice could be isolated (e.g., education/advice + treatment B versus treatment B alone) were combined in meta-analyses. Meta-analyses were conducted using R statistical packages [16, 17]. GRADE Evidence Profiles and GRADE Summary of Findings tables were developed using GRADEpro software [18].

## Results

**Fig. 1:**
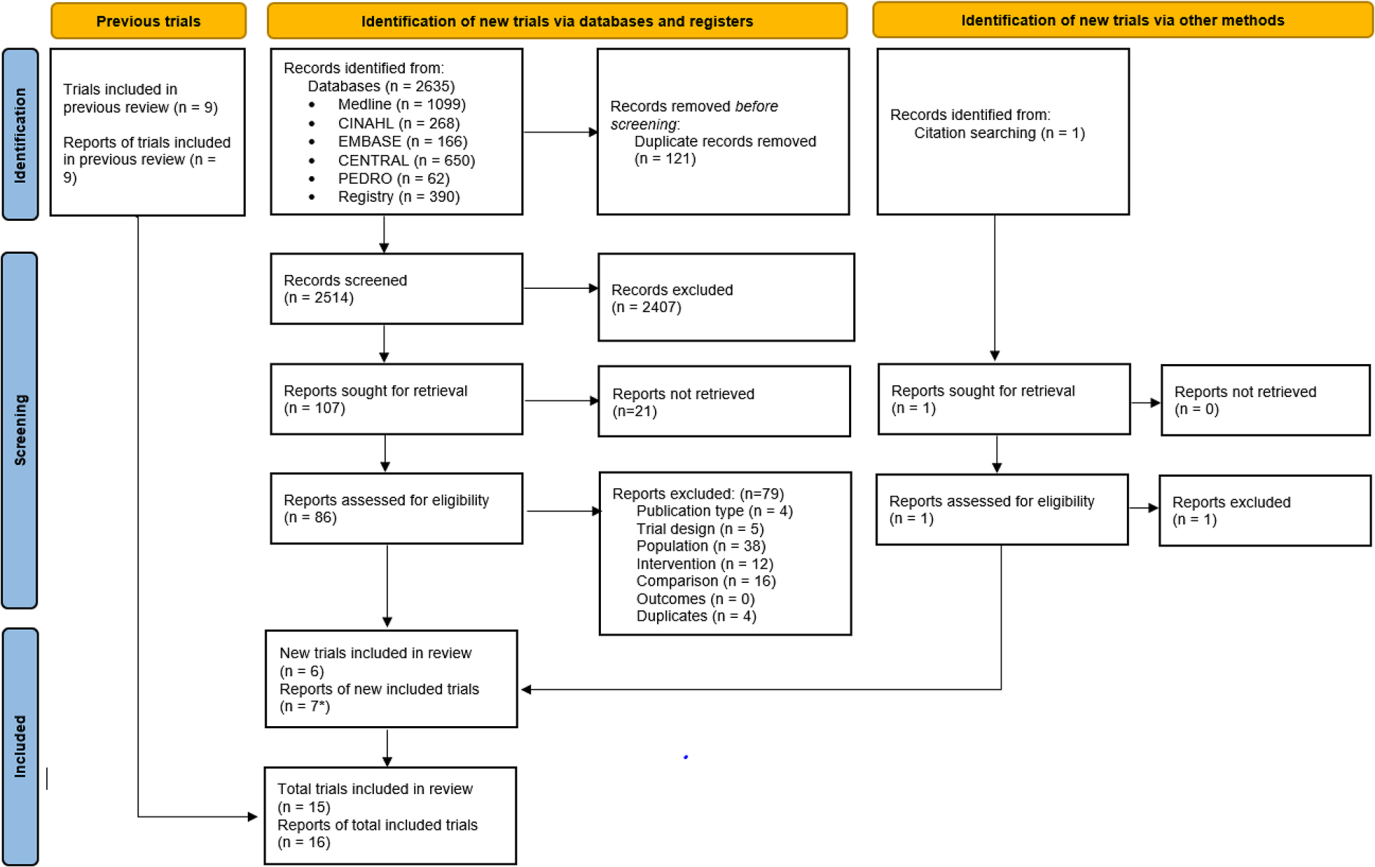
Flow diagram of literature search *1 trial was reported in 2 reports

We screened 2514 records and 86 full-text reports (Fig. 1). We identified 21 unpublished RCTs in the WHO ICTRP, of which we contacted the authors with contact information listed (8 of the 21). One of eight authors responded and informed us that the RCT was ongoing. Thus, none of the 21 unpublished RCTs identified in the WHO ICTRP were included. We included 15 published RCTs (16 reports) [19–34] of mainly structured and standardized education/advice with a total of 1403 adults (ranging from 12 to 250 adults per RCT) from health care and occupational settings (see Online Resources 2, 3). The RCTs were conducted in high-income economies [35]: Finland (1 RCT) [29], Italy (1 RCT) [27], Korea (1 RCT) [25], Portugal (1 RCT) [28], and Spain (2 RCTs) [21, 33]; upper-middle income economies: Brazil (3 RCTs) [22, 24, 26], China (1 RCT) [34], and Turkey (2 RCTs) [19, 30]; and lower-middle income economies: Iran (2 RCTs, 3 reports) [23, 31, 32] and Nigeria (1 RCT) [20]. The mean age of participants ranged from 25 to 73 years; two RCTs assessed adults ≥ 60 years (n = 60) [22, 25]. The percentage of females within the RCTs ranged from 0 to 100%. In two RCTs, adults had CPLBP without leg pain [21, 33], in five RCTs (six reports) adults had CPLBP either with or without: non-radicular leg pain (3 RCTs) [25, 27, 28], radicular leg pain (1 RCT) [19], or leg pain not otherwise specified (1 RCT, 2 reports) [31, 32]. The presence of leg pain was not reported in eight RCTs [20, 22–24, 26, 29, 30, 34]. Where reported by authors, CPLBP duration ranged from 11 months to 14 years.

The education/advice interventions predominantly involved mixed content (i.e., two or more content types such as ergonomic advice, self-management advice, etc.), or pain neuroscience education (‘explain pain’) delivered in verbal or combined verbal and written methods. Approximately half of the RCTs reported delivering their education/advice intervention in group format versus individually. The number of sessions delivered ranged from 1 to 16, with the duration of each session ranging from 10 to 120 min. Education was compared to sham (sham Kinesio taping) (1 RCT) [24]; usual care (2 RCTs) [19, 27]; no intervention (6 RCTs in 7 reports) [20, 22, 23, 26, 29, 31, 32] or comparison interventions where the attributable effect of education/advice could be isolated (6 RCTs) [21, 25, 28, 30, 33, 34]. Most of the RCTs assessed pain and function in the short term (closest to 3 months) (Table 1).

The RCTs were rated as overall high (13, 87%), or unclear (2, 13%) ROB (see Online Resource 4). The agreement on overall ROB ratings was high (weighted overall kappa score 0.96).

**Table 1:** Number of included RCTs by comparison and outcome

Outcomes assessed	Follow-up				
	Immediate (2 weeks)	Short (3 months)	Intermediate (6 months)	Long (12 months)	Extra-long (> 12 months)
**Education/advice versus no intervention** ^**a**^					
Pain	-	10 (older adults: 2)	1	1	-
Function	-	10 (older adults: 2)	1	1	1
HRQoL	-	2	-	-	-
Fear avoidance	-	5 (older adults: 1)	-	-	1
Catastrophizing	-	2	-	-	-
Depression	1	-	1	-	-
Anxiety	-	-	-	-	-
Self-efficacy	1	-	1	-	-
Social participation	-	-	-	-	1
Medication use	-	-	-	-	-
Health literacy	-	-	-	-	-
Harms	-	-	-	-	1
**Education/advice versus sham**					
Pain	-	1	-	-	-
Function	-	1	-	-	-
HRQoL	-	-	-	-	-
Fear avoidance	-	1	-	-	-
Catastrophizing	-	-	-	-	-
Depression	-	-	-	-	-
Anxiety	-	-	-	-	-
Self-efficacy	-	-	-	-	-
Social participation	-	-	-	-	-
Medication use	-	-	-	-	-
Health literacy	-	-	-	-	-
Harms	-	-	-	-	-
**Education/advice versus usual care**					
Pain	-	2	1	-	-
Function	-	1	1	-	-
HRQoL	-	1	1	-	-
Fear avoidance	-	-	-	-	-
Catastrophizing	-	-	-	-	-
Depression	-	-	-	-	-
Anxiety	-	-	-	-	-
Self-efficacy	-	-	-	-	-
Social participation	-	-	-	-	-
Medication use	-	-	-	-	-
Health literacy	-	-	-	-	-
Harms	-	-	-	-	-

HRQoL: health-related quality of life. Number of RCTs including older adults (aged ≥ 60 years) are indicated in brackets.

^a^Includes comparison interventions where the attributable effect of education/advice could be isolated

### Certainty of Evidence

The certainty of the evidence for all outcomes was very low and was downgraded due to ROB, inconsistency, indirectness, and imprecision of the effect estimates (see Online Resources 5, 6 and 7).

### Education/Advice Versus no Intervention (Including Comparison Interventions Where the Attributable Effect of Education/Advice could be Isolated)

#### All Adults

**Fig. 2:**
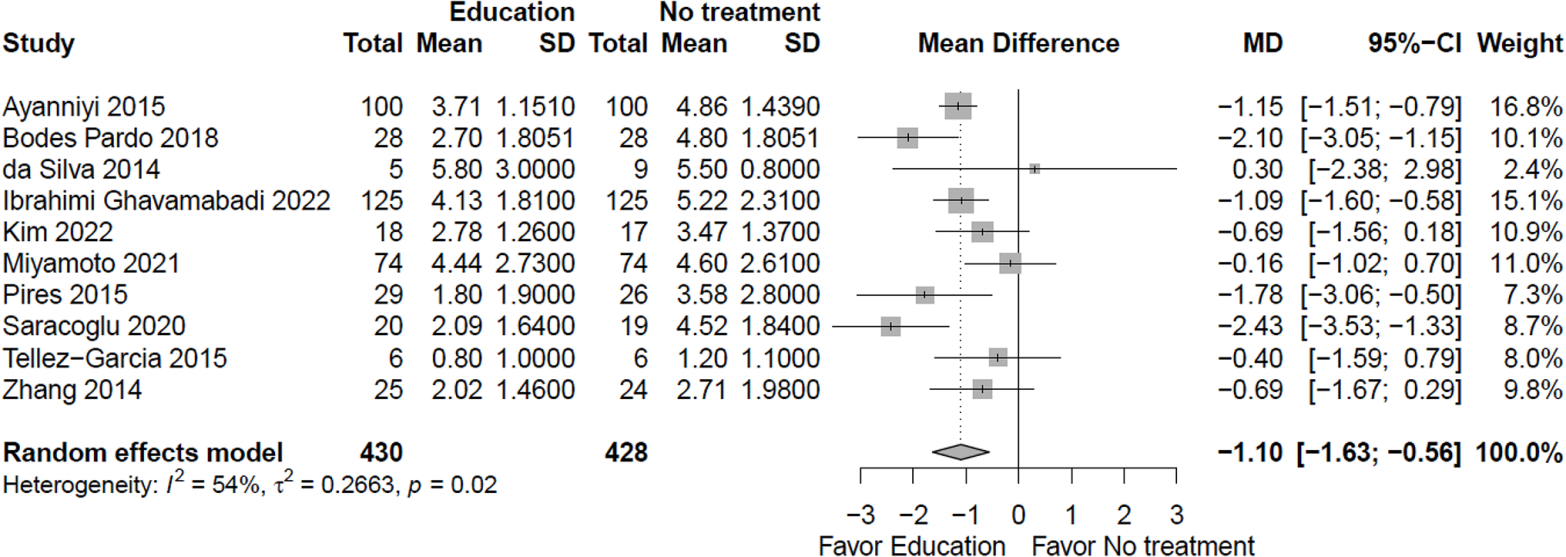
Education versus no intervention, and comparison interventions where the attributable effect of education/advice could be isolated for pain in the short term (closest to 3 months); scale range is 0 to 10

Due to very low certainty evidence, it is uncertain whether education/advice decreases ***pain*** *(scale 0 to 10, 0 = no pain)* in the short term (10 RCTs; mean difference (MD) = -1.1, 95% CI -1.63 to -0.56) (see Fig. 2, Online Resource 7, plot 1.1.1) [20–23, 25, 26, 28, 30, 33, 34] or long term (1 RCT; MD = -1.35, 95% CI -2.34 to -0.36) (plot 1.1.3) [26]. It is uncertain whether education/advice makes little or no difference to pain in the intermediate term (1 RCT; MD = -0.55, 95% CI -1.49 to 0.39) (plot 1.1.2) [26], or extra-long term (1 RCT; MD = -8.00, 95% CI -18.14 to 2.14) (plot 1.1.4) [29].

**Fig. 3:**
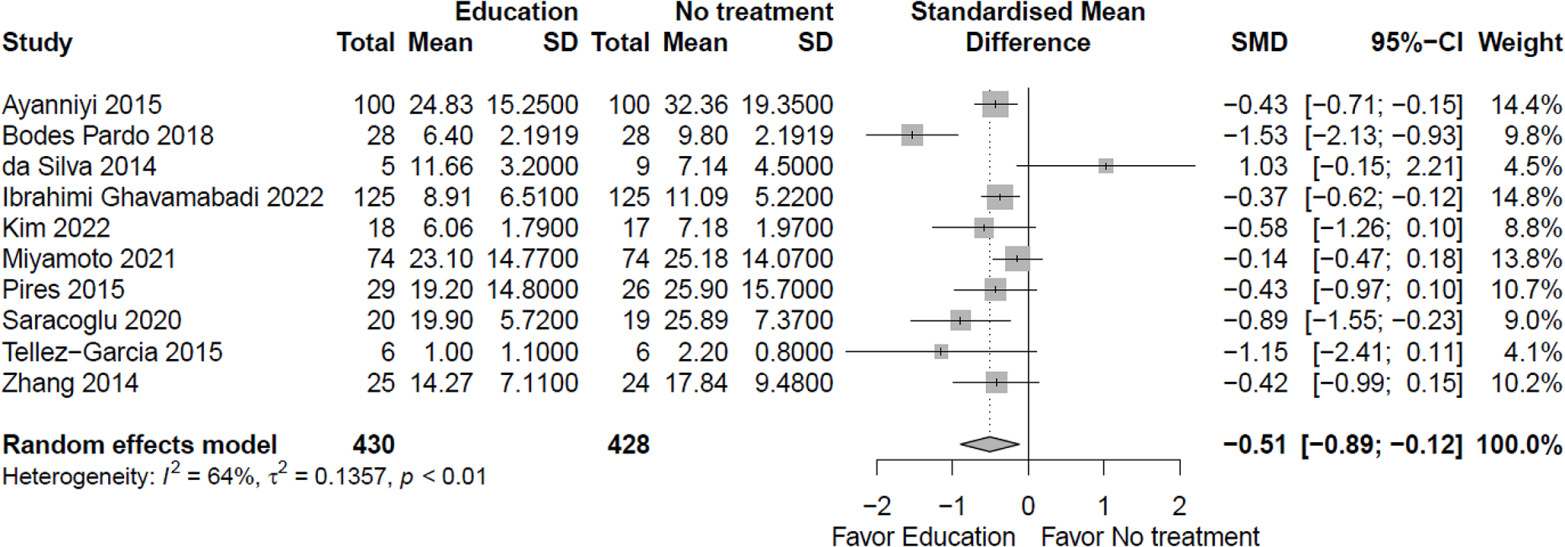
Education versus no intervention, and comparison interventions where the attributable effect of education/advice effect could be isolated for function in the short term (closest to 3 months)

Due to very low certainty evidence, it is uncertain whether education/advice decreases ***functional limitations*** in the short term (10 RCTs; standardized mean difference (SMD) = -0.51, 95% CI -0.89 to -0.12; benefit indicated by lower values) (Fig. 3, Online Resource 7, plot 1.2.1) [20–23, 25, 26, 28, 30, 33, 34]. It is uncertain whether education/advice makes little or no difference to function in the intermediate term (1 RCT; MD = -2.86, 95% CI -7.51 to 1.79; scale 0 to 100, 0 = no functional limitation) (plot 1.2.2) [26], long term (1 RCT; MD = -4.66, 95% CI -9.68 to 0.36; scale 0 to 100, 0 = no function limitation) (plot 1.2.3) [26], or extra-long term (1 RCT; MD = -1.5, 95% CI -3.42 to 0.42; scale 0 to 24, 0 = no functional limitation) (plot 1.2.4) [29].

Due to very low certainty evidence, in the short term, it is uncertain whether education/advice improves the *physical component summary (PCS)* of ***HRQoL ****(scale 0 to 100, 0 = poor quality of life)* (2 RCTs; MD = 24.27, 95% CI 12.93 to 35.61) (plot 1.3.1) [23, 34]. In addition, it is uncertain whether education/advice makes little or no difference to the *mental component summary (MCS)* of HRQoL (2 RCTs; MD = 13.99, 95% CI -62.04 to 90.03) (plot 1.4.1) [23, 34].

**Fig. 4:**
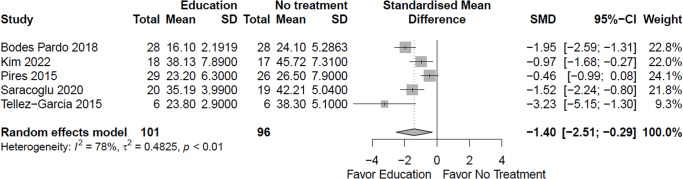
Education versus no intervention, and comparison interventions where the attributable effect of education/advice could be isolated for fear avoidance in the short term (closest to 3 months)

Due to very low certainty evidence, it is uncertain whether education/advice reduces ***fear avoidance ****(benefit indicated by lower values)* in the short term (5 RCTs; SMD = -1.4, 95% CI -2.51 to -0.29) (see Fig. 4; Online Resource 7, plot 1.6.1) [21, 25, 28, 30, 33]. It is uncertain whether education/advice makes little or no difference to fear avoidance in the extra-long term (1 RCT; MD = -1.0, 95% CI -7.13 to 5.13; scale 13 to 78) (plot 1.6.2) [21].

Due to very low certainty evidence, it is uncertain whether education/advice makes little or no difference to ***catastrophizing****(scale 0 to 52, 0 = no catastrophizing)* in the short term (2 RCTs; MD = -10.19, 95% CI -55.46 to 35.07) (plot 1.7.1) [21, 25].

Due to very low certainty evidence, it is uncertain whether education/advice improves ***depression ****(scale 4 to 20, benefit indicated by higher values)* in the immediate (1 RCT; MD = 2.10, 95% CI 1.05 to 3.15) (plot 1.8.1) or intermediate term (1 RCT; MD = 1.5, 95% CI 0.5 to 2.5) (plot 1.8.2) [32]. In the intermediate term, the effect estimate did not reach the threshold for a minimally important between-group difference (MD = 1.6).

Due to very low certainty evidence, it is uncertain whether education/advice improves ***self-efficacy ****(scale 7 to 35, benefit indicated by higher values)* in the immediate (1 RCT; MD = 4.4, 95% CI 2.77 to 6.03) (plot 1.9.1) [32], or intermediate term (1 RCT; MD = 1.60, 95% CI 0.04 to 3.16) (plot 1.9.2) [31]. In the intermediate term the effect estimate did not reach the threshold for a minimally important between-group difference (MD = 2.8).

Due to very low certainty evidence, it is uncertain whether education/advice makes little or no difference to ***social participation ****(number of sickness days, benefit indicated by lower values)* in the extra-long term (1 RCT; MD = 11.0, 95% CI -22 to 44) (plot 1.10.1) [29].

Due to very low certainty evidence, it is uncertain whether education/advice makes little or no difference to ***adverse events*** in the extra-long term (1 RCT) (no plot, narrative synthesis) [29]. No adverse events were reported among adults with CPLBP receiving either education/advice or no intervention. None of the other RCTs assessed adverse events.

#### Older Adults (aged ≥ 60 Years)

Due to very low certainty evidence, in the short term, it is uncertain whether education/advice makes little or no difference to ***pain ****(scale 0 to 10, 0 = no pain)* (2 RCTs; MD = -0.5, 95% CI -5.42 to 4.41) (plot 1.11.1) [22, 25]; or ***function ****(benefit indicated by lower values)* (2 RCTs; SMD = -0.02, 95% CI -9.79 to 9.76) (plot 1.12.1) [22, 25], or whether it reduces ***fear avoidance ****(benefit indicated by lower values)* in older female adults (1 RCT; SMD = -0.97, 95% CI -1.68 to -0.27) (plot 1.13.1) [25].

### Education/Advice Versus Sham

One RCT compared education/advice to sham (sham Kinesio taping) in the short term [24]. Based on very low certainty evidence, it is uncertain whether sham Kinesio taping is favoured over education/advice for reducing ***pain ****(scale 0 to 10, 0 = no pain)* in the short term (1 RCT; MD = 0.22, 95% CI 0.05 to 0.39) (plot 2.1.1) [24]. However, the effect estimate did not reach the threshold for a minimally important between-group difference (MD = 1).

Due to very low certainty evidence, it is uncertain whether education/advice makes little or no difference to ***functional limitations ****(scale 0 to 50, 0 = no functional limitations)* in the short term (1 RCT; MD = 0.2, 95% CI -5.7 to 6.1) (plot 2.2.1) [24].

Due to very low certainty evidence, it is uncertain whether sham Kinesio taping is favoured over education/advice for reducing ***fear avoidance (physical activity)****(scale 0 to 24, 0 = no fear avoidance)* (1 RCT; MD = 5.41, 95% CI 0.28 to 10.54) (plot 2.3.1), or whether education/advice makes little or no difference to ***fear avoidance (work)****(scale 0 to 42, 0 = no fear avoidance)* (1 RCT; MD = 2.64, 95% CI -0.54 to 5.82) (plot 2.4.1) [24].

### Education/Advice Versus Usual Care

Due to very low certainty evidence, it is uncertain whether education/advice makes little or no difference to ***pain ****(scale 0 to 10, 0 = no pain)* in the short term (2 RCTs; MD = -2.49, 95% CI -10.73 to 5.75) (plot 3.1.1) [19, 27], or whether education/advice decreases pain in the intermediate term (1 RCT; MD = -2.10, 95% CI -3.13 to -1.07) (plot 3.1.2) [27].

Due to very low certainty evidence, it is uncertain whether education/advice reduces ***functional limitations ****(scale 0 to 50, 0 = no functional limitations)* in the short (1 RCT; MD = -7.80, 95% CI -14.28 to -1.32) (plot 3.2.1) or intermediate term (1 RCT; MD = -9.2, 95% CI -16.5 to -1.9) (plot 3.2.2) [27].

Due to very low certainty evidence, it is uncertain whether education/advice makes little or no difference to the ***PCS*** of ***HRQoL ****(scale 0 to 100, 0 = poor quality of life)* in the short (1 RCT; MD = 2.50, 95% CI -1.41 to 6.41) (plot 3.3.1) or intermediate term ((1 RCT; MD = 2.40, 95% CI -1.56 to 6.36) (plot 3.3.2) [27]. It is uncertain whether education/advice improves the ***MCS ****(scale 0 to 100, 0 = poor quality of life)* in the short (1 RCT; MD = 9.40, 95% CI 2.7 to 16.1) (plot 3.4.1) or intermediate term (1 RCT; MD = 7.20, 95% CI 0.53 to 13.87) (plot 3.4.2) [27]. However, these effect estimates did not reach the threshold for what we considered to be a minimally important between-group difference (MD = 10).

### Results of Subgroup and Sensitivity Analyses

The results of the subgroup analyses did not substantially alter our main findings. For all comparisons, the subgroups were small (consisting of 1–2 RCTs with sample sizes ranging from 5 to 125 participants per group) and yielded small, pooled effects with marked imprecision (wide 95% CIs) and unclear clinical effects (see Online Resource 7). We did not conduct a sensitivity analysis removing the RCTs judged to have high ROB since most RCTs were judged as overall high ROB (14, 88%).

## Discussion

The evidence regarding the benefits and harms of education/advice for CPLBP in adults is based on 15 RCTs (16 reports) (n = 1403 total adults, n = 60 older adults). Most RCTs were rated as having a high overall ROB and the certainty of the evidence for all outcomes was very low. Compared to no intervention (including comparison interventions where the attributable effect of education/advice could be isolated), evidence suggested that education/advice is associated with a reduction in depression and improvement in self-efficacy immediately post-intervention. In the short term, education/advice is associated with greater improvements in pain, function, HRQoL (physical), and fear avoidance (including in older female adults). In the long-term, education/advice is associated with greater reductions in pain. However, education/advice was associated with little or no added benefit for other outcomes including catastrophizing, social participation, and adverse events. Compared to sham Kinesio taping in the short-term, education/advice conferred less benefits for pain and fear avoidance related to physical activity, and little to no benefit for function and fear avoidance related to work. Compared to usual care, evidence suggested that education/advice is associated with short-term improvements in function and intermediate-term improvements in pain and function. Education/advice was associated with little to no added benefit for HRQoL.

Our review adds to the findings reported by Jones et al. [2] through the inclusion of seven additional RCTs [23–27, 31, 32]. Our findings align with Jones et al. in that education/advice had a small effect on pain and disability in the short term. Our review adds findings related to the impact of education/advice on psychological outcomes.

Other related systematic reviews have focused on pain neuroscience education [36–40], an education intervention which aims to increase a patient’s knowledge of pain, the nervous system and factors that modulate pain [37], and patient education materials alone [41]. Overall, the majority of these agree with our findings. Pain neuroscience education has been found to be associated with small improvements in low-back related pain and disability [37, 39], and psychological and behavioural outcomes [36–39]; and patient education materials improved pain intensity, quality of life, global improvement, self-efficacy, fear avoidance and long-term stress [41].

Our systematic review has several strengths. First, the review team included international clinical and methodological experts with experience in the fields of LBP, systematic reviews, and evidence syntheses, and answering important policy questions from the WHO. Second, our reviews included comprehensive and peer-reviewed literature search strategies without language restrictions. Third, for screening and ROB assessments, a member of the core team (most trained and reliable in screening and ROB judgements) formed at least half of the screening and ROB assessment pairs. Fourth, for the ROB assessments, we did not rely on the number of items at ROB or summary scores, as was done in other systematic reviews [2]. Rather, we developed and used adjunct guidance forms based on the ROB 1 criteria [10, 11], allowing reviewers to consider important critical flaws [3]. Using these forms, the agreement on overall ROB judgements was high. Fifth, we maintained transparency in all steps of the review, such as providing detailed ROB assessments and footnotes for grading the certainty of the evidence (see Online Resources 4, 5). Providing comprehensive notes regarding our assessments allows readers to better understand how we came to our judgments, allowing them to come to their own judgements and conclusions.

Our review has potential limitations worth mentioning. First, we may have missed identifying potentially relevant RCTs. However, we tried to mitigate this through comprehensive, peer-reviewed literature search strategies developed with experienced health sciences librarians and searching the reference lists of included RCTs and related systematic reviews. Second, we did not search the grey literature (the WHO commissioned systematic reviews of RCTs published in the peer-reviewed literature). Excluding the grey literature has the potential of introducing publication bias in a review as trials found in the published literature tend to show larger effects of interventions compared to those found in the grey literature [42]. That said, we do not have strong evidence to suggest our review was impacted by publication bias, based on our publication bias assessment. Furthermore, we searched for unpublished RCTs in the WHO ICTRP registry and contacted authors of unpublished RCTs. However, only one author responded, and the reason given for non-publication was an ongoing RCT. While we are uncertain how publication bias may have impacted our findings, unpublished studies have been shown to represent a small proportion of studies and rarely impact the results and conclusions [43]. The inclusion of these types of studies could be especially important in scenarios with a limited number of applicable studies or when there are dubious personal interests involved in the published research [43]. Finally, the method of combining different sets of comparison interventions (i.e., no intervention and sets of interventions where the attributable effect of education/advice could be isolated) in meta-analysis may have contributed to the heterogeneity of the studies.

We identified some gaps in the evidence. First, for all adults, there were no RCTs assessing outcomes of social participation (including work), medication use or health literacy at any time point. For older adults specifically, no RCTs assessed health-related quality of life, adverse events/harms, depression, catastrophizing, anxiety, self-efficacy, change in use of medications or falls. None of the RCTs assessed whether benefits or harms varied by race/ethnicity. Furthermore, there were too few RCTs/participants to adequately assess the impact of other variables including sociodemographic (e.g., gender), clinical (e.g., presence of leg pain), and treatment-related (e.g., content, structure, setting, dose). Future RCTs should consider assessing psychological outcomes and health literacy as these outcomes are the target of most education interventions.

## Conclusion

Based on very low certainty evidence, education/advice in adults with CPLBP was associated with improvements in pain, function, HRQoL and psychological outcomes compared to no intervention (including interventions where the attributable effect of education/advice could be isolated), or usual care. However, education/advice conferred less benefit than sham Kinesio taping for improving fear avoidance related to physical activity, and no substantial differences were observed for the other outcomes. Although findings of this review are likely to change with further studies, it is important to place results within the broader context of clinical practice. Education is consistently recommended as part of an evidence-based, ethical, and patient-centred approach to the management of musculoskeletal conditions. Therefore, despite very low certainty evidence, continuing to offer education as part of a package of evidence-based interventions for the management of CPLBP seems appropriate.

### Electronic Supplementary Material

Below is the link to the electronic supplementary material.

Supplementary Material 1

## Data Availability

The datasets generated during and/or analysed during the current study are available from the corresponding author on reasonable request.
