# Supplementary material for: Systematic Review to Inform a World Health Organization (WHO) Clinical Practice Guideline: Benefits and Harms of Structured and Standardized Education or Advice for Chronic Primary low back pain in Adults
Source: J Occup Rehabil. 2023 Nov 22;33(4):625–35. doi: 10.1007/s10926-023-10120-8 (PMC10684630; doi:10.1007/s10926-023-10120-8)
Supplement: Supplementary file 1 — Supplementary Material 1 [file 10926_2023_10120_MOESM1_ESM.docx]

Systematic review to inform a World Health Organization (WHO) clinical practice guideline: Benefits and harms of structured and standardized education or advice for chronic primary low back pain in adults: Supplementary Information

Southerst D, Hincapié CA, Yu H, Verville L, Bussières A, Gross DP, Pereira P, Mior S, Tricco AC, Cedraschi C, Brunton G, Nordin M, Wong JJ, Connell G, Shearer HM, DeSouza A, Muñoz Laguna J, Lee J, To D, Lalji R, Stuber K, Funabashi M, Hofstetter L, Myrtos D, Romanelli A, Guist BP, Young J, da Silva-Oolup S, Stupar M, Wang D, Murnaghan K, Cancelliere C

Corresponding authors:

Carol Cancelliere

Institute of Disability and Rehabilitation Research and Faculty of Health Sciences, Ontario Tech University, Oshawa, Ontario, Canada

Email: [carolina.cancelliere@ontariotechu.ca](mailto:carolina.cancelliere@ontariotechu.ca)

Cesar A. Hincapié

EBPI-UWZH Musculoskeletal Epidemiology Research Group, University of Zurich and Balgrist University Hospital, Zurich, Switzerland

Epidemiology, Biostatistics and Prevention Institute (EBPI), University of Zurich, Zurich, Switzerland

University Spine Centre Zurich (UWZH), Balgrist University Hospital and University of Zurich, Zurich, Switzerland

Email: [cesar.hincapie@uzh.ch](mailto:cesar.hincapie@uzh.ch)

**Online Resource 1.** Literature search strategies

**A. Database & Platform:** MEDLINE (Ovid)

**Years of search:** September 1, 2020– March 9, 2022

**Date search run:** March 9, 2022

**Number of records retrieved:** 1099

**Search Strategy:**

1. Low Back Pain/
2. exp Back Pain/
3. Back Injuries/
4. exp Back Muscles/in [Injuries]
5. Intervertebral Disc Degeneration/
6. Intervertebral Disc Displacement/
7. Intervertebral Disc/in [Injuries]
8. Lumbar Vertebrae/in [Injuries]
9. Lumbosacral Plexus/in [Injuries]
10. Lumbosacral Region/in [Injuries]
11. Coccyx/in [Injuries]
12. Osteoarthritis, Spine/
13. Osteoarthritis/
14. Piriformis Muscle Syndrome/
15. Polyradiculopathy/
16. Sacroiliac Joint/in [Injuries]
17. Sciatica/
18. Spinal Curvatures/
19. Spinal Diseases/
20. Spinal Injuries/
21. Spinal Stenosis/
22. exp Spondylolysis/
23. Spondylosis/
24. Synovial Cyst/
25. Zygaphophyseal Joint/in [Injuries]
26. ((low* adj2 (back adj2 pain*)) or (low-back* adj2 pain*) or (lower-back* adj2 pain*) or (low* adj2 back-pain*)).mp.
27. ((low* adj2 (back adj2 injur*)) or (low-back* adj2 injur*) or (lower-back* adj2 injur*) or (low* adj2 back-injur*)).mp.
28. ((low* adj2 (back adj2 trauma*)) or (low-back adj2 trauma*) or (lower-back* adj2 trauma*) or (low* adj2 back-trauma*)).mp.
29. ((low* adj2 (trunk adj2 pain*)) or (lower-trunk* adj2 pain*) or (low* adj2 trunk-pain*)).mp.
30. lumbar* adj3 (disc* adj3 (extru* or degenerat* or displac* or herniat* or prolaps* or sequestered or slipped or protru* or avuls*)).mp.
31. lumbar* adj3 (disk* adj3 (extru* or degenerat* or displac* or herniat* or prolaps* or sequestered or slipped or protru* or avuls*)).mp.
32. lumbar* adj3 (pain* or facet* or (nerve adj2 root*) or osteoarth* or radicul* or stenos* or spondylo* or zygapophys* or injur* or discomfort* or dysfunction* or sore* or herniat*).mp.
33. lumbo* adj3 (pain* or facet* or (nerve adj2 root*) or osteoarth* or radicul* or stenos* or spondylo* or zygapophys* or injur* or discomfort* or dysfunction* or sore* or herniat*).mp.
34. back adj3 (ach* or injur* or pain* or sprain* or strain* or disorder*).mp.
35. backach*.mp.
36. back-pain*.mp.
37. intervertebral* adj3 (disc* adj3 (extru* or degenerat* or displac* or herniat* or prolaps* or sequestered or slipped or protru* or avuls*)).mp.
38. intervertebral* adj3 (disk* adj3 (extru* or degenerat* or displac* or herniat* or prolaps* or sequestered or slipped or protru* or avuls*)).mp.
39. coccy* adj2 (ach* or injur* or pain* or sprain* or strain*).mp.
40. (coccygodyn* or coccalg* or coccygalg*).mp.
41. dorsalg*.mp.
42. lumbago*.mp.
43. lumboischialg*.mp.
44. (piriformis* adj2 syndrome*).mp.
45. sacral* adj3 (pain* or facet* or (nerve adj2 root*) or osteoarth* or radicul* or stenos* or spondylo* or zygapophys* or injur* or discomfort* or dysfunction* or sore* or herniat*).mp.
46. sacro* adj3 (pain* or facet* or (nerve adj2 root*) or osteoarth* or radicul* or stenos* or spondylo* or zygapophys* or injur* or discomfort* or dysfunction* or sore* or herniat*).mp.
47. "si" adj2 (joint* adj3 (pain* or facet* or (nerve adj2 root*) or osteoarth* or radicul* or stenos* or spondylo* or zygapophys* or injur* or discomfort* or dysfunction* or sore* or herniat*)).mp.
48. sacrococcy* adj2 (ach* or injur* or pain* or sprain* or strain*).mp.
49. sacrum* adj2 (ach* or injur* or pain* or sprain* or strain*).mp.
50. sciatic*.mp.
51. stenos* adj2 (spine* or spinal* or vertebral*).mp.
52. (spine* or spinal*) adj2 osteoarthr*.mp.
53. spine* adj3 (condition* or diseas* or disabilit* or disorder* or degenerat* or pain* or stenos*).mp.
54. spinal* adj3 (condition* or diseas* or disabilit* or disorder* or degenerat* or pain* or stenos*).mp.
55. spondylo*.mp.
56. tailbone* adj3 (ach* or injur* or pain* or sprain* or strain*).mp.
57. vertebr* adj3 (ach* or injur* or pain* or sprain* or strain*).mp
58. poly-radicul* or polyradicul*.mp.
59. neuropath* adj2 (lumbar* or lumbo* or sacral* or sacro* or (low* adj2 back) or low-back* or lower-back* or spine* or spinal* or L1 or L2 or L3 or L4 or L5).mp.
60. radiculopath* adj3 (lumbar* or lumbo* or sacral* or sacro* or (low* adj2 back) or low-back* or lower-back* or spine* or spinal* or L1 or L2 or L3 or L4 or L5).mp.
61. radiating* adj3 (lumbar* or lumbo* or sacral* or sacro* or (low* adj2 back) or low-back* or lower-back* or spine* or spinal* or L1 or L2 or L3 or L4 or L5).mp.
62. radicular* adj3 (lumbar* or lumbo* or sacral* or sacro* or (low* adj2 back) or low-back* or lower-back* or spine* or spinal* or L1 or L2 or L3 or L4 or L5).mp.
63. lumborum* adj3 (ach* or injur* or pain* or sprain* or strain*).mp.
64. longissimus* adj3 (ach* or injur* or pain* or sprain* or strain*).mp.
65. (erector adj2 spin*) adj3 (ach* or injur* or pain* or sprain* or strain*).mp.
66. synovial* adj2 cyst*.mp.
67. thoracolumbar* adj3 (pain* or facet* or (nerve* adj2 root*) or osteoarthr* or radicul* or stenos* or spondylo* or zygapohys* or injur* or trauma* or discomfort* or dysfunction* or sore* or herniat*).mp.
68. thoraco-lumbar* adj3 (pain* or facet* or (nerve* adj2 root*) or osteoarthr* or radicul* or stenos* or spondylo* or zygapohys* or injur* or trauma* or discomfort* or dysfunction* or sore* or herniat*).mp.
69. curvatur* adj2 (spine* or spinal*).mp.
70. (pathol* adj2 (lumbar* or (low* adj2 back) or low-back* or (lower* adj2 back) or lower-back* or thoracolumbar* or thoraco-lumbar* or intervertebral* or lumbosacral* or lumbo-sacral* or sacral* or sacro-iliac* or sacroiliac*)).mp.
71. or/1-70
72. Patient Education as Topic/
73. exp Health Education/
74. Health Knowledge, Attitudes, Practice/
75. exp Health Promotion/
76. Education/
77. Counseling/
78. Patient Education Handout/
79. Pamphlets/
80. Books/
81. Internet/
82. Social Media/
83. Bed Rest/
84. exp Activities of Daily Living/
85. Self-Management/
86. Self Care/
87. Program Evaluation/
88. advice*.mp.
89. coach*.mp.
90. counsel*.mp.
91. stay* adj2 activ*.mp.
92. bed* adj2 rest*.mp.
93. reassur*.mp.
94. ((self* adj2 manag*) or self-manag*).mp.
95. ((self* adj2 care*) or self-care*).mp.
96. ((self* adj help*) or self-help*).mp.
97. back adj school*.mp.
98. educat*.mp.
99. book*.mp.
100. pamphlet*.mp.
101. handout*.mp.
102. leaflet*.mp.
103. information* adj2 (online* or written* or oral* or verbal* or digtal* or health*).mp.
104. (website* or web-site* or web-base* or (web* adj2 page*) or (web* adj2 application*) or (web* adj2 interfac*)).mp.
105. (email* or e-mail*).mp.
106. internet*.mp.
107. (app or apps or application*).mp.
108. (phone or phones or cellphone* or smartphone or iphone*).mp.
109. (text* adj2 messag*).mp.
110. (instruction* adj2 video*).mp.
111. (evaluat* adj2 (program* or process*)).mp.
112. (explain* adj2 pain*).mp.
113. (normal* adj2 activit*).mp.
114. (day* adj2 activit*).mp.
115. (ordinary* adj activit*).mp.
116. (daily* adj2 activit*).mp.
117. neurophysio*.mp.
118. (pain* adj2 neuroscience).mp.
119. (therapeutic* adj2 neuroscience).mp.
120. (pain* adj2 biolog*).mp.
121. (pain* adj2 science*).mp.
122. neurobio*.mp.
123. neuroscience*.mp.
124. or/ 72-123
125. exp Randomized Controlled Trial/
126. exp Randomized Controlled Trials as Topic/
127. Controlled Clinical Trial/
128. exp Controlled Clinical Trials as Topic/
129. exp Clinical Trials as Topic/
130. exp Clinical Trial/
131. Double-Blind Method/
132. Single-Blind Method/
133. Cross-Over Trials/
134. Placebos/
135. Placebo Effect/
136. Random Allocation/
137. random*.mp.
138. clinical* adj2 trial*mp.
139. controlled* adj2 (trial*).mp.
140. blind* adj2 (doubl* or singl*).mp.
141. placebo*.mp.
142. (crossover* or cross-over*).mp.
143. randomized controlled trial.pt.
144. controlled clinical trial.pt.
145. clinical trial.pt.
146. or/ 125-145
147. 71 AND 124 AND 146
148. exp Animals/
149. exp Humans/
150. 148 NOT 149
151. Limit 147 NOT 150
152. (comment or clinical conference or congress or consensus development conference or editorial or letter or review or systematic review or guideline or practice guideline or case reports).pt.
153. Limit 151 NOT 152
154. Limit 153 to dt=20200901-20220309
155. Limit 153 to rd=20200901-20220309
156. 154 or 155

**B. Database & Platform:** CINAHL (EBSCO)

**Years of search:** September 1, 2020 - March 9, 2022

**Date search run:** March 9, 2022

**Number of records retrieved:** 268

**Search Strategy:**

1. MH Low Back Pain
2. MH Back Pain+
3. MH Back Injuries
4. MH Intervertebral Disc Displacement
5. MH Intervertebral Disc/IN
6. MH Lumbar Vertebrae/IN
7. MH Lumbosacral Plexus/IN
8. MH Coccyx/IN
9. MH Osteoarthritis, Spine
10. MH Osteoarthritis
11. MH Piriformis Muscles/IN
12. MH Polyradiculopathy/
13. MH Sacroiliac Joint/IN
14. MH Sciatica
15. MH Spinal Curvatures
16. MH Spinal Diseases
17. MH Spinal Injuries
18. MH Spinal Stenosis
19. MH Spondylolysis+
20. MH Spondylosis
21. MH Synovial Cyst
22. MH Zygaphophyseal Joint/IN
23. TI ((low* n2 (back n2 pain*)) or (low-back* n2 pain*) or (lower-back* n2 pain*) or (low* n2 back-pain*)) or AB ((low* n2 (back n2 pain*)) or (low-back* n2 pain*) or (lower-back* n2 pain*) or (low* n2 back-pain*))
24. TI ((low* n2 (back n2 injur*)) or (low-back* n2 injur*) or (lower-back* n2 injur*) or (low* n2 back-injur*)) or AB ((low* n2 (back n2 injur*)) or (low-back* n2 injur*) or (lower-back* n2 injur*) or (low* n2 back-injur*))
25. TI ((low* n2 (back n2 trauma*)) or (low-back n2 trauma*) or (lower-back* n2 trauma*) or (low* n2 back-trauma*)) or AB ((low* n2 (back n2 trauma*)) or (low-back n2 trauma*) or (lower-back* n2 trauma*) or (low* n2 back-trauma*))
26. TI ((low* n2 (trunk n2 pain*)) or (lower-trunk* n2 pain*) or (low* n2 trunk-pain*)) or AB ((low* n2 (trunk n2 pain*)) or (lower-trunk* n2 pain*) or (low* n2 trunk-pain*))
27. TI lumbar* n3 (disc* n3 (extru* or degenerat* or displac* or herniat* or prolaps* or sequestered or slipped or protru* or avuls*)) or AB lumbar* n3 (disc* n3 (extru* or degenerat* or displac* or herniat* or prolaps* or sequestered or slipped or protru* or avuls*))
28. TI lumbar* n3 (disk* n3 (extru* or degenerat* or displac* or herniat* or prolaps* or sequestered or slipped or protru* or avuls*)) or AB lumbar* n3 (disk* n3 (extru* or degenerat* or displac* or herniat* or prolaps* or sequestered or slipped or protru* or avuls*))
29. TI lumbar* n3 (pain* or facet* or (nerve n2 root*) or osteoarth* or radicul* or stenos* or spondylo* or zygapophys* or injur* or discomfort* or dysfunction* or sore* or herniat*) or AB lumbar* n3 (pain* or facet* or (nerve n2 root*) or osteoarth* or radicul* or stenos* or spondylo* or zygapophys* or injur* or discomfort* or dysfunction* or sore* or herniat*)
30. TI lumbo* n3 (pain* or facet* or (nerve n2 root*) or osteoarth* or radicul* or stenos* or spondylo* or zygapophys* or injur* or discomfort* or dysfunction* or sore* or herniat*) or AB lumbo* n3 (pain* or facet* or (nerve n2 root*) or osteoarth* or radicul* or stenos* or spondylo* or zygapophys* or injur* or discomfort* or dysfunction* or sore* or herniat*)
31. TI back n3 (ach* or injur* or pain* or sprain* or strain* or disorder*) or AB back n3 (ach* or injur* or pain* or sprain* or strain* or disorder
32. TI backach* or AB backach*
33. TI back-pain* or AB back-pain*
34. TI intervertebral* n3 (disc* n3 (extru* or degenerat* or displac* or herniat* or prolaps* or sequestered or slipped or protru* or avuls*)) or AB intervertebral* n3 (disc* n3 (extru* or degenerat* or displac* or herniat* or prolaps* or sequestered or slipped or protru* or avuls*))
35. TI intervertebral* n3 (disk* n3 (extru* or degenerat* or displac* or herniat* or prolaps* or sequestered or slipped or protru* or avuls*)) or AB intervertebral* n3 (disk* n3 (extru* or degenerat* or displac* or herniat* or prolaps* or sequestered or slipped or protru* or avuls*))
36. TI coccy* n2 (ach* or injur* or pain* or sprain* or strain*) or AB coccy* n2 (ach* or injur* or pain* or sprain* or strain*)
37. TI (coccygodyn* or coccalg* or coccygalg*) or AB (coccygodyn* or coccalg* or coccygalg*)
38. TI dorsalg* or AB dorsalg*
39. TI lumbago* or AB lumbago*
40. TI lumboischialg* or AB lumboischialg*
41. TI (piriformis* n2 syndrome*) or AB (piriformis* n2 syndrome*)
42. TI sacral* n3 (pain* or facet* or (nerve n2 root*) or osteoarth* or radicul* or stenos* or spondylo* or zygapophys* or injur* or discomfort* or dysfunction* or sore* or herniat*) or AB sacral* n3 (pain* or facet* or (nerve n2 root*) or osteoarth* or radicul* or stenos* or spondylo* or zygapophys* or injur* or discomfort* or dysfunction* or sore* or herniat*)
43. TI sacro* n3 (pain* or facet* or (nerve n2 root*) or osteoarth* or radicul* or stenos* or spondylo* or zygapophys* or injur* or discomfort* or dysfunction* or sore* or herniat*) or AB sacro* n3 (pain* or facet* or (nerve n2 root*) or osteoarth* or radicul* or stenos* or spondylo* or zygapophys* or injur* or discomfort* or dysfunction* or sore* or herniat*)
44. TI "si" n2 (joint* n3 (pain* or facet* or (nerve n2 root*) or osteoarth* or radicul* or stenos* or spondylo* or zygapophys* or injur* or discomfort* or dysfunction* or sore* or herniat*)) or AB "si" n2 (joint* n3 (pain* or facet* or (nerve n2 root*) or osteoarth* or radicul* or stenos* or spondylo* or zygapophys* or injur* or discomfort* or dysfunction* or sore* or herniat*))
45. TI sacrococcy* n2 (ach* or injur* or pain* or sprain* or strain*) or AB sacrococcy* n2 (ach* or injur* or pain* or sprain* or strain*)
46. TI sacrum* n2 (ach* or injur* or pain* or sprain* or strain*) or AB sacrum* n2 (ach* or injur* or pain* or sprain* or strain*)
47. TI sciatic* or sciatic*
48. TI stenos* n2 (spine* or spinal* or vertebral*) or AB stenos* n2 (spine* or spinal* or vertebral*)
49. TI (spine* or spinal*) n2 osteoarthr* or AB (spine* or spinal*) n2 osteoarthr*
50. TI spine* n3 (condition* or diseas* or disabilit* or disorder* or degenerat* or pain* or stenos*) or AB spine* n3 (condition* or diseas* or disabilit* or disorder* or degenerat* or pain* or stenos*)
51. TI spinal* n3 (condition* or diseas* or disabilit* or disorder* or degenerat* or pain* or stenos*) or AB spinal* n3 (condition* or diseas* or disabilit* or disorder* or degenerat* or pain* or stenos*)
52. TI spondylo* or AB spondylo*
53. TI tailbone* n3 (ach* or injur* or pain* or sprain* or strain*) or AB tailbone* n3 (ach* or injur* or pain* or sprain* or strain*)
54. TI vertebr* n3 (ach* or injur* or pain* or sprain* or strain*) or AB vertebr* n3 (ach* or injur* or pain* or sprain* or strain*)
55. TI (poly-radicul* or polyradicul*) or AB (poly-radicul* or polyradicul*)
56. TI neuropath* n2 (lumbar* or lumbo* or sacral* or sacro* or (low* n2 back) or low-back* or lower-back* or spine* or spinal* or L1 or L2 or L3 or L4 or L5) or AB neuropath* n2 (lumbar* or lumbo* or sacral* or sacro* or (low* n2 back) or low-back* or lower-back* or spine* or spinal* or L1 or L2 or L3 or L4 or L5)
57. TI radiculopath* n3 (lumbar* or lumbo* or sacral* or sacro* or (low* n2 back) or low-back* or lower-back* or spine* or spinal* or L1 or L2 or L3 or L4 or L5) or AB radiculopath* n3 (lumbar* or lumbo* or sacral* or sacro* or (low* n2 back) or low-back* or lower-back* or spine* or spinal* or L1 or L2 or L3 or L4 or L5)
58. TI radiating* n3 (lumbar* or lumbo* or sacral* or sacro* or (low* n2 back) or low-back* or lower-back* or spine* or spinal* or L1 or L2 or L3 or L4 or L5) or AB radiating* n3 (lumbar* or lumbo* or sacral* or sacro* or (low* n2 back) or low-back* or lower-back* or spine* or spinal* or L1 or L2 or L3 or L4 or L5)
59. TI radicular* n3 (lumbar* or lumbo* or sacral* or sacro* or (low* n2 back) or low-back* or lower-back* or spine* or spinal* or L1 or L2 or L3 or L4 or L5) or AB radicular* n3 (lumbar* or lumbo* or sacral* or sacro* or (low* n2 back) or low-back* or lower-back* or spine* or spinal* or L1 or L2 or L3 or L4 or L5)
60. TI lumborum* n3 (ach* or injur* or pain* or sprain* or strain*) or AB lumborum* n3 (ach* or injur* or pain* or sprain* or strain*)
61. TI longissimus* n3 (ach* or injur* or pain* or sprain* or strain*) or AB longissimus* n3 (ach* or injur* or pain* or sprain* or strain*)
62. TI (erector n2 spin*) n3 (ach* or injur* or pain* or sprain* or strain*) or AB erector n2 spin*) n3 (ach* or injur* or pain* or sprain* or strain*)
63. TI synovial* n2 cyst* or AB synovial* n2 cyst*
64. TI thoracolumbar* n3 (pain* or facet* or (nerve* n2 root*) or osteoarthr* or radicul* or stenos* or spondylo* or zygapohys* or injur* or trauma* or discomfort* or dysfunction* or sore* or herniat*) or AB thoracolumbar* n3 (pain* or facet* or (nerve* n2 root*) or osteoarthr* or radicul* or stenos* or spondylo* or zygapohys* or injur* or trauma* or discomfort* or dysfunction* or sore* or herniat*)
65. TI thoraco-lumbar* n3 (pain* or facet* or (nerve* n2 root*) or osteoarthr* or radicul* or stenos* or spondylo* or zygapohys* or injur* or trauma* or discomfort* or dysfunction* or sore* or herniat*) or AB thoraco-lumbar* n3 (pain* or facet* or (nerve* n2 root*) or osteoarthr* or radicul* or stenos* or spondylo* or zygapohys* or injur* or trauma* or discomfort* or dysfunction* or sore* or herniat*)
66. TI curvatur* n2 (spine* or spinal*) or AB curvatur* n2 (spine* or spinal*)
67. TI (pathol* n2 (lumbar* or (low* n2 back) or low-back* or (lower* n2 back) or lower-back* or thoracolumbar* or thoraco-lumbar* or intervertebral* or lumbosacral* or lumbo-sacral* or sacral* or sacro-iliac* or sacroiliac*)) or AB (pathol* n2 (lumbar* or (low* n2 back) or low-back* or (lower* n2 back) or lower-back* or thoracolumbar* or thoraco-lumbar* or intervertebral* or lumbosacral* or lumbo-sacral* or sacral* or sacro-iliac* or sacroiliac*))
68. or/1-67
69. MH Patient Education
70. MH Health Education
71. MH Education
72. MH Health Promotion
73. MH Counseling+
74. MH Mentorship
75. MH Pamphlets
76. MH Books
77. MH Internet
78. MH Social Media
79. MH Bed Rest/
80. MH Activities of Daily Living+
81. MH Self-Management
82. MH Self Care
83. MH Program Evaluation
84. TI (advice* or advis*) or AB (advice* or advis*)
85. TI coach* or AB coach*
86. TI counsel* or AB counsel*
87. TI (stay* n2 activ*) or AB (stay* n2 activ*)
88. TI (bed* n2 rest*) or AB (bed* n2 rest*)
89. TI reassur* or AB reassur*
90. TI ((self* n2 manag*) or self-manag*) or AB ((self* n2 manag*) or self-manag*)
91. TI ((self* n2 care*) or self-care*) or AB ((self* n2 care*) or self-care*)
92. TI ((self* n2 help*) or self-help*) or AB ((self* n2 help*) or self-help*)
93. TI (back n2 school*) or AB (back n2 school*)
94. TI educat* or AB educat*
95. TI book* or AB book*
96. TI pamphlet* or AB pamphlet*
97. TI handout* or AB handout*
98. TI leaflet* or AB leaflet*
99. TI information* n2 (online* or written* or oral* or verbal* or digtal* or health*) or AB information* n2 (online* or written* or oral* or verbal* or digtal* or health*)
100. TI (website* or web-site* or web-base* or (web* n2 page*) or (web* n2 application*) or (web* n2 interfac*)) or AB (website* or web-site* or web-base* or (web* n2 page*) or (web* n2 application*) or (web* n2 interfac*))
101. TI (email* or e-mail*) or AB (email* or e-mail*)
102. TI internet* or AB internet*
103. TI (app or apps or application*) or AB (app or apps or application*)
104. TI (phone or phones or cellphone* or smartphone or iphone* or i-phone*) or AB (phone or phones or cellphone* or smartphone or iphone* or i-phone*)
105. TI (text* n2 messag*) or AB (text* n2 messag*)
106. TI (instruction* n2 video*) or AB (instruction* n2 video*)
107. TI (evaluat* n2 (program* or process*)) or AB (evaluat* n2 (program* or process*))
108. TI (explain* n2 pain*) or AB (explain* n2 pain*)
109. TI (normal* n2 activit*) or AB (normal* n2 activit*)
110. TI (day* n2 activit*) or AB (day* n2 activit*)
111. TI (ordinary* n2 activit*) or AB (ordinary* n2 activit*)
112. TI (daily* n2 activit*) or AB (daily* n2 activit*)
113. TI neurophysio* or AB neurophysio*
114. TI (pain* n2 neuroscience) or AB (pain* n2 neuroscience)
115. TI (therapeutic* n2 neuroscience) or AB (therapeutic* n2 neuroscience)
116. TI (pain* n2 biolog*) or AB (pain* n2 biolog*)
117. TI (pain* n2 science*) or AB (pain* n2 science*)
118. TI neurobio* or AB neurobio*
119. TI neuroscience* or AB neuroscience*)
120. or/ 69-119
121. MH Randomized Controlled Trials+
122. MH Clinical Trials+
123. MH Double-Blind Studies
124. MH Single-Blind Studies
125. MH Crossover Design
126. MH Placebos
127. MH Placebo Effect
128. MH Random Assignment
129. TI random* or AB random*
130. TI clinical* n2 (trial* or study* or studies*) or AB clinical* n2 (trial* or study* or studies*)
131. TI controlled* n2 (trial* or study* or studies*) or AB controlled* n2 (trial* or study* or studies*)
132. TI blind* n2 (doubl* or singl*) or AB blind* n2 (doubl* or singl*)
133. TI placebo* or AB placebo*
134. TI (crossover* or cross-over*) or AB (crossover* or cross-over*)
135. PT randomized controlled trial
136. PT controlled clinical trial
137. PT clinical trial
138. or/ 121-137
139. 68 AND 120 AND 138
140. MH Animals+
141. MH Human
142. 140 NOT 141
143. Limit 139 NOT 142
144. 143 NOT PT (abstract or brief item or book review or case study or commentary or doctoral dissertation or editorial or letter or practice guidelines or proceedings or review or systematic review)
145. 144 AND EM=20200901-20220309
146. 144 AND RD=20200901-20220309
147. 145 OR 146

**C.** **Database & Platform:** EMBASE (Ovid)

**Years of search:** September 1, 2020 - March 9, 2022

**Date search run:** March 9, 2022

**Number of records retrieved:** 166

**Search Strategy:**

1. Low Back Pain/
2. exp Backache/
3. Intervertebral Disc Degeneration/
4. Intervertebral Disk Hernia/
5. Lumbar Disk Hernia/
6. Coccyx/
7. Spondylosis/
8. Spondylolysis/
9. Osteoarthritis/
10. Lumbar Spinal Stenosis/
11. Synovial Cyst/
12. Piriformis Syndrome/
13. Sciatica/
14. ((low* adj2 (back adj2 pain*)) or (low-back* adj2 pain*) or (lower-back* adj2 pain*) or (low* adj2 back-pain*)).mp.
15. ((low* adj2 (back adj2 injur*)) or (low-back* adj2 injur*) or (lower-back* adj2 injur*) or (low* adj2 back-injur*)).mp.
16. ((low* adj2 (back adj2 trauma*)) or (low-back adj2 trauma*) or (lower-back* adj2 trauma*) or (low* adj2 back-trauma*)).mp.
17. ((low* adj2 (trunk adj2 pain*)) or (lower-trunk* adj2 pain*) or (low* adj2 trunk-pain*)).mp.
18. lumbar* adj3 (disc* adj3 (extru* or degenerat* or displac* or herniat* or prolaps* or sequestered or slipped or protru* or avuls*)).mp.
19. lumbar* adj3 (disk* adj3 (extru* or degenerat* or displac* or herniat* or prolaps* or sequestered or slipped or protru* or avuls*)).mp.
20. lumbar* adj3 (pain* or facet* or (nerve adj2 root*) or osteoarth* or radicul* or stenos* or spondylo* or zygapophys* or injur* or discomfort* or dysfunction* or sore* or herniat*).mp.
21. lumbo* adj3 (pain* or facet* or (nerve adj2 root*) or osteoarth* or radicul* or stenos* or spondylo* or zygapophys* or injur* or discomfort* or dysfunction* or sore* or herniat*).mp.
22. back adj3 (ach* or injur* or pain* or sprain* or strain* or disorder*).mp.
23. backach*.mp.
24. back-pain*.mp.
25. intervertebral* adj3 (disc* adj3 (extru* or degenerat* or displac* or herniat* or prolaps* or sequestered or slipped or protru* or avuls*)).mp.
26. intervertebral* adj3 (disk* adj3 (extru* or degenerat* or displac* or herniat* or prolaps* or sequestered or slipped or protru* or avuls*)).mp.
27. coccy* adj2 (ach* or injur* or pain* or sprain* or strain*).mp.
28. (coccygodyn* or coccalg* or coccygalg*).mp.
29. dorsalg*.mp.
30. lumbago*.mp.
31. lumboischialg*.mp.
32. (piriformis* adj2 syndrome*).mp.
33. sacral* adj3 (pain* or facet* or (nerve adj2 root*) or osteoarth* or radicul* or stenos* or spondylo* or zygapophys* or injur* or discomfort* or dysfunction* or sore* or herniat*).mp.
34. sacro* adj3 (pain* or facet* or (nerve adj2 root*) or osteoarth* or radicul* or stenos* or spondylo* or zygapophys* or injur* or discomfort* or dysfunction* or sore* or herniat*).mp.
35. "si" adj2 (joint* adj3 (pain* or facet* or (nerve adj2 root*) or osteoarth* or radicul* or stenos* or spondylo* or zygapophys* or injur* or discomfort* or dysfunction* or sore* or herniat*)).mp.
36. sacrococcy* adj2 (ach* or injur* or pain* or sprain* or strain*).mp.
37. sacrum* adj2 (ach* or injur* or pain* or sprain* or strain*).mp.
38. sciatic*.mp.
39. stenos* adj2 (spine* or spinal* or vertebral*).mp.
40. (spine* or spinal*) adj2 osteoarthr*.mp.
41. spine* adj3 (condition* or diseas* or disabilit* or disorder* or degenerat* or pain* or stenos*).mp.
42. spinal* adj3 (condition* or diseas* or disabilit* or disorder* or degenerat* or pain* or stenos*).mp.
43. spondylo*.mp.
44. tailbone* adj3 (ach* or injur* or pain* or sprain* or strain*).mp.
45. vertebr* adj3 (ach* or injur* or pain* or sprain* or strain*).mp
46. poly-radicul* or polyradicul*.mp.
47. neuropath* adj2 (lumbar* or lumbo* or sacral* or sacro* or (low* adj2 back) or low-back* or lower-back* or spine* or spinal* or L1 or L2 or L3 or L4 or L5).mp.
48. radiculopath* adj3 (lumbar* or lumbo* or sacral* or sacro* or (low* adj2 back) or low-back* or lower-back* or spine* or spinal* or L1 or L2 or L3 or L4 or L5).mp.
49. radiating* adj3 (lumbar* or lumbo* or sacral* or sacro* or (low* adj2 back) or low-back* or lower-back* or spine* or spinal* or L1 or L2 or L3 or L4 or L5).mp.
50. radicular* adj3 (lumbar* or lumbo* or sacral* or sacro* or (low* adj2 back) or low-back* or lower-back* or spine* or spinal* or L1 or L2 or L3 or L4 or L5).mp.
51. lumborum* adj3 (ach* or injur* or pain* or sprain* or strain*).mp.
52. longissimus* adj3 (ach* or injur* or pain* or sprain* or strain*).mp.
53. (erector adj2 spin*) adj3 (ach* or injur* or pain* or sprain* or strain*).mp.
54. synovial* adj2 cyst*.mp.
55. thoracolumbar* adj3 (pain* or facet* or (nerve* adj2 root*) or osteoarthr* or radicul* or stenos* or spondylo* or zygapohys* or injur* or trauma* or discomfort* or dysfunction* or sore* or herniat*).mp.
56. thoraco-lumbar* adj3 (pain* or facet* or (nerve* adj2 root*) or osteoarthr* or radicul* or stenos* or spondylo* or zygapohys* or injur* or trauma* or discomfort* or dysfunction* or sore* or herniat*).mp.
57. curvatur* adj2 (spine* or spinal*).mp.
58. (pathol* adj2 (lumbar* or (low* adj2 back) or low-back* or (lower* adj2 back) or lower-back* or thoracolumbar* or thoraco-lumbar* or intervertebral* or lumbosacral* or lumbo-sacral* or sacral* or sacro-iliac* or sacroiliac*)).mp.
59. or/1-58
60. Patient Education/
61. exp Health Education/
62. exp Health Promotion/
63. Education/
64. exp Counseling/
65. Mentoring/
66. exp Publication/
67. exp Book/
68. Internet/
69. Social Media/
70. exp Mobile Phone/
71. Mobile Health Application/
72. Mobile Application/
73. Self-Care Software/
74. exp Mass Communication/
75. exp Distance Learning/
76. Patient Information/
77. Bed Rest/
78. Daily Life Activity/
79. Self Care/
80. (advice* or advis*).mp.
81. coach*.mp.
82. counsel*.mp.
83. stay* adj2 activ*.mp.
84. bed* adj2 rest*.mp.
85. reassur*.mp.
86. ((self* adj2 manag*) or self-manag*).mp.
87. ((self* adj2 care*) or self-care*).mp.
88. ((self* adj help*) or self-help*).mp.
89. back adj school*.mp.
90. educat*.mp.
91. book*.mp.
92. pamphlet*.mp.
93. handout*.mp.
94. leaflet*.mp.
95. information* adj2 (online* or written* or oral* or verbal* or digtal* or health* or face-to-face).mp.
96. (website* or web-site* or web-base* or (web* adj2 page*) or (web* adj2 application*) or (web* adj2 interfac*)).mp.
97. (email* or e-mail*).mp.
98. internet*.mp.
99. (app or apps or application*).mp.
100. (phone or phones or cellphone* or smartphone or iphone*).mp.
101. (text* adj2 messag*).mp.
102. (instruction* adj2 video*).mp.
103. (evaluat* adj2 (program* or process*)).mp.
104. (explain* adj2 pain*).mp.
105. (normal* adj2 activit*).mp.
106. (day* adj2 activit*).mp.
107. (ordinary* adj activit*).mp.
108. (daily* adj2 activit*).mp.
109. neurophysio*.mp.
110. (pain* adj2 neuroscience).mp.
111. (therapeutic* adj2 neuroscience).mp.
112. (pain* adj2 biolog*).mp.
113. (pain* adj2 science*).mp.
114. neurobio*.mp.
115. neuroscience*.mp.
116. or/ 60-115
117. exp Randomized Controlled Trial/
118. exp Randomized Controlled Trial (Topic)/
119. Controlled Clinical Trial/
120. exp Controlled Clinical Trial (Topic)/
121. exp Clinical Trial (Topic)/
122. exp Clinical Trial/
123. Double-Blind Procedure/
124. Single-Blind Procedure/
125. Crossover Procedure/
126. Placebo/
127. Placebo Effect/
128. Randomization/
129. random*.mp.
130. clinical* adj2 trial*mp.
131. controlled* adj2 (trial*).mp.
132. blind* adj2 (doubl* or singl*).mp.
133. placebo*.mp.
134. (crossover* or cross-over*).mp.
135. or/ 117-134
136. 59 AND 116 AND 135
137. exp Animal/
138. exp Human/
139. 137 NOT 138
140. Limit 136 NOT 139
141. (books or chapter or conference abstract or conference paper or conference review or review or editorial or letter).pt.
142. Limit 140 NOT 141
143. Limit 142 to dd=20200901-20220309
144. Limit 142 to rd=20200901-20220309
145. 143 or 144

**D. Database & Platform:** Cochrane Central Register of Controlled Trials (Wiley)

**Years of search:** September 2020 – March 2022

**Date search run:** March 9, 2022

**Number of records retrieved:** 650

**Search Strategy:**

#1 MeSH descriptor: [Back Injuries] explode all trees

#2 MeSH descriptor: [Back Pain] explode all trees

#3 MeSH descriptor: [Low Back Pain] this term only

#4 MeSH descriptor: [Osteoarthritis] this term only

#5 MeSH descriptor: [Osteoarthritis, Spine] this term only

#6 MeSH descriptor: [Piriformis Muscle Syndrome] this term only

#7 MeSH descriptor: [Polyradiculopathy] this term only

#8 MeSH descriptor: [Spinal Diseases] explode all trees

#9 MeSH descriptor: [Synovial Cyst] this term only

#10 MeSH descriptor: [Back Muscles] this term only and with qualifier(s): [injuries - IN]

#11 MeSH descriptor: [Intervertebral Disc Degeneration] this term only

#12 MeSH descriptor: [Intervertebral Disc Displacement] this term only

#13 MeSH descriptor: [Intervertebral Disc] this term only and with qualifier(s): [injuries - IN]

#14 MeSH descriptor: [Lumbar Vertebrae] this term only and with qualifier(s): [injuries - IN]

#15 MeSH descriptor: [Lumbosacral Plexus] this term only and with qualifier(s): [injuries - IN]

#16 MeSH descriptor: [Sacroiliac Joint] this term only and with qualifier(s): [injuries - IN]

#17 MeSH descriptor: [Sacrum] this term only and with qualifier(s): [injuries - IN]

#18 MeSH descriptor: [Lumbosacral Region] this term only and with qualifier(s): [injuries - IN]

#19 MeSH descriptor: [Zygapophyseal Joint] this term only and with qualifier(s): [injuries - IN]

#20 MeSH descriptor: [Sciatica] this term only

#21 MeSH descriptor: [Spinal Injuries] this term only

#22 MeSH descriptor: [Spinal Stenosis] this term only

#23 MeSH descriptor: [Spondylolysis] explode all trees

#24 (((low* near/2 (back near/2 pain*)) or (low-back* near/2 pain*) or (lower-back* near/2 pain*) or (low* near/2 back-pain*))):ti OR (((low* near/2 (back near/2 pain*)) or (low-back* near/2 pain*) or (lower-back* near/2 pain*) or (low* near/2 back-pain*))):ab

#25 (((low* near/2 (back near/2 injur*)) or (low-back* near/2 injur*) or (lower-back* near/2 injur*) or (low* near/2 back-injur*))):ti OR (((low* near/2 (back near/2 injur*)) or (low-back* near/2 injur*) or (lower-back* near/2 injur*) or (low* near/2 back-injur*))):ab

#26 (((low* near/2 (back near/2 trauma*)) or (low-back near/2 trauma*) or (lower-back* near/2 trauma*) or (low* near/2 back-trauma*))):ti OR (((low* near/2 (back near/2 trauma*)) or (low-back near/2 trauma*) or (lower-back* near/2 trauma*) or (low* near/2 back-trauma*))):ab

#27 (((low* near/2 (trunk near/2 pain*)) or (lower-trunk* near/2 pain*) or (low* near/2 trunk-pain*))):ti OR (((low* near/2 (trunk near/2 pain*)) or (lower-trunk* near/2 pain*) or (low* near/2 trunk-pain*))):ab

#28 (lumbar* near/3 (disc* near/3 (extru* or degenerat* or displac* or herniat* or prolaps* or sequestered or slipped or protru* or avuls*))):ti OR (lumbar* near/3 (disc* near/3 (extru* or degenerat* or displac* or herniat* or prolaps* or sequestered or slipped or protru* or avuls*))):ab

#29 (lumbar* near/3 (disk* near/3 (extru* or degenerat* or displac* or herniat* or prolaps* or sequestered or slipped or protru* or avuls*))):ti OR (lumbar* near/3 (disk* near/3 (extru* or degenerat* or displac* or herniat* or prolaps* or sequestered or slipped or protru* or avuls*))):ab

#30 (lumbar* near/3 (pain* or facet* or (nerve near/2 root*) or osteoarth* or radicul* or stenos* or spondylo* or zygapophys* or injur* or discomfort* or dysfunction* or sore* or herniat*)):ti OR (lumbar* near/3 (pain* or facet* or (nerve near/2 root*) or osteoarth* or radicul* or stenos* or spondylo* or zygapophys* or injur* or discomfort* or dysfunction* or sore* or herniat*)):ab

#31 (lumbo* near/3 (pain* or facet* or (nerve near/2 root*) or osteoarth* or radicul* or stenos* or spondylo* or zygapophys* or injur* or discomfort* or dysfunction* or sore* or herniat*)):ti OR (lumbo* near/3 (pain* or facet* or (nerve near/2 root*) or osteoarth* or radicul* or stenos* or spondylo* or zygapophys* or injur* or discomfort* or dysfunction* or sore* or herniat*)):ab

#32 (back near/3 (ach* or injur* or pain* or sprain* or strain* or disorder*)):ti OR (back near/3 (ach* or injur* or pain* or sprain* or strain* or disorder*)):ab

#33 (backach*):ti OR (backach*):ab

#34 (back-pain*):ti OR (back-pain*):ab

#35 (intervertebral* near/3 (disc* near/3 (extru* or degenerat* or displac* or herniat* or prolaps* or sequestered or slipped or protru* or avuls*))):ti OR (intervertebral* near/3 (disc* near/3 (extru* or degenerat* or displac* or herniat* or prolaps* or sequestered or slipped or protru* or avuls*))):ab

#36 (intervertebral* near/3 (disk* near/3 (extru* or degenerat* or displac* or herniat* or prolaps* or sequestered or slipped or protru* or avuls*))):ti OR (intervertebral* near/3 (disk* near/3 (extru* or degenerat* or displac* or herniat* or prolaps* or sequestered or slipped or protru* or avuls*))):ab

#37 (coccy* near/2 (ach* or injur* or pain* or sprain* or strain*)):ti OR (coccy* near/2 (ach* or injur* or pain* or sprain* or strain*)):ab

#38 ((coccygodyn* or coccalg* or coccygalg*)):ti OR ((coccygodyn* or coccalg* or coccygalg*)):ab

#39 (dorsalg* or lumbago* or lumboischialg*):ti OR (dorsalg* or lumbago* or lumboischialg*):ab

#40 ((piriformis* near/2 syndrome*)):ti OR ((piriformis* near/2 syndrome*)):ab

#41 (sacral* near/3 (pain* or facet* or (nerve near/2 root*) or osteoarth* or radicul* or stenos* or spondylo* or zygapophys* or injur* or discomfort* or dysfunction* or sore* or herniat*)):ti OR (sacral* near/3 (pain* or facet* or (nerve near/2 root*) or osteoarth* or radicul* or stenos* or spondylo* or zygapophys* or injur* or discomfort* or dysfunction* or sore* or herniat*)):ab

#42 (sacro* near/3 (pain* or facet* or (nerve near/2 root*) or osteoarth* or radicul* or stenos* or spondylo* or zygapophys* or injur* or discomfort* or dysfunction* or sore* or herniat*)):ti OR (sacro* near/3 (pain* or facet* or (nerve near/2 root*) or osteoarth* or radicul* or stenos* or spondylo* or zygapophys* or injur* or discomfort* or dysfunction* or sore* or herniat*)):ab

#43 ("si" near/2 (joint* near/3 (pain* or facet* or (nerve near/2 root*) or osteoarth* or radicul* or stenos* or spondylo* or zygapophys* or injur* or discomfort* or dysfunction* or sore* or herniat*))):ti OR ("si" near/2 (joint* near/3 (pain* or facet* or (nerve near/2 root*) or osteoarth* or radicul* or stenos* or spondylo* or zygapophys* or injur* or discomfort* or dysfunction* or sore* or herniat*))):ab

#44 (sacrococcy* near/2 (ach* or injur* or pain* or sprain* or strain*)):ti OR (sacrococcy* near/2 (ach* or injur* or pain* or sprain* or strain*)):ab

#45 (sacrum* near/2 (ach* or injur* or pain* or sprain* or strain*)):ti OR (sacrum* near/2 (ach* or injur* or pain* or sprain* or strain*)):ab

#46 (sciatic*):ti OR (sciatic*):ab ˙

#47 (stenos* near/2 (spine* or spinal* or vertebral*)):ti OR (stenos* near/2 (spine* or spinal* or vertebral*)):ab

#48 ((spine* or spinal*) near/2 osteoarthr*):ti OR ((spine* or spinal*) near/2 osteoarthr*):ab

#49 (spine* near/3 (condition* or diseas* or disabilit* or disorder* or degenerat* or pain* or stenos*)):ti OR (spine* near/3 (condition* or diseas* or disabilit* or disorder* or degenerat* or pain* or stenos*)):ab

#50 (spinal* near/3 (condition* or diseas* or disabilit* or disorder* or degenerat* or pain* or stenos*)):ti OR (spinal* near/3 (condition* or diseas* or disabilit* or disorder* or degenerat* or pain* or stenos*)):ab

#51 (spondylo*):ti OR (spondylo*):ab

#52 (tailbone* near/3 (ach* or injur* or pain* or sprain* or strain*)):ti OR (tailbone* near/3 (ach* or injur* or pain* or sprain* or strain*)):ab

#53 (vertebr* near/3 (ach* or injur* or pain* or sprain* or strain*)):ti OR (vertebr* near/3 (ach* or injur* or pain* or sprain* or strain*)):ab

#54 (poly-radicul* or polyradicul*):ti OR (poly-radicul* or polyradicul*):ab

#55 (neuropath* near/2 (lumbar* or lumbo* or sacral* or sacro* or (low* near/2 back) or low-back* or lower-back* or spine* or spinal* or L1 or L2 or L3 or L4 or L5)):ti OR (neuropath* near/2 (lumbar* or lumbo* or sacral* or sacro* or (low* near/2 back) or low-back* or lower-back* or spine* or spinal* or L1 or L2 or L3 or L4 or L5)):ab

#56 (radiculopath* near/3 (lumbar* or lumbo* or sacral* or sacro* or (low* near/2 back) or low-back* or lower-back* or spine* or spinal* or L1 or L2 or L3 or L4 or L5)):ti OR (radiculopath* near/3 (lumbar* or lumbo* or sacral* or sacro* or (low* near/2 back) or low-back* or lower-back* or spine* or spinal* or L1 or L2 or L3 or L4 or L5)):ab

#57 (radiating* near/3 (lumbar* or lumbo* or sacral* or sacro* or (low* near/2 back) or low-back* or lower-back* or spine* or spinal* or L1 or L2 or L3 or L4 or L5)):ti OR (radiating* near/3 (lumbar* or lumbo* or sacral* or sacro* or (low* near/2 back) or low-back* or lower-back* or spine* or spinal* or L1 or L2 or L3 or L4 or L5)):ab

#58 (radicular* near/3 (lumbar* or lumbo* or sacral* or sacro* or (low* near/2 back) or low-back* or lower-back* or spine* or spinal* or L1 or L2 or L3 or L4 or L5)):ti OR (radicular* near/3 (lumbar* or lumbo* or sacral* or sacro* or (low* near/2 back) or low-back* or lower-back* or spine* or spinal* or L1 or L2 or L3 or L4 or L5)):ab

#59 (lumborum* near/3 (ach* or injur* or pain* or sprain* or strain*)):ti OR (lumborum* near/3 (ach* or injur* or pain* or sprain* or strain*)):ab

#60 (longissimus* near/3 (ach* or injur* or pain* or sprain* or strain*)):ti OR (longissimus* near/3 (ach* or injur* or pain* or sprain* or strain*)):ab

#61 ((erector near/2 spin*) near/3 (ach* or injur* or pain* or sprain* or strain*)):ti OR ((erector near/2 spin*) near/3 (ach* or injur* or pain* or sprain* or strain*)):ab

#62 (synovial* near/2 cyst*):ti OR (synovial* near/2 cyst*):ab

#63 (thoracolumbar* near/3 (pain* or facet* or (nerve* near/2 root*) or osteoarthr* or radicul* or stenos* or spondylo* or zygapohys* or injur* or trauma* or discomfort* or dysfunction* or sore* or herniat*)):ti OR (thoracolumbar* near/3 (pain* or facet* or (nerve* near/2 root*) or osteoarthr* or radicul* or stenos* or spondylo* or zygapohys* or injur* or trauma* or discomfort* or dysfunction* or sore* or herniat*)):ab

#64 (thoraco-lumbar* near/3 (pain* or facet* or (nerve* near/2 root*) or osteoarthr* or radicul* or stenos* or spondylo* or zygapohys* or injur* or trauma* or discomfort* or dysfunction* or sore* or herniat*)):ti OR (thoraco-lumbar* near/3 (pain* or facet* or (nerve* near/2 root*) or osteoarthr* or radicul* or stenos* or spondylo* or zygapohys* or injur* or trauma* or discomfort* or dysfunction* or sore* or herniat*)):ab

#65 (curvatur* near/2 (spine* or spinal*)):ti OR (curvatur* near/2 (spine* or spinal*)):ab

#66 ((pathol* near/2 (lumbar* or (low* near/2 back) or low-back* or (lower* near/2 back) or lower-back* or thoracolumbar* or thoraco-lumbar* or intervertebral* or lumbosacral* or lumbo-sacral* or sacral* or sacro-iliac* or sacroiliac*))):ti OR ((pathol* near/2 (lumbar* or (low* near/2 back) or low-back* or (lower* near/2 back) or lower-back* or thoracolumbar* or thoraco-lumbar* or intervertebral* or lumbosacral* or lumbo-sacral* or sacral* or sacro-iliac* or sacroiliac*))):ab

#67 MeSH descriptor: [Patient Education as Topic] this term only

#68 MeSH descriptor: [Health Knowledge, Attitudes, Practice] this term only

#69 MeSH descriptor: [Health Promotion] this term only

#70 MeSH descriptor: [Education] this term only

#71 MeSH descriptor: [Counseling] this term only

#72 MeSH descriptor: [Mentoring] this term only

#73 MeSH descriptor: [Patient Education Handout] this term only

#74 MeSH descriptor: [Pamphlets] this term only

#75 MeSH descriptor: [Books] this term only

#76 MeSH descriptor: [Internet] this term only

#77 MeSH descriptor: [Social Media] this term only

#78 MeSH descriptor: [Bed Rest] this term only

#79 MeSH descriptor: [Activities of Daily Living] explode all trees

#80 MeSH descriptor: [Self-Management] this term only

#81 MeSH descriptor: [Self Care] this term only

#82 MeSH descriptor: [Program Evaluation] this term only

#83 MeSH descriptor: [Distance Education] this term only

#84 MeSH descriptor: [Cell Phone] this term only

#85 MeSH descriptor: [Mobile Applications] this term only

#86 ((advice* or advis*)):ti OR ((advice* or advis*)):ab

#87 (coach* or counsel*):ti OR (coach* or counsel*):ab

#88 (stay* near/2 activ*):ti OR (stay* near/2 activ*):ab

#89 (bed* near/2 rest*):ti OR (bed* near/2 rest*):ab

#90 (reassur*):ti OR (reassur*):ab

#91 (((self* near/2 manag*) or self-manag*)):ti OR (((self* near/2 manag*) or self-manag*)):ab

#92 (((self* near/2 care*) or self-care*)):ti OR (((self* near/2 care*) or self-care*)):ab

#93 (((self* near/2 help*) or self-help*)):ti OR (((self* near/2 help*) or self-help*)):ab

#94 (back near/2 school*):ti OR (back near/2 school*):ab

#95 (educat*):ti OR (educat*):ab

#96 (book* or pamphlet* or handout* or leaflet*):ti OR (book* or pamphlet* or handout* or leaflet*):ab

#97 (information* near/2 (online* or written* or oral* or verbal* or digital* or health*)):ti OR (information* near/2 (online* or written* or oral* or verbal* or digital* or health*)):ab

#98 ((website* or web-site* or web-base* or (web* near/2 page*) or (web* near/2 application*) or (web* near/2 interfac*))):ti OR ((website* or web-site* or web-base* or (web* near/2 page*) or (web* near/2 application*) or (web* near/2 interfac*))):ab

#99 (internet*):ti OR (internet*):ab

#100 (((self* near/2 help*) or self-help*)):ti or (((self* near/2 help*) or self-help*)):ab

#101 ((email* or e-mail*)):ti OR ((email* or e-mail*)):ab

#102 ((phone or phones or cellphone* or smartphone or iphone* or i-phone*)):ti OR ((phone or phones or cellphone* or smartphone or iphone* or i-phone*)):ab

#103 ((text* near/2 messag*)):ti OR ((text* near/2 messag*)):ab

#104 ((instruction* near/2 video*)):ti OR ((instruction* near/2 video*)):ab

#105 ((explain* near/2 pain*)):ti OR ((explain* near/2 pain*)):ab

#106 ((normal* near/2 activit*)):ti OR ((normal* near/2 activit*)):ab

#107 (evaluat* near/2 (program* or process*)):ti OR (evaluat* near/2 (program* or process*)):ab

#108 ((day* near/2 activit*)):ti OR ((day* near/2 activit*)):ab

#109 ((daily* near/2 activit*)):ti OR ((daily* near/2 activit*)):ab

#110 ((ordinary* near/2 activit*)):ti OR ((ordinary* near/2 activit*)):ab

#111 (neurophysio*):ti OR (neurophysio*):ab

#112 ((pain* near/2 neuroscience)):ti OR ((pain* near/2 neuroscience)):ab

#113 ((therapeutic* near/2 neuroscience)):ti OR ((therapeutic* near/2 neuroscience)):ab

#114 ((pain* near/2 biolog*)):ti OR ((pain* near/2 biolog*)):ab

#115 ((pain* near/2 science*)):ti OR ((pain* near/2 science*)):ab

#116 (neurobio*):ti OR (neurobio*):ab

#117 (neuroscience*):ti OR (neuroscience*):ab

#118 #1 or #2 or #3 or #4 or #5 or #6 or #7 or #8 or #9 or #10 or #11 or #12 or #13 or #14 or #15 or #16 or #17 or #18 or #19 or #20 or #21 or #22 or #23 or #24 or #25 or #26 or #27 or #28 or #29 or #30 or #31 or #32 or #33 or #34 or #35 or #36 or #37 or #38 or #39 or #40 or #41 or #42 or #43 or #44 or #45 or #46 or #47 or #48 or #49 or #50 or #51 or #52 or #53 or #54 or #55 or #56 or #57 or #58 or #59 or #60 or #61 or #62 or #63 or #64 or #65 or #66

#119 #67 or #68 or #69 or #70 or #71 or #72 or #73 or #74 or #75 or #76 or #77 or #78 or #79 or #80 or #81 or #82 or #83 or #84 or #85 or #86 or #87 or #88 or #89 or #90 or #91 or #92 or #93 or #94 or #95 or #96 or #97 or #98 or #99 or #100 or #101 or #102 or #103 or #104 or #105 or #106 or #107 or #108 or #109 or #110 or #111 or #112 or #113 or #114 or #115 or #116 or #117 or #118

#86 #118 AND #119 with Cochrane Library publication date Between Sep 2020 and Mar 2022

**E. Database:** PEDRO Physiotherapy Evidence Database; https://pedro.org.au/

**Years of search:** 2020 – current

**Date search run:** March 9, 2022

**Number of records retrieved:** 62

**Search Strategy:**

- Abstract & Title: (advice) AND (low back pain)
- [OR] Abstract & Title: ( educat*) AND (low back pain )
- [OR] Abstract & Title: ( counsel*) AND (low back pain)
- [OR] Abstract & Title: ( book*) AND (low back pain)
- [OR] Abstract & Title: ( social media*) AND (low back pain)
- [OR] Abstract & Title: ( website* ) AND (low back pain)

Limit: Published since 2020

Limit: Method = clinical trial

**F. Database:** World Health Organization International Clinical Trials Registry Platform (ICTRP)

https://trialsearch.who.int

**Years of search:** Sept 1, 2020 – March 2022

**Date search run:** March 9, 2022

**Number of records retrieved:** 390

**Search Strategy:**

[Basic search option]

- low back pain AND advice [OR]
- low back pain AND educat* [OR]
- low back and counsel*

Records selected via ‘date of registration’ Sept 1, 2020- current.

**Online Resource 2.** Description of all included randomized controlled trials (RCTs) (n=15 trials; 16 reports)

| **Akca 2017^1^** (Ref. ID 25006) | |
| --- | --- |
| Methods | **Study design:** RCT  **Setting:** Turkey (upper-middle income economy)  **Education/advice groups:** 1  **Comparison groups:** 1 |
| Participants | **Number of participants:** 100 (E1=50, C1=50)  **Mean age, years (SD):** E1=48 (9.5), C1=46 (10.6)  **Gender (female)**^†^**:** E1=76%, C1=75%  **Chronic LBP type:** lumbar disc herniation  **Mean chronic LBP duration, years (SD):** E1=6.1 (5.4), C1=6.5 (4.6)  **Leg pain:** mixed with and without leg pain; majority with pain (radicular) (E1=93%, C1=88%) |
| Interventions | **Intervention:** body mechanics education + physical therapy and medical care (E1)  **Content:** nature of LBP condition, self-management, ergonomic, anatomical/physiological  **Rationale:** multifactorial strategy to improve outcomes and limit recurrence of injury  **Materials:** written brochure (take-home following in-person education)  **Procedures:** 4-week standard physiotherapy and prescription of oral anti-inflammatory agents in case of severe pain. Education sessions provided within the last week of care.  **Format:** group (5-6 subjects per group)  **Duration:** 1 week, 3 sessions, 30 minutes/session  **Location:** physical therapy and rehabilitation unit of hospital  **Provider:** researcher  **Mode of delivery:** verbal (oral presentation with demonstration of correct/incorrect body mechanics), written  **Tailoring:** structured  **Modifications:** individualized (incorrect body mechanics were corrected)  **Adherence:** 90% |
| Comparisons | **Comparisons assessed:** body mechanics education + physical therapy and medical care (E1) vs. physical therapy and medical care alone (C1)  **Intervention:** physical therapy and medical care (C1)  **Procedure:** standard physiotherapy and prescription of oral anti-inflammatory agents in case of severe pain  **Materials:** NR  **Format:** individual  **Duration:** 4 weeks  **Location:** physical therapy and rehabilitation unit of hospital  **Provider:** NR  **Mode of delivery:** in-person  **Tailoring:** NR  **Modifications:** NR |
| Outcomes | Pain (VAS, 0-10)  **Follow-up:** short-term (closest to 3 months) |
| Risk of bias | High (refer to Appendix 2 for details) |
| **Ayanniyi 2015^2^** (Ref. ID 25007) | |
| Methods | **Study design:** RCT  **Setting:** Nigeria (lower-middle income economy)  **Education/advice groups:** 1  **Comparison groups:** 1 |
| Participants | **Number of participants:** 247 (E1=126, C1=121)  **Mean age, years (SD):** range reported 25-60 years  **Gender (female)** ^†^**:** 0%  **Chronic LBP type:** nonspecific (primary)  **Mean chronic LBP duration, years (SD):** NR  **Leg pain:** NR |
| Interventions | **Intervention:** back care education (E1)  **Content:** keep active, exercises, ergonomic, anatomical/physiological  **Rationale:** patient education to support the management of LBP, and reduce the incidence and/or recurrence of injury  **Materials:** education programme, translated into Yoruba language  **Procedures:** education scheduled once per week for the first three weeks and once in two weeks in the next four weeks  **Format:** group  **Duration:** 8 weeks, 5 sessions, 45 minutes/session  **Location:** central location in each village  **Provider:** two principal investigators  **Mode of delivery:** verbal, written  **Tailoring:** structured  **Modifications:** standardized  **Adherence:** NR |
| Comparisons | **Comparisons assessed:** back care education (E1) vs. no treatment (C1)  **Intervention:** no treatment (C1)  **Procedure:** n/a  **Materials:** n/a  **Format:** n/a  **Duration:** n/a  **Location:** n/a  **Provider:** n/a  **Mode of delivery:** n/a  **Tailoring:** n/a  **Modifications:** n/a |
| Outcomes | Pain (Chronic Pain Questionnaire, 0-100), function (Chronic Pain Questionnaire, 0-100)  **Follow-up:** short-term (closest to 3 months) |
| Risk of bias | High (refer to Appendix 2 for details) |
| Notes | Peasant male farmers |
| **Bodes Pardo 2018^3^** (Ref. ID 25000) | |
| Methods | **Study design:** RCT  **Setting:** Spain (high-income economy)  **Education/advice groups:** 1  **Comparison groups:** 1 |
| Participants | **Number of participants:** 56 (E1=28, C1=28)  **Mean age, years (SD):** E1=45 (9.6), C1=49 (10.5)  **Gender (female)** ^†^**:** E1**=**79%, C1=79%  **Chronic LBP type:** nonspecific (primary)  **Mean chronic LBP duration, years (SD):** NR  **Leg pain:** none |
| Interventions | **Intervention:** pain neurophysiology education (E1)  **Content:** pain neuroscience (‘explain pain’) (book *Explain Pain,* available at: [www.paininmotion.be](http://www.paininmotion.be)), exercises  **Rationale:** pain neurophysiology education thought to desensitise the central nervous system when combined with therapeutic exercise; education aims to change patient beliefs and behaviour  **Materials:** written leaflet  **Procedures:** education provided following therapeutic exercise and again one month later  **Format:** group (4-6 per group)  **Duration:** 4 weeks, 2 sessions, 50 min/session  **Location:** NR  **Provider:** physiotherapist  **Mode of delivery:** verbal explanation (with visual presentation), written  **Tailoring:** structured  **Modifications:** standardized  **Adherence:** 100% (1 subject indicated not doing exercises frequently, 4 frequently, 14 very frequently, and 10 always) |
| Comparisons | **Comparisons assessed:** pain neurophysiology education + therapeutic exercise (E1) vs. therapeutic exercise alone (C1)  **Intervention:** therapeutic exercise (C1)  **Procedure:** motor control exercises for lumbar spine, stretching, aerobic exercise  **Materials:** n/a  **Format:** group (4-6 per group)  **Duration:** NR  **Location:** NR  **Provider:** physiotherapist  **Mode of delivery:** in-person  **Tailoring:** physiotherapist corrected each participant individually as required  **Modifications:** NR |
| Outcomes | Pain (NPRS, 0-10), function (RMDQ, 0-24), catastrophizing (PCS Spanish version, 0-52), kinesiophobia (TSK-11 Spanish version, 11-44)  **Follow-up:** short-term (closest to 3 months) |
| Risk of bias | Moderate (refer to Appendix 2 for details) |
| **da Silva 2014^4^** (Ref. ID 25008) | |
| Methods | **Study design:** RCT  **Setting:** Brazil (upper-middle-income economy)  **Education/advice groups:** 1  **Comparison groups:** 1 |
| Participants | **Number of participants:** 20 (E1=10, C1=10)  **Mean age, years (SD):** E1**=**73 (9.6), C1=65 (8.5)  **Gender (female)** ^†^**:** E1**=**100%, C1=89%  **Chronic LBP type:** nonspecific (primary)  **Mean chronic LBP duration, years (SD):** NR  **Leg pain:** NR |
| Interventions | **Intervention:** educational lessons (E1)  **Content:** pain concepts, ergonomic, anatomical/physiological  **Rationale:** education enables patients to become responsible in their own recovery process and in the maintenance of quality of life  **Materials:** written pamphlets  **Procedures:** education lesson followed by written pamphlet to take home encouraging home exercise  **Format:** group  **Duration:** 5 weeks, number of sessions = 10, session duration = 30 minutes  **Location:** NR  **Provider:** physical therapy students and physiotherapists  **Mode of delivery:** verbal, written  **Tailoring:** structured  **Modifications:** standardized  **Adherence:** NR |
| Comparisons | **Comparisons assessed:** educational lessons (E1) vs. waitlist control (C1)  **Intervention:** received take-home pamphlet encouraging home exercise; same pamphlet at E1 (C1)  **Procedure:** n/a  **Materials:** written pamphlets  **Format:** n/a  **Duration:** n/a  **Location:** n/a  **Provider:** n/a  **Mode of delivery:** written  **Tailoring:** n/a  **Modifications:** n/a |
| Outcomes | Pain (VAS, 0-10) function (RMDQ, 0-24), health-related quality of life (WHOQOL-BREF 1-5, 26-130)  **Follow-up:** short-term (closest to 3 months) |
| Risk of bias | High (refer to Appendix 2 for details) |
| Notes | 1. VAS follow-up scores estimated from figure 2 2. RMDQ percent change from baseline estimated from figure 3 3. Baseline SDs carried forward |
| **Ibrahimi Ghavamabadi 2022^5^** (Ref. ID 21997) | |
| Methods | **Study design:** RCT  **Setting:** Iran (lower-middle income economy)  **Education/advice groups:** 1  **Comparison groups:** 1 |
| Participants | **Number of participants:** 250 (E1=125, C1=125)  **Mean age, years (SD):** majority 35-39 (E1=40%, C1=38%)  **Gender (female)** ^†^**:** 0%  **Chronic LBP type:** NR (assumed non-specific (primary))  **Mean chronic LBP duration, years (SD):** NR  **Leg pain:** NR |
| Interventions | **Intervention:** back school training program (E1)  **Content:** nature of condition, lifestyle factors, stress management, exercises, self-management, ergonomic, anatomical/physiological  **Rationale:** education addresses physical and psychological dimensions of participants  **Materials:** written LBP management guide booklet  **Procedures:** weekly 2-hour meetings followed by 15 minute question and answer period  **Format:** group (25 per group)  **Duration:** 4 weeks, number of sessions = 4, session duration = 120 minutes  **Location:** NR  **Provider:** occupational ergonomist  **Mode of delivery:** verbal, written  **Tailoring:** structured  **Modifications:** standardized  **Adherence:** NR |
| Comparisons | **Comparisons assessed:** back school training program (E1) vs. no treatment (C1)  **Intervention:** no treatment (received guide booklet at end of trial) (C1)  **Procedure:** n/a  **Materials:** n/a  **Format:** n/a  **Duration:** n/a  **Location:** n/a  **Provider:** n/a  **Mode of delivery:** n/a  **Tailoring:** n/a  **Modifications:** n/a |
| Outcomes | Pain (VAS, 0-10), function (RMDQ, 0-24), Health-related quality of life (SF-36, 0-100)  **Follow-up:** short-term (closest to 3 months) |
| Risk of bias | High (refer to Appendix 2 for details) |
| Notes | Occupational setting (male industrial workers) |
| **Jassi 2021^6^** (Ref. ID 21838) | |
| Methods | **Study design:** RCT  **Setting:** Brazil (upper-middle-income economy)  **Education/advice groups:** 1  **Comparison groups:** 1 |
| Participants | **Number of participants:** 80 (E1=40, C1=40)  **Mean age, years (SD):** E1= 28 (10.85), C1=28 (11.44)  **Gender (female)** ^†^**:** E1=58%, C1=58%  **Chronic LBP type:** nonspecific (primary)  **Mean chronic LBP duration, years (SD):** NR  **Leg pain:** NR |
| Interventions | **Intervention:** educational booklet for self-management (E1)  **Content:** adapted from the Back Book  **Rationale:** NR  **Materials:** educational booklet  **Procedures:** postural control assessment followed by educational intervention, followed by additional postural control assessment  **Format:** individual  **Duration:** 0 weeks, number of sessions = 1, session duration = 45 minutes  **Location:** outpatient physical therapy clinic  **Provider:** physiotherapist  **Mode of delivery:** verbal (book discussed in an interactive session), written  **Tailoring:** structured  **Modifications:** standardized  **Adherence:** NR |
| Comparisons | **Comparisons assessed:** educational booklet for self-management (E1) vs. sham Kinesio Taping (C1)  **Intervention:** sham Kinesio Taping (C1)  **Procedure:** using elastic adhesive bandage 5.0 cm wide x 0.5 mm thick and water resistant; single bandage placed horizontally, passing through the spinous process of the second lumbar vertebra and applied without any tape tension; participants advised to maintain daily functional activities  **Materials:** elastic adhesive bandage  **Format:** n/a  **Duration:** tape replaced on third day of continuous wear and continued for a period of 7 days  **Location:** outpatient physical therapy clinic  **Provider:** physiotherapist  **Mode of delivery:** in-person  **Tailoring:** n/a  **Modifications:** standardized |
| Outcomes | Pain (NPRS, 0-10), function (ODI, 0-50), fear avoidance (Fear Avoidance Beliefs Questionnaire: Work subscale [FABQ-Work, 0-42], Physical Activities subscale [FABQ-PA, 0-24])  **Follow-up:** short-term (closest to 3 months) |
| Risk of bias | High (refer to Appendix 2 for details) |
| **Kim 2022^7^** (Ref. ID 20270) | |
| Methods | **Study design:** RCT  **Setting:** Korea (high-income economy)  **Education/advice groups:** 1  **Comparison groups:** 1 |
| Participants | **Number of participants:** 40 (E1=20, C1=20)  **Mean age, years (SD):** E1=69 (5.1), C1=71 (5.2)  **Gender (female)** ^†^**:** 100%  **Chronic LBP type:** nonspecific (primary)  **Mean chronic LBP duration, years (SD):** 18-20 months  **Leg pain:** mixed with and without leg pain (non-radicular) |
| Interventions | **Intervention:** pain neuroscience education (E1)  **Content:** pain neuroscience (‘explain pain’)  **Rationale:** aimed at lowering the threat value of pain, increasing the participants’ knowledge of pain, and reconceptualizing pain  **Materials:** ‘presentation materials’  **Procedures:** conducted twice per week for 10 minutes before the start of physical therapy treatments  **Format:** individual  **Duration:** 8 weeks, number of sessions = 16, session duration = 10 minutes  **Location:** NR  **Provider:** physical therapist  **Mode of delivery:** verbal, written  **Tailoring:** structured  **Modifications:** standardized  **Adherence:** 100% (only analyzed those that attended over 80% of treatment sessions) |
| Comparisons | **Comparisons assessed:** pain neuroscience education + lumbar stabilization exercises (E1) vs. lumbar stabilization exercises alone (C1)  **Intervention:** lumbar stabilization exercises (C1)  **Procedure:** gradually progressed, 11 exercises aimed at strengthening hamstring, abdominal and quadriceps stretching, cat-camel, neutral abdominal hollow, curl-up, dead bug, side bridge, superman, bridge, bird dog; 5 repetitions/1 set with 20 second rest time  **Materials:** n/a  **Format:** individual  **Duration:** 2 30-minute sessions per week combined with 20 minutes general physical therapy (hyperthermia, electrotherapy)  **Location:** NR  **Provider:** physical therapist  **Mode of delivery:** in-person  **Tailoring:** NR  **Modifications:** NR |
| Outcomes | Pain (NPRS, 0-10), function (RMDQ, 0-24), fear avoidance (TSK-11, 11-44), catastrophizing (PCS Korean version, 0-52)  **Follow-up:** short-term (closest to 3 months) |
| Risk of bias | High (refer to Appendix 2 for details) |
| **Miyamoto 2021^8^** (Ref. ID 20643) | |
| Methods | **Study design:** RCT  **Setting:** Brazil (upper-middle-income economy)  **Education/advice groups:** 1  **Comparison groups:** 1 |
| Participants | **Number of participants:** 148 (E1=74, C1=74)  **Mean age, years (SD):** E1=47 (14.8), C1=51 (13.2)  **Gender (female)** ^†^**:** E1=51%, C1=58%  **Chronic LBP type:** nonspecific (primary)  **Mean chronic LBP duration, months (SD):** E1=80 (112), C1=91 (93.2)  **Leg pain:** NR |
| Interventions | **Intervention:** education based on LBP guidelines (E1)  **Content:** return to daily activities, self-management (advice on how to cope with pain), nature of condition (explanation of signs and symptoms)  **Rationale:** NR  **Materials:** n/a  **Procedures:** received two 60-minute individual treatment sessions, with one-week interval between them  **Format:** individual  **Duration:** 1 week, 2 sessions, 60 min/session  **Location:** outpatient clinic  **Provider:** physical therapist  **Mode of delivery:** verbal  **Tailoring:** unstructured  **Modifications:** standardized  **Adherence:** 91% (7 participants did not attend second education session (67 attended; 67/74=91%) |
| Comparisons | **Comparisons assessed:** education (E1) vs. no treatment (waitlist) (C1)  **Intervention:** no treatment (waitlist) (C1)  **Procedure:** n/a  **Materials:** n/a  **Format:** n/a  **Duration:** n/a  **Location:** n/a  **Provider:** n/a  **Mode of delivery:** n/a  **Tailoring:** n/a  **Modifications:** n/a |
| Outcomes | Pain (NRS, 0-10) function (ODI, 0-100; PSFS, 0-10)  **Follow-up:** short-term (closest to 3 months), moderate-term (closest to 6 months), long-term (closest to 12 months) |
| Risk of bias | Moderate (refer to Appendix 2 for details) |
| Notes | Function (measured with ODI) is included in meta-analysis. |
| **Morone 2011^9^** (Ref. ID 25002) | |
| Methods | **Study design:** RCT  **Setting:** Italy (high-income economy)  **Education/advice groups:** 1  **Comparison groups:** 1 |
| Participants | **Number of participants:** 73 (E1=44, C1=29)  **Mean age, years (SD):** E1=61 (13.3), C1=59 (12.2)  **Gender (female)** ^†^**:** E1**=**59%, C1=72%  **Chronic LBP type:** nonspecific (primary)  **Mean chronic LBP duration, years (SD):** NR  **Leg pain:** mixed with and without leg pain (non-radicular) |
| Interventions | **Intervention:** back School program (E1)  **Content:** stress management, exercises, ergonomic, pain neuroscience (‘explain pain’), anatomical/physiological, psychological aspects, workplace situations, sport activities, re-education of breathing, self-stretching  **Rationale:** encourage patient active-management for chronic back pain  **Materials:** written pamphlets  **Procedures:** educational sessions followed by a take-home written pamphlet with further explanations of educational material  **Format:** group (4-5 per group)  **Duration:** 4 weeks, number of sessions = 10, session duration = 60 minutes  **Location:** rehabilitation centre  **Provider:** NR  **Mode of delivery:** verbal (patients actively involved), written  **Tailoring:** structured  **Modifications:** standardized  **Adherence:** NR |
| Comparisons | **Comparisons assessed:** back school program + usual care (E1) vs. usual care alone (C1)  **Intervention:** usual care (C1)  **Procedure:** medical/pharmacological assistance for both groups and for the same time period (e.g., analgesics, miorelaxants, NSAIDs when needed and under medical supervision). Physicians were instructed not to start pain therapy during the trial using different drugs (e.g., antidepressants, antiepileptics)  **Materials:** n/a  **Format:** individual  **Duration:** n/a  **Location:** rehabilitation centre  **Provider:** physician  **Mode of delivery:** in-person  **Tailoring:** n/a  **Modifications:** n/a |
| Outcomes | Pain (VAS, 0-10), function (ODI, 0-50; Waddell Disability Index 0-9), health-related quality of life (SF-36, 0-100)  **Follow-up:** short-term (closest to 3 months), moderate term (closest to 6 months) |
| Risk of bias | High (refer to Appendix 2 for details) |
| **Pires 2015^10^** (Ref. ID 25001) | |
| Methods | **Study design:** RCT  **Setting:** Portugal (high-income economy)  **Education/advice groups:** 1  **Comparison groups:** 1 |
| Participants | **Number of participants:** 62 (E1=30, C1=32)  **Mean age, years (SD):** E1=51 (6.2), C1=51(6.3)  **Gender (female)** ^†^**:** E1=67%, C1=63%  **Chronic LBP type:** nonspecific (primary)  **Mean chronic LBP duration, years (SD):** majority >24 months (E1=80%, C1=75%)  **Leg pain:** mixed with and without leg pain (non-radicular) |
| Interventions | **Intervention:** pain neurophysiology education (E1)  **Content:** pain neuroscience (‘explain pain’), exercises  **Rationale:** pain neurophysiology to change maladaptive pain cognitions, illness perceptions or coping strategies, to further introduce normal movement and activity, and reducing pain and functional disability  **Materials:** metaphors and pictures used to challenge participant maladaptive pain cognitions and illness behaviours  **Procedures:** two sessions of education immediately before starting the aquatic exercise programme  **Format:** group  **Duration:** 1 week, number of sessions = 2, session duration = 90 minutes  **Location:** outpatient clinic  **Provider:** physiotherapist  **Mode of delivery:** verbal, pictures  **Tailoring: s**tructured  **Modifications:** standardized  **Adherence:** 100% |
| Comparisons | **Comparisons assessed:** pain neurophysiology education + aquatic exercise (E1) vs. aquatic exercise alone (C1)  **Intervention:** aquatic exercise (C1)  **Procedure:** based on aquatic exercise program for chronic LBP patients  **Materials:** n/a  **Format:** group (6-9 participants)  **Duration:** 12 bi-weekly sessions (30-60 minutes/session)  **Location:** therapeutic pool  **Provider:** physiotherapist  **Mode of delivery:** in-person  **Tailoring:** NR  **Modifications:** NR |
| Outcomes | Pain (VAS, 0-100), function (Quebec Back Pain Disability Scale, 0-100), fear avoidance (TSK, 13-52)  **Follow-up:** short-term (closest to 3 months) |
| Risk of bias | High (refer to Appendix 2 for details) |
| **Rantonen 2018^11^** (Ref. ID 25003) | |
| Methods | **Study design:** RCT  **Setting:** Finland (high-income economy)  **Education/advice groups:** 1  **Comparison groups:** 1 |
| Participants | **Number of participants:** 90 (E1=40, C1=50)  **Mean age, years (SD):** E1=45 (7), C1=46 (7)  **Gender (female)** ^†^**:** E1=32%, C1=40%  **Chronic LBP type:** nonspecific (primary)  **Mean chronic LBP duration, years (SD):** E1=14 (9), C1=11 (9)  **Leg pain:** NR |
| Interventions | **Intervention:** advice (E1)  **Content:** avoid bed rest, reassurance of a positive prognosis, self-management (rapid return to normal activities)  **Rationale:** NR  **Materials:** The Back Book  **Procedures:** participants received back book in the first visit, contents were individually explained by physician  **Format:** individual  **Duration:** 0 weeks, number of sessions = 1, session duration = not reported  **Location:** outpatient clinic  **Provider:** occupational health physician  **Mode of delivery:** verbal, written  **Tailoring:** structured  **Modifications:** standardized  **Adherence:** NR |
| Comparisons | **Comparisons assessed:** advice (E1) vs. no treatment (C1)  **Intervention:** no treatment (C1)  **Procedure:** n/a  **Materials:** n/a  **Format:** n/a  **Duration:** n/a  **Location:** n/a  **Provider:** n/a  **Mode of delivery:** n/a  **Tailoring:** n/a  **Modifications:** n/a |
| Outcomes | Pain (VAS, 0-100), function (RMDQ, physical impairment [PHI] 0-18; ODI), health-related quality of life (15-D quality of life questionnaire score, 0-1)), adverse events (not specified), fear avoidance (FABQ, 13-78; subscales FABQ-work 6-36, FABQ-PA 4-24), work (accumulated number of sickness absence days and periods in 4 years)  **Follow-up:** extra long-term (closest to 2 years) |
| Risk of bias | High (refer to Appendix 2 for details) |
| Notes | 1. Occupational population 2. Adverse events only reported narratively by trial authors 3. Function: RMDQ score used in meta-analysis |
| **Saracoglu 2020^12^** (Ref. ID 22211) | |
| Methods | **Study design:** RCT  **Setting:** Turkey (upper-middle income economy)  **Education/advice groups:** 1  **Comparison groups:** 1 |
| Participants | **Number of participants:** 46 (E1=23, C1=23)  **Mean age, years (SD):** E1=40 (13.7), C1=41 (12.7)  **Gender (female)** ^†^**:** E1=60%, C1=53%  **Chronic LBP type:** nonspecific (primary)  **Mean chronic LBP duration, months (SD):** E1=28 (16.6), C1=35 15.4)  **Leg pain:** NR |
| Interventions | **Intervention:** pain neuroscience education (E1)  **Content:** pain neuroscience (‘explain pain’), stress management, cognitive/behavioural strategies, role of exercise and manual therapy  **Rationale:** the goal of pain neuroscience education is to change the patient’s misconceptions and maladaptive thoughts about pain  **Materials:** slide presentation  **Procedures:** education sessions held once each week prior to manual therapy sessions  **Format:** individual  **Duration:** 4 weeks, number of sessions = 4, session duration = 45 minutes  **Location:** physical therapy hospital  **Provider:** physiotherapist  **Mode of delivery:** verbal, written  **Tailoring:** structured  **Modifications:** standardized  **Adherence:** 87% |
| Comparisons | **Comparisons assessed:** pain neuroscience education + manual therapy + home exercise program (E1) vs. manual therapy + home exercise program alone (C1)  **Intervention:** manual therapy + home exercise (C1)  **Procedure:** manual therapy: individualized according to each patient’s response to treatment (joint mobilizations), home exercise program: aims to increase strength and flexibility of the abdominal, erector spinae, gluteal, quadriceps, and hamstring muscles. The program begins with lumbar and pelvic stretching and warm-up exercises, followed by strengthening exercises. All exercises were explained once and performed under the supervision of the physiotherapist. The participants were then asked to perform all of the exercises with 10 repetitions of each exercise 3 times a day for 4 weeks. None of the participants received feedback regarding the home exercises over the following 4 weeks. At the first follow-up session (at 4 weeks), the physiotherapist confirmed their correct performance of the exercises again.  **Materials:** n/a  **Format:** individual  **Duration:** 8 30-minute sessions, 2 times/week for 4 weeks  **Location:** physical therapy hospital  **Provider:** physiotherapist  **Mode of delivery:** in-person, home  **Tailoring:** manual therapy was individualized according to each patient’s response to treatment  **Modifications:** participants received feedback regarding the home exercises |
| Outcomes | Pain (NPRS, 0-10), function (Back Performance Scale, 0-15; ODI, 0-100), fear avoidance (TSK, 17-68)  **Follow-up:** short-term (closest to 3 months) |
| Risk of bias | High (refer to Appendix 2 for details) |
| Notes | ODI used in meta-analysis |
| **Shojaei 2017^13^** (Ref. ID 22030) | |
| Methods | **Study design:** RCT  **Setting:** Iran (lower-middle income economy)  **Education/advice groups:** 1  **Comparison groups:** 1 |
| Participants | **Number of participants:** 125 (E1=63, C1=62)  **Mean age, years (SD):** majority 30-45 (E1=57%, C1=65%)  **Gender (female)** ^†^**:** E1=18%, C1=24%  **Chronic LBP type:** nonspecific (primary)  **Mean chronic LBP duration, years (SD):** NR  **Leg pain:** mixed with and without leg pain (“sciatica” unknown radicular/non-radicular) |
| Interventions | **Intervention:** educational program (based on social cognitive theory) and ergonomic posture training (E1)  **Content:** stress management, cognitive/behavioural strategies, ergonomic, exercises, self-management  **Rationale:** prevent chronic back pain by understanding the principles of proper body mechanics and learn how to maintain correct ergonomics and vertebral posture during daily activities  **Materials:** NR  **Procedures:** group-based educational lessons followed by question and answer session  **Format:** group  **Duration:** 2 days, number of sessions = 4, session duration = 120 minutes  **Location:** hospital (workplace of participants)  **Provider:** health education specialist  **Mode of delivery:** verbal  **Tailoring:** structured  **Modifications:** standardized  **Adherence:** NR |
| Comparisons | **Comparisons assessed:** Educational program and ergonomic posture training (E1) vs. no treatment (C1)  **Intervention:** no treatment (C1)  **Procedure:** n/a  **Materials:** n/a  **Format:** n/a  **Duration:** n/a  **Location:** n/a  **Provider:** n/a  **Mode of delivery:** n/a  **Tailoring:** n/a  **Modifications:** n/a |
| Outcomes | Pain (VAS, 0-100 [used 0-10]), function (Quebec Back Pain Disability Scale, 0-100), self-efficacy (The behaviour questionnaire, 12-48)  **Follow-up:** moderate term (closest to 6 months) |
| Risk of bias | High (refer to Appendix 2 for details) |
| Notes | 1. Same trial as Shojaei 2017 (25009) (additional report with different outcomes) 2. Self-efficacy outcome from this trial was not reported; instead, reported the self-efficacy outcome from Shojaei 2017 (25009) since it uses the same outcome measurement as the 2-week time point |
| **Shojaei 2017^14^** (Ref. ID 25009) | |
| Methods | **Study design:** RCT  **Setting:** Iran (lower-middle income economy)  **Education/advice groups:** 1  **Comparison groups:** 1 |
| Participants | **Number of participants:** 125 (E1=63, C1=62)  **Mean age, years (SD):** majority 30-45 (E1=57%, C1=65%)  **Gender (female)** ^†^**:** E1=18%, C1=24%  **Chronic LBP type:** nonspecific (primary)  **Mean chronic LBP duration, years (SD):** NR  **Leg pain:** mixed with and without leg pain (“sciatica” unknown radicular/non-radicular) |
| Interventions | **Intervention:** educational program (based on social cognitive theory) and ergonomic posture training (E1)  **Content:** stress management, cognitive/behavioural strategies, ergonomic, exercises, self-management  **Rationale:** prevent chronic back pain by understanding the principles of proper body mechanics and learn how to maintain correct ergonomics and vertebral posture during daily activities  **Materials:** NR  **Procedures:** group-based educational lessons followed by question and answer session  **Format:** group  **Duration:** 2 days, number of sessions = 4, session duration = 120 minutes  **Location:** hospital (workplace of participants)  **Provider:** health education specialist  **Mode of delivery:** verbal  **Tailoring:** structured  **Modifications:** standardized  **Adherence:** NR |
| Comparisons | **Comparisons assessed:** Educational program + ergonomic posture training (E1) vs. no treatment (C1)  **Intervention:** no treatment (C1)  **Procedure:** n/a  **Materials:** n/a  **Format:** n/a  **Duration:** n/a  **Location:** n/a  **Provider:** n/a  **Mode of delivery:** n/a  **Tailoring:** n/a  **Modifications:** n/a |
| Outcomes | Emotional coping (Multidisciplinary Work-related LBP Predictor Questionnaire, Emotional Coping subscale, 4-20), self-efficacy (Multidisciplinary Work-related LBP Predictor Questionnaire, self-efficacy subscale, 7-35)  **Follow-up:** immediate term (closest to 2 weeks), moderate term (closest to 6 months) |
| Risk of bias | High (refer to Appendix 2 for details) |
| Notes | Same trial as Shojaei 2017 (22030) (additional report with different outcomes) |
| **Tellez-Garcia 2015^15^** (Ref. ID 25004) | |
| Methods | **Study design:** RCT  **Setting:** Spain (high-income economy)  **Education/advice groups:** 1  **Comparison groups:** 1 |
| Participants | **Number of participants:** 12 (E1=6, C1=6)  **Mean age, years (SD):** E1=36 (5), C1=37 (13)  **Gender (female)** ^†^**:** 50%  **Chronic LBP type:** nonspecific (primary)  **Mean chronic LBP duration, months (SD):** E1=17 (9), C1=19 (8)  **Leg pain:** none |
| Interventions | **Intervention:** neuroscience education + trigger point dry needling (E1)  **Content:** based on book “Explain Pain”; cognitive/behavioural strategies, pain neuroscience (‘explain pain’), anatomical/physiological  **Rationale:** attempts to increase patients’ understanding of their pain by explaining the underlying neurophysiology of chronic pain in order to modify attitude and, in combination with other therapy interventions, improves pain and disability  **Materials:** PowerPoint presentation, written information  **Procedures:** received dry needling followed by face-to-face individual education session. Written information about pain physiology concepts was discussed and provided in a take-home written format  **Format:** individual  **Duration:** 2 weeks, number of sessions = 2, session duration = 30 minutes  **Location:** NR  **Provider:** experienced clinician (likely a physiotherapy setting)  **Mode of delivery:** verbal, written  **Tailoring:** structured  **Modifications:** standardized, individualized  **Adherence:** NR |
| Comparisons | **Comparisons assessed:** Neuroscience education + trigger point dry needling (E1) vs. trigger point dry needling alone (C1)  **Intervention:** trigger point dry needling (C1)  **Procedure:** active trigger points located in the gluteus medius and quadratus lumborum muscles were treated  **Materials:** n/a  **Format:** individual  **Duration:** 3 sessions  **Location:** NR  **Provider:** experienced clinician (likely a physiotherapy setting)  **Mode of delivery:** in-person  **Tailoring:** none  **Modifications:** NR |
| Outcomes | Pain (NPRS, 0-10), function (RMDQ, 0-24; ODI, 0-50), fear avoidance (TSK, 17-68)  **Follow-up:** short-term (closest to 3 months) |
| Risk of bias | High (refer to Appendix 2 for details) |
| Notes | RMDQ scores used in meta-analysis |
| **Zhang 2014^16^** (Ref. ID 25005) | |
| Methods | **Study design:** RCT  **Setting:** China (upper-middle income economy)  **Education/advice groups:** 1  **Comparison groups:** 1 |
| Participants | **Number of participants:** 54 (E1=27, C1=27)  **Mean age, years (SD):** E1=22.3 (2.9), C1=23.0 (2.2)  **Gender (female)** ^†^**:** 33%  **Chronic LBP type:** NR (assumed nonspecific (primary))  **Mean chronic LBP duration, months (SD):** E1=10.9 (3.7), C1=11.5 (3.5)  **Leg pain:** NR |
| Interventions | **Intervention:** education + exercise (E1)  **Content:** keep active, self-management, ergonomic  **Rationale:** postural hygiene used to train patient how to perform daily activities in such a way that back muscle tension and spinal load are reduced  **Materials:** videos, computer presentations, instructional leaflets  **Procedures:** Sessions included a lecture (30 min) followed by discussion (10 min)  **Format:** individual  **Duration:** 12 weeks, number of sessions = 12, session duration = 40 minutes  **Location:** NR  **Provider:** physical therapist  **Mode of delivery:** verbal, written, electronic  **Tailoring:** structured  **Modifications:** standardized  **Adherence:** NR |
| Comparisons | **Comparisons assessed:** education + exercise (E1) vs. exercise alone (C1)  **Intervention:** lumbar strengthening exercises (C1)  **Procedure:** 5-min warm-up; 15-min trunk flexor strength exercises, including straight leg raises and sit-ups with foot fixation; 15-min trunk extensor strength exercises, including prone trunk extensions; 5-min cool-down  **Materials:** n/a  **Format:** individual  **Duration:** 40-minute sessions, 3 times/week for 12 weeks  **Location:** NR  **Provider:** physical therapist  **Mode of delivery:** in-person  **Tailoring:** NR  **Modifications:** NR |
| Outcomes | Pain (VAS, 0-10), function (ODI, 0-50), health-related quality of life (SF-36, 0-100)  **Follow-up:** short-term (closest to 3 months) |
| Risk of bias | High (refer to Appendix 2 for details) |

^†^We used the terms ‘female or male’ to describe gender because these were the terms used by trial authors; however, we recognize that gender is a social construct and sex is a biological construct

**C1:** Comparison treatment group 1, **E1:** Education or advice treatment group 1, **FABQ-PA:** Fear Avoidance Beliefs Questionnaire Physical Activities subscale, **FABQ-W:** Fear Avoidance Beliefs Questionnaire Work subscale, **LBP:** low back pain, **NPRS:** numeric pain rating scale, **n/a:** not applicable; **NR:** not reported; **ODI:** Oswestry Disability Index, PCS: Pain Catastrophizing Scale, PHI: Physical impairment, QofL: Quality of life, **RCT:** randomized controlled trial, **RMDQ:** Roland-Morris Disability Questionnaire, **SD:** standard deviation, **SF-36:** 36-item Short Form Health Survey, **TSK:** Tampa Scale of Kinesiophobia, **VAS:** Visual Analogue Scale, **WHOQOL-BREF:** World Health Organization Quality of Life abbreviated questionnaire, **WI:** Waddell Disability Index

**Online Resource 3.** List of excluded trials with reasons for exclusion (n=75)

| Reason | Trial |
| --- | --- |
| Ineligible publication type  n = 4 | 1. Antunes M, Bertolini S. Comparison between the intervention with back school and postural reeducation in the posture of elderly people with low back pain (abstract). 2021.^17^ 2. Ferreira GE, Lin CC, Stevens ML, Hancock MJ, Latimer J, Wisbey-Roth T, Maher CG. TOPS – a randomized controlled trial of exercise and education to prevent recurrence of low back pain: statistical analysis plan. 2020.^18^ 3. Gul H, Erel S. Effect of therapeutic neuroscience education combined with physical therapy in individuals with chronic low back pain (abstract). 2019.^19^ 4. Shakil-ur-Rehman (PI) Effects of Patient Education Manual in Patient With Chronic Low Back Pain. Identifier:  NCT04600843.^20^ |
| Ineligible study design  n = 5 | 1. Attanayake AM, P. Somarathna KIWK, Vyas GH, Dash SC. Clinical evaluation of selected Yogic procedures in individuals with low back pain. 2010.^21^ 2. Ghadyani L, Tavafian SS, Kazemnejad A, Wagner J. Effectiveness of Multidisciplinary Group-Based Intervention versus Individual Physiotherapy for Improving Chronic Low Back Pain in Nursing Staff: A Clinical Trial with 3- and 6-Month Follow-Up Visits from Tehran, Iran. 2017.^22^ 3. Gul H, Erel S, Toraman NF. Physiotherapy combined with therapeutic neuroscience education versus physiotherapy alone for patients with chronic low back pain: a pilot, randomized-controlled trial. 2021.^23^ 4. Saxena P, Thiyagarajan S. Scapular and Pelvic PNF Pattern for Female Physical Education Students with Low Back Pain. 2020.^24^ 5. Sharafkhani N, Khorsandi M, Shamsi M, Ranjbaran M. The Effect of an Educational Intervention Program on the Adoption of Low Back Pain Preventive Behaviors in Nurses: An Application of the Health Belief Model. 2016. ^25^ |
| Ineligible population  n = 38 | 1. Aghilinejad M, Bahrami-Ahmadi A, Kabir-Mokamelkhah E, Sarebanha S, Hosseini HR, Sadeghi, Z. The effect of three ergonomics training programs on the prevalence of low-back pain among workers of an Iranian automobile factory: a randomized clinical trial. 2014.^26^ 2. Anan T, Kajiki, S, Oka H, Fujii T, Kawamata K, Mori K, Matsudaira K. Effects of an Artificial Intelligence-Assisted Health Program on Workers With Neck/Shoulder Pain/Stiffness and Low Back Pain: randomized Controlled Trial. 2021.^27^ 3. Baumeister H, Paganini S, Sander LB, Lin J, Schlicker S, Terhorst Y, Moshagen M, Bengel J, Lehr D, Ebert DD. Effectiveness of a Guided Internet- and Mobile-Based Intervention for Patients with Chronic Back Pain and Depression (WARD-BP): A Multicenter, Pragmatic Randomized Controlled Trial. 2021.^28^ 4. Buhrman M, Faltenhag S, Strom L, Andersson G. Controlled trial of Internet-based treatment with telephone support for chronic back pain. 2004.^29^ 5. Corsinovi L, Martinelli E, Fonte G, Astengo M, Sona A, Gatti A, Massaia M, Bo M, Zanocchi M, Michelis G, Isaia G, Molaschi M. Efficacy of oxycodone/acetaminophen and codeine/acetaminophen vs. conventional therapy in elderly women with persistent, moderate to severe osteoarthritis-related pain. 2009.^30^ 6. Dagenais S, Hayflinger DC, Mayer JM. Economic evaluation of an extended telehealth worksite exercise intervention to reduce lost work time from low back pain in career firefighters. 2021.^31^ 7. Delitto A, Patterson CG, Stevans JM, Freburger JK, Khoja SS, Schneider MJ, Greco CM, Freel JA, Sowa GA, Wasan AD, Brennan GP, Hunter SJ, Minick KI, Wegener ST, Ephraim PL, Beneciuk JM, George SZ, Saper RB. Stratified care to prevent chronic low back pain in high-risk patients: the TARGET trial. A multi-site pragmatic cluster randomized trial [with consumer summary]. 2021.^32^ 8. Dwyer CP, MacNeela P, Durand H, O'Connor LL, Main CJ, McKenna-Plumley PE, Hamm RM, Reynolds B, Conneely S, Slattery BW, et al. Effects of Biopsychosocial Education on the Clinical Judgments of Medical Students and GP Trainees Regarding Future Risk of Disability in Chronic Lower Back Pain: a Randomized Control Trial. 2020.^33^ 9. Foster NE, Konstantinou K, Lewis M, Ogollah R, Saunders B, Kigozi J, Jowett S, Bartlam B, Artus M, Hill JC, et al. Stratified versus usual care for the management of primary care patients with sciatica: the SCOPiC RCT. 2020.^34^ 10. Fu WJ, Ying LH. Application of nursing intervention guided by king’s interactive standard theory in patients with lumbar disc herniation undergoing conservative treatment. 2021.^35^ 11. Galan-Martin MA, Montero-Cuadrado F, Lluch-Girbes E, Coca-Lopez MC, Mayo-Iscar A, Cuesta-Vargas A. Pain neuroscience education and physical therapeutic exercise for patients with chronic spinal pain in spanish physiotherapy primary care: A pragmatic randomized controlled trial. 2020.^36^ 12. George SZ, Teyhen DS, Wu SS, Wright AC, Dugan JL, Yang G, Robinson ME, Childs JD. Psychosocial education improves low back pain beliefs: results from a cluster randomized clinical trial (NCT00373009) in a primary prevention setting. 2009.^37^ 13. Hodges PW, Hall L, Setchell J, French S, Kasza J, Bennell K, Hunter D, Vicenzino B, Crofts S, Dickson C, et al. Effect of a Consumer-Focused Website for Low Back Pain on Health Literacy, Treatment Choices, and Clinical Outcomes: randomized Controlled Trial. 2021.^38^ 14. Johnsen TL, Eriksen HR, Baste V, Indahl A, Odeen M, Tveito TH. Effect of Reassuring Information About Musculoskeletal and Mental Health Complaints at the Workplace: a Cluster Randomized Trial of the at Work Intervention. 2019.^39^ 15. Katri Maria T, Laura AM, Erja P, Timo R, Sirkka K, Marja-Liisa K, Sarianna S, Riku N. Effects of a home-based rehabilitation program in community-dwelling older people after discharge from hospital: a subgroup analysis of a randomized controlled trial. 2021.^40^ 16. Kazemi S-S, Tavafian S-S, Hiller CE, Hidarnia A, Montazeri A. The effectiveness of social media and in-person interventions for low back pain conditions in nursing personnel (SMILE). 2021.^41^ 17. Konstantinou K, Lewis M, Dunn K, Ogollah R, Artus M, Hill J, Hughes G, Robinson M, Saunders B, Bartlam B, Kigozi J, Jowett S, Mallen C, Hay E, van der Windt DA, Foster NE. Stratified care versus usual care for management of patients presenting with sciatica in primary care (SCOPiC): a randomised controlled trial [with consumer summary]. 2020^42^ 18. Lane E, Magel JS, Thackeray A, Greene T, Fino NF, Puentedura EJ, Louw A, Maddox D, Fritz JM. Effectiveness of training physical therapists in pain neuroscience education for patients with chronic spine pain: cluster-randomized trial. 2022.^43^ 19. Langagergaard V, Jensen OK, Nielsen CV, Jensen C, Labriola M, Sørensen VN, Pedersen P. The comparative effects of brief or multidisciplinary intervention on return to work at 1 year in employees on sick leave due to low back pain: a randomized controlled trial. 2021.^44^ 20. Lee JA, Ha I-H, Choi T-Y, Choi J, Jun JH, Kang B-K, Lee MS. Evaluating the clinical application of a leaflet for clinical practice guideline in patients with lumbar herniated intervertebral discs: Randomized controlled trial. 2017.^45^ 21. McCurry SM, Zhu W, Von Korff M, Wellman R, Morin CM, Thakral M, Yeung K, Vitiello MV. Effect of Telephone Cognitive Behavioral Therapy for Insomnia in Older Adults With Osteoarthritis Pain: a Randomized Clinical Trial. 2021.^46^ 22. Meisel ZF, Shofer F, Dolan A, Goldberg EB, Rhodes KV, Hess EP, Bellamkonda VR, Perrone J, Cannuscio CC, Becker L, Rodgers MA, Zyla MM, Bell JJ, McCollum S, Engel-Rebitzer E, Tiako MJN, Ridgeway G, Schapira MM. A Multicentered Randomized Controlled Trial Comparing the Effectiveness of Pain Treatment Communication Tools in Emergency Department Patients With Back or Kidney Stone Pain. 2022.^47^ 23. Moore JE, Von Korff M, Cherkin D, Saunders K, Lorig K. A randomized trial of a cognitive-behavioral program for enhancing back pain self care in a primary care setting. 2000.^48^ 24. Pereira OS. Efficacy of the Santhiflex method of psychomotor postural re-education in the treatment of chronic low back pain. 2015.^49^ 25. Pourhaji F, Delshad MH, Tavafian SS, Niknami S, Pourhaji F. Effects of educational program based on precede-proceed model in promoting low back pain behaviors (EPPLBP) in health care workers Shahid Beheshti University of Medical Sciences: randomized trial. 2020.^50^ 26. Sandal LF, Bach K, Overas CK, Svendsen MJ, Dalager T, Stejnicher Drongstrup Jensen J, Kongsvold A, Nordstoga AL, Bardal EM, Ashikhmin I, Wood K, Rasmussen CDN, Stochkendahl MJ, Nicholl BI, Wiratunga N, Cooper K, Hartvigsen J, Kjaer P, Sjogaard G, Nilsen TIL, Mair FS, Sogaard K, Mork PJ. Effectiveness of App-Delivered, Tailored Self-management Support for Adults With Lower Back Pain-Related Disability: A selfBACK Randomized Clinical Trial. 2021.^51^ 27. Schroder K, Oberg B, Enthoven P, Hedevik H, Abbott A. Improved adherence to clinical guidelines for low back pain after implementation of the BetterBack model of care: A stepped cluster randomized controlled trial within a hybrid type 2 trial. 2022.^52^ 28. Sharma S, Traeger AC, O'Keeffe M, Copp T, Freeman A, Hoffmann T, Maher CG. Effect of information format on intentions and beliefs regarding diagnostic imaging for non-specific low back pain: a randomised controlled trial in members of the public. 2021.^53^ 29. Shebib R, Bailey JF, Smittenaar P, Perez DA, Mecklenburg G, Hunter S. Randomized controlled trial of a 12-week digital care program in improving low back pain. 2019.^54^ 30. Simula AS, Jenkins HJ, Hancock MJ, Malmivaara A, Booth N, Karppinen J. Patient education booklet to support evidence-based low back pain care in primary care - a cluster randomized controlled trial. 2021.^55^ 31. Slater H, Briggs AM, Watkins K, Chua J, Smith AJ. Translating evidence for low back pain management into a consumer-focussed resource for use in community pharmacies: a cluster-randomised controlled trial. 2013.^56^ 32. Stuart Donaldson C, Stanger LM, Donaldson MW, Cram J, Skubick DL. A randomized crossover investigation of a back pain and disability prevention program: Possible mechanisms of change. 1993.^57^ 33. Suman A, Schaafsma FG, van Dongen JM, Elders PJM, Buchbinder R, van Tulder MW, Anema JR. Effectiveness and cost-utility of a multifaceted eHealth strategy to improve back pain beliefs of patients with non-specific low back pain: a cluster randomised trial. 2019.^58^ 34. Rhon DI, Mayhew RJ, Greenlee TA, Fritz JM. The influence of a MOBile-based video Instruction for Low back pain (MOBIL) on initial care decisions made by primary care providers: a randomized controlled trial. 2021.^59^ 35. Riva S, Camerini A-L, Allam A, Schulz PJ. Interactive sections of an Internet-based intervention increase empowerment of chronic back pain patients: randomized controlled trial. 2014.^60^ 36. Rosenberg NR, Petersen SB, Begtrup LM, Flachs EM, Petersen JA, Hansen BB, Kirkeskov L, Bliddal H, Christensen R, Kristensen LE, Fournier GL, Kryger AI. Early Occupational Intervention for People with Low Back Pain in Physically Demanding Jobs: 1-year Follow-up Results of the Randomized Controlled GOBACK Trial. 2021.^61^ 37. Ryynänen K, Oura P, Simula AS, Holopainen R, Paukkunen M, Lausmaa M, Remes J, Booth N, Malmivaara A, Karppinen J. Effectiveness of training in guideline-oriented biopsychosocial management of low-back pain in occupational health services - a cluster randomized controlled trial. 2021.^62^ 38. Zarifsanaiey N, Yazdani Z, Karimian Z, Raeisi Shahraki H, Ghaffarinejad F. The Effect of Interactive Multimedia Versus Illustrated Booklet on Behavior to Prevent Lower Back Pain in Working Nurses. 2021.^63^ |
| Ineligible intervention  n = 12 | 1. Burns SA, Cleland JA, Rivett DA, O'Hara MC, Egan W, Pandya J, Snodgrass SJ. When Treating Coexisting Low Back Pain and Hip Impairments, Focus on the Back: adding Specific Hip Treatment Does Not Yield Additional Benefits-A Randomized Controlled Trial. 2021.^64^ 2. Carpenter KM, Stoner SA, Mundt JM, Stoelb B. An online self-help CBT intervention for chronic lower back pain. 2012.^65^ 3. Garcia LM, Birckhead BJ, Krishnamurthy P, Sackman J, Mackey IG, Louis RG, Salmasi V, Maddox T, Darnall BD. An 8-Week Self-Administered At-Home Behavioral Skills-Based Virtual Reality Program for Chronic Low Back Pain: Double-Blind, Randomized, Placebo-Controlled Trial Conducted During COVID-19. 2021.^66^ 4. Garaud T, Gervais C, Szekely B, Michel-Cherqui M, Dreyfus J-E, Fischler M. Randomized study of the impact of a therapeutic education program on patients suffering from chronic low-back pain who are treated with transcutaneous electrical nerve stimulation. 2018.^67^ 5. Narciso Garcia A, da Cunha Menezes Costa L, Mota da Silva T, Lopes Barreto Gondo F, Navarro Cyrillo F, Alqualo Costa R, Oliveira Pena Costa L. Effectiveness of Back School Versus McKenzie Exercises in Patients With Chronic Nonspecific Low Back Pain: A Randomized Controlled Trial. 2013.^68^ 6. Jinnouchi H, Matsudaira K, Kitamura A, Kakihana H, Oka H, Hayama-Terada M, Yamagishi K, Kiyama M, Iso H, CIRCS Investigators. Effects of brief self-exercise education on the management of chronic low back pain: A community-based, randomized, parallel-group pragmatic trial. 2021.^69^ 7. Maggi L, Celletti C, Mazzarini M, Blow D, Camerota F. Neuromuscular taping for chronic non-specific low back pain: a randomized single-blind controlled trial. 2022.^70^ 8. de Moraes EB, Martins Junior FF, da Silva LB, Garcia JBS, de Mattos-Pimenta CA. Self-efficacy and fear of pain to movement in chronic low back pain: an intervention developed by nurses. 2021.^71^ 9. Rabiei P, Sheikhi B, Letafatkar A. Comparing pain neuroscience education followed by motor control exercises with group-based exercises for chronic low back pain: a randomized controlled trial. 2021.^72^ 10. Shimo K, Hasegawa M, Mizutani S, Hasegawa T, Ushida T. Effects of a 12-week workplace counseling program on physical activity and low back pain: a pilot randomized controlled study. 2021.^73^ 11. Walsh N, Jones L, Phillips S, Thomas R, Odondi L, Palmer S, Cramp F, Pollock J, Hurley M. Facilitating Activity and Self-management for people with Arthritic knee, hip or lower back pain (FASA): a cluster randomised controlled trial. 2020.^74^ 12. Williams A, Wiggers J, O'Brien KM, Wolfenden K, Yoong SL, Hodder RL, Lee H, Robson EK, McAuley JH, Haskins R, Kamper SJ, Rissel C, Williams CM. Effectiveness of a healthy lifestyle intervention for chronic low back pain: a randomised controlled trial. 2018.^75^ |
| Ineligible comparison  n = 16 | 1. Amaral DDV, Miyamoto GC, Franco KFM, Dos Santos Franco YR, Bastos De Oliveira NT, Hancock MJ, Van Tulder MW, Nunes Cabral CM. Examination of a Subgroup of Patients With Chronic Low Back Pain Likely to Benefit More Lrom Pilates-Based Exercises Compared to an Educational Booklet. 2020.^76^ 2. Berlowitz J, Hall D, Joyce C, Fredman L, Sherman K, Saper R, Roseen E. Changes in perceived stress after yoga, physical therapy, and education interventions for chronic low back pain: A secondary analysis of a randomized controlled trial. 2020.^77^ 3. Caldas VVDA, Maciel DG, Cerqueira MS, Barboza JAM, Neto JBV, Dantas G, de Melo RRV, de Souza RNS, Vieira WHDB. Effect of Pain Education, Cryotherapy, and Transcutaneous Electrical Nerve Stimulation on the Pain, Functional Capacity, and Quality of Life in Patients With Nonspecific Chronic Low Back Pain: A Single-Blind Randomized Controlled Trial. 2021.^78^ 4. Chiauzzi E, Pujol LA, Wood M, Bond K, Black R, Yiu E, Zacharoff K. painACTION-back pain: a self-management website for people with chronic back pain. 2010.^79^ 5. Darnall BD, Roy A, Chen AL, Ziadni MS, Keane RT, You DS, Slater K, Poupore-King H, MacKey I, Kao M-C, et al. Comparison of a Single-Session Pain Management Skills Intervention with a Single-Session Health Education Intervention and 8 Sessions of Cognitive Behavioral Therapy in Adults with Chronic Low Back Pain: a Randomized Clinical Trial. 2021.^80^ 6. Ghadyani L, Tavafian SS, Kazemnejad A, Wagner J. Work-Related Low Back Pain Treatment: A Randomized Controlled Trial from Tehran, Iran, Comparing Multidisciplinary Educational Program versus Physiotherapy Education. 2016.^81^ 7. Ibrahim MI, Zubair IU, Shafei MN, Ahmad MI, Yaacob NM. Interactive Low Back Pain Intervention Module Based on the Back School Program: A Cluster-Randomized Experimental Study Evaluating Its Effectiveness among Nurses in Public Hospitals. 2020.^82^ 8. Kim S-Y, Kim H-S, Chung S-S. Effects of an Individualized Educational Program for Korean Patients With Chronic Low Back Pain: A Randomized Controlled Trial. 2021.^83^ 9. Kohns DJ, Urbanik CP, Geisser ME, Schubiner H, Lumley MA. The Effects of a Pain Psychology and Neuroscience Self-Evaluation Internet Intervention: A Randomized Controlled Trial. 2020.^84^ 10. Marshall A, Joyce CT, Tseng B, Gerlovin H, Yeh GY, Sherman KJ, Saper RB, Roseen EJ. Changes in pain self-efficacy, coping skills and fear avoidance beliefs in a randomized controlled trial of yoga, physical therapy, and education for chronic low back pain. 2022.^85^ 11. Mendes Tozim B, Thomaz de Aquino Nava G, Zuliani Stroppa Marques AE, Tavella Navega, M. Efficacy of the Pilates versus general exercises versus educational workshops on neuromuscular parameters: A randomized controlled trial. 2021.^86^ 12. Orhan C, Lenoir D, Favoreel A, van Looveren E, Yildiz Kabak V, Mukhtar NB, Cagnie B, Meeus M. Culture-sensitive and standard pain neuroscience education improves pain, disability, and pain cognitions in first-generation Turkish migrants with chronic low back pain: a pilot randomized controlled trial. 2021.^87^ 13. Rios JCS, Hua FY, Safons MP. Posture-focused self-management programme improves pain and function in older people with chronic low back pain: a randomised controlled trial. 2020.^88^ 14. Unal M, Evcik E, Kocaturk M, Algun ZC. Investigating the effects of myofascial induction therapy techniques on pain, function and quality of life in patients with chronic low back pain. 2020.^89^ 15. Yang C-Y, Tsai Y-A, Wu P-K, Ho S-Y, Chou C-Y, Huang S-F. Pilates-based core exercise improves health-related quality of life in people living with chronic low back pain: a pilot study. 2021.^90^ 16. Yildirim P, Gultekin A. The Effect of a Stretch and Strength-Based Yoga Exercise Program on Patients with Neuropathic Pain due to Lumbar Disc Herniation. 2022.^91^ |

**Online Resource 4.** Risk of bias assessment for the included trials (n=15) (16 reports)

| Trial | Random allocation | Concealed allocation | Participant blinding | Provider blinding | Assessor blinding | Drop-outs | Intention-to-treat analysis | Selective reporting | Similar groups (baseline) | Co-interventions | Compliance | Timing of outcomes | Other biases | Overall ROB Rating |
| --- | --- | --- | --- | --- | --- | --- | --- | --- | --- | --- | --- | --- | --- | --- |
|  | Selection bias | | Performance bias | | Detection bias | Attrition bias | | Reporting bias | Selection bias | Other biases | | | |  |
| Akca 2017 | 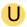 | 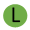 | 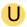 | 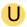 | 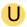 | 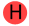 | 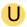 | 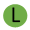 | 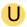 | 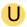 | 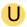 | 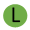 | 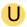 | High |
| Ayanniyi 2015 | 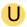 | 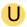 | 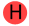 | 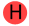 | 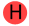 | 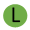 | 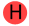 | 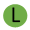 | 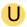 | 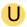 | 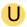 | 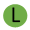 | 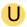 | High |
| Bodes Pardo 2018 | 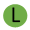 | 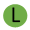 | 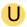 | 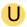 | 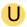 | 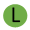 | 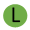 | 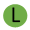 | 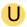 | 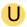 | 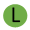 | 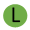 | 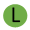 | Unclear |
| DaSilva 2014 | 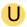 | 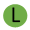 | 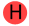 | 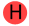 | 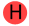 | 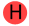 | 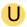 | 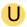 | 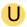 | 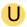 | 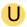 | 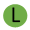 | 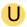 | High |
| Ibrahami 2021 | 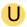 | 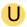 | 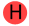 | 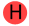 | 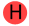 | 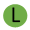 | 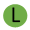 | 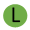 | 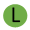 | 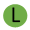 | 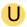 | 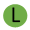 | 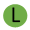 | High |
| Jassi 2021 | 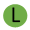 | 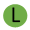 | 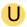 | 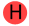 | 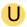 | 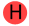 | 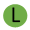 | 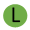 | 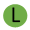 | 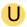 | 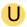 | 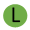 | 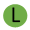 | High |
| Kim 2022 | 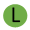 | 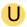 | 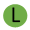 | 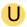 | 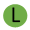 | 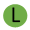 | 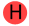 | 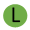 | 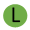 | 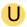 | 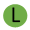 | 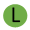 | 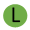 | High |
| Miyamoto 2021 | 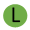 | 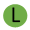 | 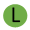 | 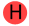 | 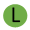 | 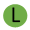 | 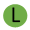 | 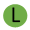 | 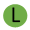 |  |  |  |  | Unclear |
| Morone 2011 |  |  |  |  |  |  |  |  |  |  |  |  |  | High |
| Pires 2015 |  |  |  |  |  |  |  |  |  |  |  |  |  | High |
| Rantonen 2018 |  |  |  |  |  |  |  |  |  |  |  |  |  | High |
| Saracoglu 2020 |  |  |  |  |  |  |  |  |  |  |  |  |  | High |
| Shojaei 2017 (22030) |  |  |  |  |  |  |  |  |  |  |  |  |  | High |
| Shojaei 2017 (25009) |  |  |  |  |  |  |  |  |  |  |  |  |  | High |
| Tellez-Garcia 2015 |  |  |  |  |  |  |  |  |  |  |  |  |  | High |
| Zhang 2014 |  |  |  |  |  |  |  |  |  |  |  |  |  | High |

Risk of bias assessment conducted using ROB1 criteria. H=high risk of bias, L=low risk of bias, ROB=risk of bias, U=Unclear

Risk of bias graph: review authors’ judgements about each risk of bias item presented as percentages across all included trials

**Online Resource 5.** GRADE evidence profile tables

GRADE evidence profile 1: ***What are the benefits and harms of education/advice in the management of community-dwelling adults (including older adults aged 60 years and over) with chronic primary low back pain (with or without leg pain) compared to no intervention or interventions where the effect of education/advice could be isolated?***

| **Certainty assessment** | | | | | | | **№ of patients** | | **Effect** | | **Certainty** | **Importance** |
| --- | --- | --- | --- | --- | --- | --- | --- | --- | --- | --- | --- | --- |
| **№ of studies** | **Study design** | **Risk of bias** | **Inconsistency** | **Indirectness** | **Imprecision** | **Other considerations** | **Education or advice** | **No treatment** | **Relative (95% CI)** | **Absolute (95% CI)** |  |  |
| **ALL ADULTS** | | | | | | | | | | | | |
| **Pain (follow-up: closest to 3 months; assessed with: NRS, VAS, Chronic Pain Questionnaire; benefit indicated by lower values; Scale from: 0 to 10)** | | | | | | | | | | | | |
| 10^1,2,3,4,5,6,7,8,9,10^ | randomised trials | very serious^a^ | serious^b^ | not serious^c^ | serious^d^ | none | 430 | 428 | - | MD **1.1 lower** (1.63 lower to 0.56 lower) | ⨁◯◯◯ Very low | CRITICAL |
| **Pain in males (follow-up: closest to 3 months; assessed with: VAS, Chronic Pain Questionnaire; benefit indicated by lower values; Scale from: 0 to 10)** | | | | | | | | | | | | |
| 2^1,4^ | randomised trials | very serious^a^ | not serious^e^ | not serious^f^ | serious | none | 225 | 225 | - | MD **1.12 lower** (1.5 lower to 0.74 lower) | ⨁◯◯◯ Very low | CRITICAL |
| **Pain in females and males (follow-up: closest to 3 months; assessed with: NRS; benefit indicated by lower values; Scale from: 0 to 10)** | | | | | | | | | | | | |
| 7^2,3,6,7,8,9,10^ | randomised trials | very serious^a^ | serious^g^ | not serious^c^ | serious^h^ | none | 187 | 186 | - | MD **1.16 lower** (2.08 lower to 0.23 lower) | ⨁◯◯◯ Very low | CRITICAL |
| **Pain in females (follow-up: closest to 3 months; assessed with: NRS, VAS; benefit indicated by lower values; Scale from: 0 to 10)** | | | | | | | | | | | | |
| 1^5^ | randomised trials | very serious^a^ | not serious^i^ | serious^j^ | very serious^k^ | none | 18 | 17 | - | MD **0.69 lower** (1.56 lower to 0.18 higher) | ⨁◯◯◯ Very low | CRITICAL |
|  | | | | | | | | | | | | |
|  | | | | | | | | | | | | |
| **Pain in people with unclassified presence of leg pain (follow-up: closest to 3 months; assessed with: NRS, VAS, Chronic Pain Questionnaire; benefit indicated by lower values; Scale from: 0 to 10)** | | | | | | | | | | | | |
| 6^1,3,4,6,8,10^ | randomised trials | very serious^a^ | serious^l^ | not serious^c^ | serious^d^ | none | 349 | 351 | - | MD **1.01 lower** (1.85 lower to 0.17 lower) | ⨁◯◯◯ Very low | CRITICAL |
| **Pain in people without leg pain (follow-up: closest to 3 months; assessed with: NRS; benefit indicated by lower values; Scale from: 0 to 10)** | | | | | | | | | | | | |
| 2^2,9^ | randomised trials | very serious^a^ | serious^m^ | serious^n^ | very serious^k^ | none | 34 | 34 | - | MD **1.33 lower** (12.08 lower to 9.42 higher) | ⨁◯◯◯ Very low | CRITICAL |
| **Pain in people either with or without non-radicular leg pain (follow-up: closest to 3 months; assessed with: NRS, VAS; benefit indicated by lower values; Scale from: 0 to 10)** | | | | | | | | | | | | |
| 2^5,7^ | randomised trials | very serious^a^ | serious^b^ | not serious^o^ | very serious^k^ | none | 49 | 43 | - | MD **1.15 lower** (7.99 lower to 5.69 higher) | ⨁◯◯◯ Very low | CRITICAL |
| **Pain in trials undertaken in low to lower-middle income countries (follow-up: closest to 3 months; assessed with: VAS, Chronic Pain Questionnaire; benefit indicated by lower values; Scale from: 0 to 10)** | | | | | | | | | | | | |
| 2^1,4^ | randomised trials | very serious^a^ | not serious^e^ | not serious^f^ | serious^d^ | none | 225 | 225 | - | MD **1.12 lower** (1.5 lower to 0.74 lower) | ⨁◯◯◯ Very low | CRITICAL |
| **Pain in trials undertaken in high to upper-middle income countries (follow-up: closest to 3 months; assessed with: NRS, VAS, benefit indicated by lower values; Scale from: 0 to 10)** | | | | | | | | | | | | |
| 8^2,3,5,6,7,8,9,10^ | randomised trials | very serious^a^ | serious^p^ | not serious^c^ | serious^d^ | none | 205 | 203 | - | MD **1.09 lower** (1.86 lower to 0.31 lower) | ⨁◯◯◯ Very low | CRITICAL |
| **Pain stratified by race/ethnicity** | | | | | | | | | | | | |
| 0 |  |  |  |  |  |  |  |  |  |  |  | CRITICAL |
|  | | | | | | | | | | | | |
| **Pain (education intervention: mixed content) (follow-up: closest to 3 months; assessed with: NRS, VAS, Chronic Pain Questionnaire; benefit indicated by lower values; Scale from: 0 to 10)** | | | | | | | | | | | | |
| 5^1,3,4,6,10^ | randomised trials | very serious^a^ | not serious^q^ | not serious^c^ | serious^r^ | none | 329 | 332 | - | MD **0.8 lower** (1.41 lower to 0.19 lower) | ⨁◯◯◯ Very low | CRITICAL |
| **Pain (education intervention: pain neuroscience) (follow-up: closest to 3 months; assessed with: NRS, VAS; benefit indicated by lower values; Scale from: 0 to 10)** | | | | | | | | | | | | |
| 5^2,5,7,8,9^ | randomised trials | very serious^a^ | serious^p^ | not serious^o^ | serious^h^ | none | 101 | 96 | - | MD **1.47 lower** (2.57 lower to 0.37 lower) | ⨁◯◯◯ Very low | CRITICAL |
| **Pain (education intervention delivery mode: combined verbal and written and/or electronic) (follow-up: closest to 3 months; assessed with: NRS, VAS, Chronic Pain Questionnaire; benefit indicated by lower values; Scale from: 0 to 10)** | | | | | | | | | | | | |
| 7^1,2,4,5,8,9,10^ | randomised trials | very serious^a^ | serious^s^ | not serious^c^ | serious^t^ | none | 322 | 319 | - | MD **1.21 lower** (1.84 lower to 0.57 lower) | ⨁◯◯◯ Very low | CRITICAL |
| **Pain (education intervention delivery mode: verbal) (follow-up: closest to 3 months; assessed with: NRS, VAS; benefit indicated by lower values; Scale from: 0 to 10)** | | | | | | | | | | | | |
| 3^3,6,7^ | randomised trials | very serious^a^ | serious^u^ | not serious^c^ | very serious^v^ | none | 108 | 109 | - | MD **0.68 lower** (3.19 lower to 1.83 higher) | ⨁◯◯◯ Very low | CRITICAL |
| **Pain (after removing high risk of bias studies) (follow-up: closest to 3 months; assessed with: NRS; benefit indicated by lower values; Scale from: 0 to 10)** | | | | | | | | | | | | |
| 2^2,6^ | randomised trials | very serious^a^ | very serious^w^ | not serious^c^ | very serious^x^ | none | 102 | 102 | - | MD **1.1 lower** (13.41 lower to 11.22 higher) | ⨁◯◯◯ Very low | CRITICAL |
|  | | | | | | | | | | | | |
|  | | | | | | | | | | | | |
|  | | | | | | | | | | | | |
| **Pain (follow-up: closest to 6 months; assessed with: NRS; benefit indicated by lower values; Scale from: 0 to 10)** | | | | | | | | | | | | |
| 1^6^ | randomised trials | very serious^a^ | not serious^i^ | serious^j^ | very serious^k^ | none | 74 | 74 | - | MD **0.55 lower** (1.49 lower to 0.39 higher) | ⨁◯◯◯ Very low | CRITICAL |
| **Pain (follow-up: closest to 12 months; assessed with: NRS; benefit indicated by lower values; Scale from: 0 to 10)** | | | | | | | | | | | | |
| 1^6^ | randomised trials | very serious^a^ | not serious^i^ | serious^j^ | very serious^k^ | none | 74 | 74 | - | MD **1.35 lower** (2.34 lower to 0.36 lower) | ⨁◯◯◯ Very low | CRITICAL |
| **Pain (follow-up: 2 years; assessed with: VAS; benefit indicated by lower values; Scale from: 0 to 100)** | | | | | | | | | | | | |
| 1^11^ | randomised trials | very serious^a^ | not serious^i^ | serious^j^ | very serious^k^ | none | 40 | 50 | - | MD **8 lower** (18.14 lower to 2.14 higher) | ⨁◯◯◯ Very low | CRITICAL |
| **Function (follow-up: closest to 3 months; assessed with: RMDQ, ODI, Chronic Pain Questionnaire, Quebec Back Pain Disability Scale; benefit indicated by lower values)** | | | | | | | | | | | | |
| 10^1,2,3,4,5,6,7,8,9,10^ | randomised trials | very serious^a^ | serious^p^ | not serious^c^ | serious^d^ | none | 430 | 428 | - | SMD **0.51 lower** (0.89 lower to 0.12 lower) | ⨁◯◯◯ Very low | CRITICAL |
| **Function in males (follow-up: closest to 3 months; assessed with: RMDQ, Chronic Pain Questionnaire; benefit indicated by lower values)** | | | | | | | | | | | | |
| 2^1,4^ | randomised trials | very serious^a^ | not serious^e^ | not serious^f^ | serious^y^ | none | 225 | 225 | - | SMD **0.4 lower** (0.79 lower to 0 ) | ⨁◯◯◯ Very low | CRITICAL |
| **Function in females and males (follow-up: closest to 3 months; assessed with: RMDQ, ODI, Chronic Pain Questionnaire, Quebec Back Pain Disability Scale; benefit indicated by lower values)** | | | | | | | | | | | | |
| 7^2,3,6,7,8,9,10^ | randomised trials | very serious^a^ | serious^z^ | not serious^o^ | serious^aa^ | none | 187 | 186 | - | SMD **0.55 lower** (1.22 lower to 0.13 higher) | ⨁◯◯◯ Very low | CRITICAL |
| **Function in females (follow-up: closest to 3 months; assessed with: RMDQ; benefit indicated by lower values)** | | | | | | | | | | | | |
| 1^5^ | randomised trials | very serious^a^ | not serious^i^ | serious^j^ | very serious^k^ | none | 18 | 17 | - | SMD **0.58 lower** (1.26 lower to 0.1 higher) | ⨁◯◯◯ Very low | CRITICAL |
| **Function in people with unclassified presence of leg pain (follow-up: closest to 3 months; assessed with: RMDQ, ODI, Chronic Pain Questionnaire; benefit indicated by lower values)** | | | | | | | | | | | | |
| 6^1,3,4,6,8,10^ | randomised trials | very serious^a^ | not serious^ab^ | not serious^c^ | serious^d^ | none | 349 | 351 | - | SMD **0.35 lower** (0.62 lower to 0.07 lower) | ⨁◯◯◯ Very low | CRITICAL |
| **Function in people without leg pain (follow-up: closest to 3 months; assessed with: RMDQ; benefit indicated by lower values)** | | | | | | | | | | | | |
| 2^2,9^ | randomised trials | very serious^a^ | not serious^e^ | serious^n^ | very serious^k^ | none | 34 | 34 | - | SMD **1.46 lower** (3.33 lower to 0.41 higher) | ⨁◯◯◯ Very low | CRITICAL |
| **Function in people either with or without non-radicular leg pain (follow-up: closest to 3 months; assessed with: RMDQ, Chronic Pain Questionnaire; benefit indicated by lower values)** | | | | | | | | | | | | |
| 2^5,7^ | randomised trials | very serious^a^ | not serious^e^ | not serious^o^ | very serious^k^ | none | 47 | 43 | - | SMD **0.49 lower** (1.41 lower to 0.43 higher) | ⨁◯◯◯ Very low | CRITICAL |
| **Function in trials undertaken in low to lower-middle income countries (follow-up: closest to 3 months; assessed with: RMDQ, Chronic Pain Questionnaire; benefit indicated by lower values)** | | | | | | | | | | | | |
| 2^1,4^ | randomised trials | very serious^a^ | not serious^q^ | not serious^f^ | serious^y^ | none | 225 | 225 | - | SMD **0.4 lower** (0.79 lower to 0 ) | ⨁◯◯◯ Very low | CRITICAL |
| **Function in trials undertaken in high to upper-middle income countries (follow-up: closest to 3 months; assessed with: RMDQ, ODI, Quebec Back Pain Disability Scale; benefit indicated by lower values)** | | | | | | | | | | | | |
| 8^2,3,5,6,7,8,9,10^ | randomised trials | very serious^a^ | serious^ac^ | not serious^o^ | serious^ad^ | none | 205 | 203 | - | SMD **0.55 lower** (1.1 lower to 0 ) | ⨁◯◯◯ Very low | CRITICAL |
|  | | | | | | | | | | | | |
| **Function stratified by race/ethnicity** | | | | | | | | | | | | |
| 0 |  |  |  |  |  |  |  |  |  |  |  | CRITICAL |
| **Function (education intervention: mixed content) (follow-up: closest to 3 months; assessed with: RMDQ, ODI, Chronic Pain Questionnaire; benefit indicated by lower values)** | | | | | | | | | | | | |
| 5^1,3,4,6,10^ | randomised trials | very serious^a^ | not serious^ae^ | not serious^c^ | serious^y^ | none | 329 | 332 | - | SMD **0.28 lower** (0.68 lower to 0.11 higher) | ⨁◯◯◯ Very low | CRITICAL |
| **Function (education intervention: pain neuroscience) (follow-up: closest to 3 months; assessed with: RMDQ, ODI, Quebec Back Pain Disability Scale; benefit indicated by lower values)** | | | | | | | | | | | | |
| 5^2,5,7,8,9^ | randomised trials | very serious^a^ | not serious^af^ | not serious^o^ | serious^ag^ | none | 101 | 96 | - | SMD **0.87 lower** (1.46 lower to 0.28 lower) | ⨁◯◯◯ Very low | CRITICAL |
| **Function (education intervention delivery mode: combined verbal, written, and/or electronic) (follow-up: closest to 3 months; assessed with: RMDQ, ODI, Chronic Pain Questionnaire; benefit indicated by lower values)** | | | | | | | | | | | | |
| 7^1,2,4,5,8,9,10^ | randomised trials | very serious^a^ | serious^ah^ | not serious^c^ | serious^ai^ | none | 322 | 319 | - | SMD **0.68 lower** (1.08 lower to 0.28 lower) | ⨁◯◯◯ Very low | CRITICAL |
| **Function (education intervention delivery mode: verbal) (follow-up: closest to 3 months; assessed with: RMDQ, ODI, Quebec Back Pain Disability Scale; benefit indicated by lower values)** | | | | | | | | | | | | |
| 3^3,6,7^ | randomised trials | very serious^a^ | serious^aj^ | not serious^o^ | very serious^v^ | none | 108 | 109 | - | SMD **0.08 lower** (1.52 lower to 1.36 higher) | ⨁◯◯◯ Very low | CRITICAL |
| **Function (after removing high risk of bias studies) (follow-up: closest to 3 months; assessed with: RMDQ, ODI; benefit indicated by lower values)** | | | | | | | | | | | | |
| 2^2,6^ | randomised trials | very serious^a^ | very serious^w^ | not serious^o^ | very serious^x^ | none | 102 | 102 | - | SMD **0.74 lower** (9.46 lower to 7.98 higher) | ⨁◯◯◯ Very low | CRITICAL |
|  | | | | | | | | | | | | |
|  | | | | | | | | | | | | |
| **Function (follow-up: closest to 6 months; assessed with: ODI; benefit indicated by lower values; Scale from: 0 to 100)** | | | | | | | | | | | | |
| 1^6^ | randomised trials | very serious^a^ | not serious^i^ | serious^j^ | very serious^k^ | none | 74 | 74 | - | MD **2.86 lower** (7.51 lower to 1.79 higher) | ⨁◯◯◯ Very low | CRITICAL |
| **Function (follow-up: closest to 12 months; assessed with: ODI; benefit indicated by lower values; Scale from: 0 to 100)** | | | | | | | | | | | | |
| 1^6^ | randomised trials | very serious^a^ | not serious^i^ | serious^j^ | very serious^k^ | none | 74 | 74 | - | MD **4.66 lower** (9.68 lower to 0.36 higher) | ⨁◯◯◯ Very low | CRITICAL |
| **Function (follow-up: 2 years; assessed with: RMDQ; benefit indicated by lower values; Scale from: 0 to 24)** | | | | | | | | | | | | |
| 1^11^ | randomised trials | very serious^a^ | not serious^i^ | serious^j^ | very serious^k^ | none | 40 | 50 | - | MD **1.5 lower** (3.42 lower to 0.42 higher) | ⨁◯◯◯ Very low | CRITICAL |
| **Health-related quality of life (unclassified presence of leg pain) (follow-up: closest to 3 months; assessed with: SF-36 (PCS); benefit indicated by higher values; Scale from: 0 to 100)** | | | | | | | | | | | | |
| 2^4,10^ | randomised trials | very serious^a^ | not serious^e^ | not serious^c^ | serious^ak^ | none | 150 | 149 | - | MD **24.27 higher** (12.93 higher to 35.61 higher) | ⨁◯◯◯ Very low | CRITICAL |
| **Health-related quality of life (unclassified presence of leg pain) (follow-up: closest to 3 months; assessed with: SF-36 (MCS); benefit indicated by higher values; Scale from: 0 to 100)** | | | | | | | | | | | | |
| 2^4,10^ | randomised trials | very serious^a^ | very serious^al^ | not serious^c^ | very serious^x^ | none | 125 | 125 | - | MD **13.99 higher** (62.04 lower to 90.03 higher) | ⨁◯◯◯ Very low | CRITICAL |
|  | | | | | | | | | | | | |
|  | | | | | | | | | | | | |
|  | | | | | | | | | | | | |
| **Health-related quality of life (follow-up: closest to 3 months; assessed with: WHOQOL-BREF; benefit indicated by higher values; Scale from: 26 to 130)** | | | | | | | | | | | | |
| 1^3^ | randomised trials | very serious^a^ | not serious^i^ | serious^j^ | very serious^k^ | none | 8 | 9 | - | MD **9.4 lower** (17 lower to 1.8 lower) | ⨁◯◯◯ Very low | CRITICAL |
| **Fear avoidance (high-income country) (follow-up: closest to 3 months; assessed with: TSK, TSK-11; benefit indicated by lower values)** | | | | | | | | | | | | |
| 5^2,5,7,8,9^ | randomised trials | very serious^a^ | serious^am^ | not serious^o^ | serious^ag^ | none | 72 | 70 | - | SMD **1.4 lower** (2.51 lower to 0.29 lower) | ⨁◯◯◯ Very low | CRITICAL |
| **Fear avoidance in females and males (follow-up: closest to 3 months; assessed with: TSK, TSK-11; benefit indicated by lower values)** | | | | | | | | | | | | |
| 4^2,7,8,9^ | randomised trials | very serious^a^ | serious^an^ | not serious^o^ | serious^aa^ | none | 83 | 79 | - | SMD **1.57 lower** (3.21 lower to 0.07 higher) | ⨁◯◯◯ Very low | CRITICAL |
| **Fear avoidance in females (follow-up: closest to 3 months; assessed with: TSK-11; benefit indicated by lower values; Scale from: 11 to 44)** | | | | | | | | | | | | |
| 1^5^ | randomised trials | very serious^a^ | not serious^i^ | serious^j^ | very serious^k^ | none | 18 | 17 | - | MD **7.59 lower** (12.63 lower to 2.55 lower) | ⨁◯◯◯ Very low | CRITICAL |
| **Fear avoidance in people without leg pain (follow-up: closest to 3 months; assessed with: TSK, TSK-11; benefit indicated by lower values)** | | | | | | | | | | | | |
| 2^2,9^ | randomised trials | very serious^a^ | not serious^ao^ | not serious^o^ | very serious^k^ | none | 34 | 34 | - | SMD **2.12 lower** (7.61 lower to 3.37 higher) | ⨁◯◯◯ Very low | CRITICAL |
| **Fear avoidance in people either with or without non-radicular leg pain (follow-up: closest to 3 months; assessed with: TSK, TSK-11; benefit indicated by lower values)** | | | | | | | | | | | | |
| 2^5,7^ | randomised trials | very serious^a^ | not serious^ap^ | not serious^o^ | very serious^k^ | none | 47 | 43 | - | SMD **0.67 lower** (3.89 lower to 2.55 higher) | ⨁◯◯◯ Very low | CRITICAL |
| **Fear avoidance in people with unclassified presence of leg pain (follow-up: closest to 3 months; assessed with: TSK; benefit indicated by lower values)** | | | | | | | | | | | | |
| 1^8^ | randomised trials | very serious^a^ | not serious^i^ | serious^j^ | very serious^k^ | none | 20 | 19 | - | SMD **1.52 lower** (2.24 lower to 0.8 lower) | ⨁◯◯◯ Very low | CRITICAL |
| **Fear avoidance (after removing high risk of bias studies) (follow-up: closest to 3 months; assessed with: TSK, TSK-11; benefit indicated by lower values)** | | | | | | | | | | | | |
| 1^2^ | randomised trials | very serious^a^ | not serious^i^ | serious^j^ | very serious^k^ | none | 28 | 28 | - | SMD **1.95 lower** (2.59 lower to 1.31 lower) | ⨁◯◯◯ Very low | CRITICAL |
| **Fear avoidance in trials undertaken in low to lower-middle income countries** | | | | | | | | | | | | |
| 0 |  |  |  |  |  |  |  |  |  |  |  | CRITICAL |
| **Fear avoidance stratified by race/ethnicity** | | | | | | | | | | | | |
| 0 |  |  |  |  |  |  |  |  |  |  |  | CRITICAL |
| **Fear avoidance (follow-up: 2 years; assessed with: FABQ; benefit indicated by lower values; Scale from: 13 to 78)** | | | | | | | | | | | | |
| 1^11^ | randomised trials | very serious^a^ | not serious^i^ | serious^j^ | very serious^k^ | none | 40 | 50 | - | MD **1 lower** (7.13 lower to 5.13 higher) | ⨁◯◯◯ Very low | CRITICAL |
| **Catastrophizing (follow-up: closest to 3 months; assessed with: Pain Catastrophizing Scale; benefit indicated by lower values; Scale from: 0 to 52)** | | | | | | | | | | | | |
| 2^2,5^ | randomised trials | very serious^a^ | serious^aq^ | not serious^o^ | very serious^k^ | none | 46 | 45 | - | MD **10.19 lower** (55.46 lower to 35.07 higher) | ⨁◯◯◯ Very low | CRITICAL |
|  | | | | | | | | | | | | |
|  | | | | | | | | | | | | |
|  | | | | | | | | | | | | |
|  | | | | | | | | | | | | |
| **Catastrophizing (females and males, no leg pain) (follow-up: closest to 3 months; assessed with: Pain Catastrophizing Scale; benefit indicated by lower values; Scale from: 0 to 52)** | | | | | | | | | | | | |
| 1^2^ | randomised trials | very serious^a^ | not serious^i^ | serious^j^ | very serious^k^ | none | 28 | 28 | - | MD **13.9 lower** (17.16 lower to 10.64 lower) | ⨁◯◯◯ Very low | CRITICAL |
| **Catastrophizing (females, either with or without non-radicular leg pain) (follow-up: closest to 3 months; assessed with: Pain Catastrophizing Scale; benefit indicated by lower values; Scale from: 0 to 52)** | | | | | | | | | | | | |
| 1^5^ | randomised trials | very serious^a^ | not serious^i^ | serious^j^ | very serious^k^ | none | 18 | 17 | - | MD **6.77 lower** (8.48 lower to 5.06 lower) | ⨁◯◯◯ Very low | CRITICAL |
| **Catastrophizing in trials undertaken in high to upper-middle income countries (follow-up: closest to 3 months; assessed with: Pain Catastrophizing Scale; benefit indicated by lower values; Scale from: 0 to 52)** | | | | | | | | | | | | |
| 2^2,5^ | randomised trials | very serious^a^ | serious^aq^ | not serious^o^ | very serious^k^ | none | 46 | 45 | - | MD **10.19 lower** (55.46 lower to 35.07 higher) | ⨁◯◯◯ Very low | CRITICAL |
| **Catastrophizing in trials undertaken in low to lower-middle income countries** | | | | | | | | | | | | |
| 0 |  |  |  |  |  |  |  |  |  |  |  | CRITICAL |
| **Catastrophizing stratified by race/ethnicity** | | | | | | | | | | | | |
| 0 |  |  |  |  |  |  |  |  |  |  |  | CRITICAL |
| **Catastrophizing (after removing high risk of bias studies) (follow-up: closest to 3 months; assessed with: Pain Catastrophizing Scale; benefit indicated by lower values; Scale from: 0 to 52)** | | | | | | | | | | | | |
| 1^2^ | randomised trials | very serious^a^ | not serious^i^ | serious^j^ | very serious^k^ | none | 28 | 28 | - | MD **13.9 lower** (17.16 lower to 10.64 lower) | ⨁◯◯◯ Very low | CRITICAL |
|  | | | | | | | | | | | | |
|  | | | | | | | | | | | | |
|  | | | | | | | | | | | | |
| **Depression (females and males, low-income country, either with or without leg pain unclassified) (follow-up: closest to 2 weeks; assessed with: Multidisciplinary Work-related LBP Predictor Questionnaire, Emotional Coping subscale; benefit indicated by higher values; Scale from: 4 to 20)** | | | | | | | | | | | | |
| 1^12^ | randomised trials | very serious^a^ | not serious^i^ | serious^j^ | serious^ag^ | none | 63 | 62 | - | MD **2.1 higher** (1.05 higher to 3.15 higher) | ⨁◯◯◯ Very low | CRITICAL |
| **Depression (females and males, low-income country, either with or without leg pain unclassified) (follow-up: closest to 6 months; assessed with: Multidisciplinary Work-related LBP Predictor Questionnaire, Emotional Coping subscale; benefit indicated by higher values; Scale from: 4 to 20)** | | | | | | | | | | | | |
| 1^12^ | randomised trials | very serious^a^ | not serious^i^ | serious^j^ | serious^ag^ | none | 63 | 62 | - | MD **1.5 higher** (0.5 higher to 2.5 higher) | ⨁◯◯◯ Very low | CRITICAL |
| **Depression stratified by gender** | | | | | | | | | | | | |
| 0 |  |  |  |  |  |  |  |  |  |  |  | CRITICAL |
| **Depression in trials undertaken in high to upper-middle income countries** | | | | | | | | | | | | |
| 0 |  |  |  |  |  |  |  |  |  |  |  | CRITICAL |
| **Depression stratified by race/ethnicity** | | | | | | | | | | | | |
| 0 |  |  |  |  |  |  |  |  |  |  |  | CRITICAL |
| **Anxiety** | | | | | | | | | | | | |
| 0 |  |  |  |  |  |  |  |  |  |  |  | CRITICAL |
| **Self-efficacy (females and males, low-income country, either with or without leg pain unclassified) (follow-up: closest to 2 weeks; assessed with: Multidisciplinary Work-related LBP Predictor Questionnaire, Self-efficacy subscale; benefit indicated by higher values; Scale from: 7 to 35)** | | | | | | | | | | | | |
| 1^12^ | randomised trials | very serious^a^ | not serious^i^ | serious^j^ | serious^ag^ | none | 63 | 62 | - | MD **4.4 higher** (2.77 higher to 6.03 higher) | ⨁◯◯◯ Very low | CRITICAL |
|  | | | | | | | | | | | | |
|  | | | | | | | | | | | | |
| **Self-efficacy (females and males, low-income country, either with or without leg pain unclassified) (follow-up: closest to 6 months; assessed with: Multidisciplinary Work-related LBP Predictor Questionnaire, Self-efficacy subscale; benefit indicated by higher values; Scale from: 7 to 35)** | | | | | | | | | | | | |
| 1^12,ar^ | randomised trials | very serious^a^ | not serious^i^ | serious^j^ | serious^ag^ | none | 63 | 62 | - | MD **1.6 higher** (0.04 higher to 3.16 higher) | ⨁◯◯◯ Very low | CRITICAL |
| **Self-efficacy stratified by gender** | | | | | | | | | | | | |
| 0 |  |  |  |  |  |  |  |  |  |  |  | CRITICAL |
| **Self-efficacy in trials undertaken in high to upper-middle income countries** | | | | | | | | | | | | |
| 0 |  |  |  |  |  |  |  |  |  |  |  | CRITICAL |
| **Self-efficacy stratified by race/ethnicity** | | | | | | | | | | | | |
| 0 |  |  |  |  |  |  |  |  |  |  |  | CRITICAL |
| **Social participation (paid work) (females and males, high-income country, unclassified presence of leg pain) (follow-up: 2 years; assessed with: number of sickness absence days; benefit indicated by lower values)** | | | | | | | | | | | | |
| 1^11^ | randomised trials | very serious^a^ | not serious^i^ | serious^j^ | very serious^k^ | none | 40 | 50 | - | MD **11 lower** (44 lower to 22 higher) | ⨁◯◯◯ Very low | CRITICAL |
| **Social participation (paid work) stratified by gender (follow-up: 2 years; assessed with: number of sickness absence days)** | | | | | | | | | | | | |
| 0 |  |  |  |  |  |  |  |  |  |  |  | CRITICAL |
| **Social participation (paid work) in trials undertaken in low to lower-middle income countries** | | | | | | | | | | | | |
| 0 |  |  |  |  |  |  |  |  |  |  |  | CRITICAL |
| **Social participation (paid work) stratified by race/ethnicity** | | | | | | | | | | | | |
| 0 |  |  |  |  |  |  |  |  |  |  |  | CRITICAL |
| **Change in use of medications** | | | | | | | | | | | | |
| 0 |  |  |  |  |  |  |  |  |  |  |  | CRITICAL |
| **Health literacy** | | | | | | | | | | | | |
| 0 |  |  |  |  |  |  |  |  |  |  |  | CRITICAL |
| **Adverse events/harms (people with uncertain presence of leg pain, high-income country) (follow-up: 2 years)** | | | | | | | | | | | | |
| 1^11^ | randomised trials | very serious^a^ | not serious^i^ | serious^j^ | serious^ag^ | none | The trial author reported that no adverse events were reported by participants (n=90) during the interventions. | | | | ⨁◯◯◯ Very low | CRITICAL |
| **OLDER ADULTS (aged 60 years or more)** | | | | | | | | | | | | |
| **Pain (high-income country) (follow-up: closest to 3 months; assessed with: NRS, VAS; benefit indicated by lower values; Scale from: 0 to 10)** | | | | | | | | | | | | |
| 2^3,5^ | randomised trials | very serious^a^ | not serious^e^ | not serious^o^ | very serious^k^ | none | 23 | 26 | - | MD **0.5 lower** (5.42 lower to 4.41 higher) | ⨁◯◯◯ Very low | CRITICAL |
| **Pain (females, either with or without non-radicular leg pain) (follow-up: closest to 3 months; assessed with: NRS; benefit indicated by lower values; Scale from: 0 to 10)** | | | | | | | | | | | | |
| 1^5^ | randomised trials | very serious^a^ | not serious^i^ | serious^j^ | very serious^k^ | none | 18 | 17 | - | MD **0.69 lower** (1.56 lower to 0.18 higher) | ⨁◯◯◯ Very low | CRITICAL |
| **Pain (females and males, unclassified presence of leg pain) (follow-up: closest to 3 months; assessed with: VAS; benefit indicated by lower values; Scale from: 0 to 10)** | | | | | | | | | | | | |
| 1^3^ | randomised trials | very serious^a^ | not serious^i^ | serious^j^ | very serious^k^ | none | 5 | 9 | - | **0.3 higher** (2.38 lower to 2.98 higher) | ⨁◯◯◯ Very low | CRITICAL |
| **Pain in trials undertaken in low to lower-middle income countries** | | | | | | | | | | | | |
| 0 |  |  |  |  |  |  |  |  |  |  |  | CRITICAL |
| **Pain stratified by race/ethnicity** | | | | | | | | | | | | |
| 0 |  |  |  |  |  |  |  |  |  |  |  | CRITICAL |
| **Function (high-income country) (follow-up: closest to 3 months; assessed with: RMDQ; benefit indicated by lower values)** | | | | | | | | | | | | |
| 2^3,5^ | randomised trials | very serious^a^ | very serious^as^ | not serious^c^ | very serious^k^ | none | 23 | 26 | - | SMD **0.02 lower** (9.79 lower to 9.76 higher) | ⨁◯◯◯ Very low | CRITICAL |
|  | | | | | | | | | | | | |
| **Function (females, either with or without non-radicular leg pain) (follow-up: closest to 3 months; assessed with: RMDQ; benefit indicated by lower values; Scale from: 0 to 24)** | | | | | | | | | | | | |
| 1^5^ | randomised trials | very serious^a^ | not serious^i^ | serious^j^ | very serious^k^ | none | 18 | 17 | - | MD **1.12 lower** (2.37 lower to 0.13 higher) | ⨁◯◯◯ Very low | CRITICAL |
| **Function (females and males, unclassified presence of leg pain) (follow-up: closest to 3 months; assessed with: RMDQ; benefit indicated by lower values; Scale from: 0 to 24)** | | | | | | | | | | | | |
| 1^3^ | randomised trials | very serious^a^ | not serious^i^ | serious^j^ | very serious^k^ | none | 5 | 9 | - | MD **4.52 higher** (0.46 higher to 8.58 higher) | ⨁◯◯◯ Very low | CRITICAL |
| **Function in trials undertaken in low to lower-middle income countries** | | | | | | | | | | | | |
| 0 |  |  |  |  |  |  |  |  |  |  |  | CRITICAL |
| **Function stratified by race/ethnicity** | | | | | | | | | | | | |
| 0 |  |  |  |  |  |  |  |  |  |  |  | CRITICAL |
| **Fear avoidance (females, high-income country, either with or without non-radicular leg pain) (follow-up: closest to 3 months; assessed with: TSK-11; benefit indicated by lower values)** | | | | | | | | | | | | |
| 1^5^ | randomised trials | very serious^a^ | not serious^i^ | serious^j^ | very serious^k^ | none | 18 | 17 | - | SMD **0.97 lower** (1.68 lower to 0.27 lower) | ⨁◯◯◯ Very low | CRITICAL |
| **Fear-avoidance in males** | | | | | | | | | | | | |
| 0 |  |  |  |  |  |  |  |  |  |  |  | CRITICAL |
| **Fear avoidance in trials undertaken in low to lower-middle income countries** | | | | | | | | | | | | |
| 0 |  |  |  |  |  |  |  |  |  |  |  | CRITICAL |
| **Fear avoidance stratified by race/ethnicity** | | | | | | | | | | | | |
| 0 |  |  |  |  |  |  |  |  |  |  |  | CRITICAL |
| **Health-related quality of life** | | | | | | | | | | | | |
| 0 |  |  |  |  |  |  |  |  |  |  |  | CRITICAL |
| **Depression** | | | | | | | | | | | | |
| 0 |  |  |  |  |  |  |  |  |  |  |  | CRITICAL |
| **Catastrophizing** | | | | | | | | | | | | |
| 0 |  |  |  |  |  |  |  |  |  |  |  | CRITICAL |
| **Anxiety** | | | | | | | | | | | | |
| 0 |  |  |  |  |  |  |  |  |  |  |  | CRITICAL |
| **Self-efficacy** | | | | | | | | | | | | |
| 0 |  |  |  |  |  |  |  |  |  |  |  | CRITICAL |
| **Change in use of medications** | | | | | | | | | | | | |
| 0 |  |  |  |  |  |  |  |  |  |  |  | CRITICAL |
| **Falls** | | | | | | | | | | | | |
| 0 |  |  |  |  |  |  |  |  |  |  |  | CRITICAL |
| **Adverse events/harms** | | | | | | | | | | | | |
| 0 |  |  |  |  |  |  |  |  |  |  |  | CRITICAL |

**CI:** confidence interval; **FABQ:** Fear Avoidance Beliefs Questionnaire; **LBP**: low back pain; **MCS:** mental component summary; **MD:** mean difference; **n/a:** non-applicable; **NRS:** numerical rating scale; **ODI:** Oswestry Disability Index; **OIS:** Optimal Information Size; **PCS:** Physical Component Summary; **RMDQ:** Rolland Morris Disability Questionnaire; **SF-36:** short form health survey; **SMD:** standardised mean difference; **TSK:**Tampa Scale of Kinesiophopia; **VAS:** Visual Analogue Scale; **WHOQOL-BREF:**World Health Organization Quality of Life Questionnaire – Brief version

The following was used to guide the ratings.

**Risk of bias:** *Not serious:* all or most of the weight (>50%) comes from overall low risk of bias trial(s). *Serious:* some of the weight (<50%) comes from overall low risk of bias trial(s). *Very serious:* all or most of the weight (>50%) comes from overall high or unclear risk of bias trial(s).

**Inconsistency:** *Not serious:* high extent of similarity of point estimates and overlap of confidence intervals; statistical heterogeneity (I^2^) is between 0% and 40%, which might not be important. *Serious:* some extent of similarity of point estimates and overlap of confidence intervals; statistical heterogeneity (I^2^) is between 30% and 60%, which could not be explained due to small subgroups and may represent moderate heterogeneity. *Very serious:* little or no similarity of point estimates and overlap of confidence intervals; statistical heterogeneity (I^2^) is between 50% and 90% or 75% and 100%, which could not be explained due to small subgroups and may represent substantial or considerable heterogeneity, respectively.

**Indirectness:** *Not serious:* trial(s) were conducted in different countries or settings. *Serious:* trial(s) were conducted from a single country/setting. *Very serious:* evidence is not directly related to PICO question.

**Imprecision:** *Not serious:* Optimal Information Size (OIS) was reached (i.e., sample sizes with at least 200 participants per group may provide prognostic balance); and the entire confidence interval lies on one side of the threshold that may be considered clinically important (≥10% scale range or SMD ≥0.2 for continuous variables, ≥10% for binary variables), such that the clinical course of action would not differ if the upper versus the lower boundary of the confidence interval represented the truth. *Serious:* OIS would not have been reached (sample sizes with less than 200 participants per group); if the OIS was reached, the clinical course of action might differ if the upper versus the lower boundary of the confidence interval represented the truth. *Very serious:* similar to ‘serious’ but to a greater extent (e.g., very small sample sizes and confidence intervals crossing appreciable benefit and harm).

**Other considerations:** *Not serious:* Publication bias is undetected. *Serious/very serious:* Publication bias is strongly suspected.

**Explanations**

a. Risk of bias: We downgraded twice. All of the trials were rated as overall high or unclear risk of bias.

b. Inconsistency: We downgraded once. There is similarity in the point estimates with overlapping confidence intervals. Statistical heterogeneity is between 30% and 60% (i.e., I2 = 54%); this could not be explained due to small subgroups and may represent moderate heterogeneity.

c. Indirectness: We did not downgrade because the trials were conducted in different countries (high and low or lower-middle income).

d. Imprecision: We downgraded once (studies have small sample sizes ranging from 5 to 125 participants per group). The point estimate reached the pre-specified threshold for what may be considered clinically important (MD ≥ 10% scale range or SMD ≥ 0.2). The confidence interval does not cross null; however, one of the boundaries crosses the pre-specified threshold (≥ 10% scale range or SMD ≥ 0.2).

e. Inconsistency: We did not downgrade. The point estimates are similar with overlapping confidence intervals; statistical heterogeneity is between 0% and 40%, which might not be important (i.e., I2 = 0%).

f. Indirectness: We did not downgrade because the trials were conducted in different countries (low or lower-middle income).

g. Inconsistency: We downgraded once. The point estimates are mostly in the same direction with overlapping confidence intervals. Statistical heterogeneity is between 50% and 90% (i.e., I2 = 68%). This could not be explained due to small subgroups and may represent substantial heterogeneity.

h. Imprecision: We downgraded once due to small sample size (the OIS would not have been reached). The point estimate reached the pre-specified threshold for what may be considered clinically important (MD ≥ 10% scale range or SMD ≥ 0.2). The confidence interval does not cross the null; however, one of the boundaries crosses the pre-specified threshold (≥ 10% scale range or SMD ≥ 0.2).

i. Inconsistency: We did not downgrade; however, there are no additional studies with which to compare these findings.

j. Indirectness: We downgraded once. This is a single trial from a single centre (high or upper-middle income).

k. Imprecision: We downgraded twice due to small sample size (the OIS would not have been reached).

l. Inconsistency: We downgraded once. There is similarity in the point estimates with overlapping confidence intervals. Statistical heterogeneity is between 30% and 60% (i.e., I2 = 58%); this could not be explained due to small subgroups and may represent moderate heterogeneity.

m. Inconsistency: We downgraded once. There is some overlap in the confidence intervals. Statistical heterogeneity is between 50% and 90% (i.e., I2 = 79%). This could not be explained due to small subgroups and may represent substantial heterogeneity.

n. Indirectness: We downgraded once because the trials were conducted in the same country (high-income).

o. Indirectness: We did not downgrade because the trials were conducted in different countries (high or upper-middle income).

p. Inconsistency: We downgraded once. The point estimates are or are mostly in the same direction with overlapping confidence intervals. Statistical heterogeneity is between 50% and 90% (i.e., I2 = 64%). This could not be explained due to small subgroups and may represent substantial heterogeneity.

q. Inconsistency: We did not downgrade. The point estimates are mostly similar with overlapping confidence intervals; statistical heterogeneity is between 0% and 40%, which might not be important (i.e., I2 = 30%).

r. Imprecision: We downgraded once (studies have small sample sizes ranging from 5 to 125 participants per group). The point estimate did not reach the pre-specified threshold for what may be considered clinically important (MD ≥ 10% scale range or SMD ≥ 0.2). The confidence interval does not cross null; however, one of the boundaries crosses the pre-specified threshold (≥ 10% scale range or SMD ≥ 0.2).

s. Inconsistency: We downgraded once. There is similarity in most or all of the point estimates with overlapping confidence intervals. Statistical heterogeneity is between 30% and 60% (i.e., I2 = 52%); this could not be explained due to small subgroups and may represent moderate heterogeneity.

t. Imprecision: We downgraded once (studies have small sample sizes ranging from 6 to 125 participants per group). The point estimate reached the pre-specified threshold for what may be considered clinically important (MD ≥ 10% scale range or SMD ≥ 0.2). The confidence interval does not cross null; however, one of the boundaries crosses the pre-specified threshold (≥ 10% scale range or SMD ≥ 0.2).

u. Inconsistency: We downgraded once. There are overlapping confidence intervals. Statistical heterogeneity is between 30% and 60% (i.e., I2 = 57%); this could not be explained due to small subgroups and may represent moderate heterogeneity.

v. Imprecision: We downgraded twice. The sample size is small (OIS would not have been achieved). The point estimate did not reach the pre-specified threshold for what may be considered clinically important (MD ≥ 10% scale range or SMD ≥ 0.2). The confidence interval crossed the null with the boundaries crossing the thresholds for what may be considered appreciable benefit and harm (MD ≥ 10% scale range or SMD ≥ 0.2).

w. Inconsistency: We downgraded twice. The point estimates differ without overlapping confidence intervals. Statistical heterogeneity is between 75% and 100% (i.e., I2 = 94%); this could not be explained due to small subgroups and may represent considerable heterogeneity.

x. Imprecision: We downgraded twice. The sample size is small (OIS would not have been achieved). The point estimate reached the pre-specified threshold for what may be considered clinically important (MD ≥ 10% scale range or SMD ≥ 0.2). The confidence interval crossed the null with the boundaries crossing the thresholds for what may be considered appreciable benefit and harm (MD ≥ 10% scale range or SMD ≥ 0.2).

y. Imprecision: We downgraded once (studies have sample sizes ranging from 100 to 125 participants per group). The point estimate reached the pre-specified threshold for what may be considered clinically important (MD ≥ 10% scale range or SMD ≥ 0.2). The confidence interval crosses the null.

z. Inconsistency: We downgraded once. There similarity is some of the point estimates with some overlap in the confidence intervals. Statistical heterogeneity is between 50% and 90% (i.e., I2 = 76%). This could not be explained due to small subgroups and may represent substantial heterogeneity.

aa. Imprecision: We downgraded once . The sample size is small (OIS would not have been achieved). The point estimate reached the pre-specified threshold for what may be considered clinically important (MD ≥ 10% scale range or SMD ≥ 0.2). The confidence interval crosses the null.

ab. Inconsistency: We downgraded once. There is similarity in most or all of the point estimates with overlapping confidence intervals. Statistical heterogeneity is between 30% and 60% (i.e., I2 = 49%); this could not be explained due to small subgroups and may represent moderate heterogeneity.

ac. Inconsistency: We downgraded once. The point estimates are mostly in the same direction with overlapping confidence intervals. Statistical heterogeneity is between 50% and 90% (i.e., I2 = 72%). This could not be explained due to small subgroups and may represent substantial heterogeneity.

ad. Imprecision: We downgraded once (studies have sample sizes ranging from 5 to 74 participants per group). The point estimate reached the pre-specified threshold for what may be considered clinically important (MD ≥ 10% scale range or SMD ≥ 0.2). The confidence interval crosses the null.

ae. Inconsistency: We did not downgrade. There is similarity in most of the point estimates with overlapping confidence intervals. Statistical heterogeneity is between 30% and 60% (i.e., I2 = 43%); this could not be explained due to small subgroups and may represent moderate heterogeneity.

af. Inconsistency: We did not downgrade. There is similarity in the point estimates with overlapping confidence intervals. Statistical heterogeneity is between 30% and 60% (i.e., I2 = 50%); this could not be explained due to small subgroups and may represent moderate heterogeneity.

ag. Imprecision: We downgraded once due to small sample size (the OIS would not have been reached).

ah. Inconsistency: We downgraded once. There is similarity in the point estimates with overlapping confidence intervals. Statistical heterogeneity is between 30% and 60% (i.e., I2 = 60%); this could not be explained due to small subgroups and may represent moderate heterogeneity.

ai. Imprecision: We downgraded once (studies have small sample sizes ranging from 6 to 125 participants per group).

aj. Inconsistency: We downgraded once. There is similarity in most or all of the point estimates with overlapping confidence intervals. Statistical heterogeneity is between 30% and 60% (i.e., I2 = 59%); this could not be explained due to small subgroups and may represent moderate heterogeneity.

ak. Imprecision: We downgraded once (studies have sample sizes ranging from 24 to 125 participants per group). The point estimate reached the pre-specified threshold for what may be considered clinically important (MD ≥ 10% scale range or SMD ≥ 0.2). The confidence interval does not cross the null.

al. Inconsistency: We downgraded twice. The point estimates are in the same direction with no overlap of confidence intervals. Statistical heterogeneity is between 50% and 90% (i.e., I2 = 89%); this could not be explained due to small subgroups and may represent substantial heterogeneity.

am. Inconsistency: We downgraded once. There is similarity in most of the point estimates and overlap in the confidence intervals. Statistical heterogeneity is between 50% and 90% (i.e., I2 = 78%). This could not be explained due to small subgroups and may represent substantial heterogeneity.

an. Inconsistency: We downgraded once. There is similarity in some of the point estimates and some overlap in the confidence intervals. Statistical heterogeneity is between 50% and 90% (i.e., I2 = 83%). This could not be explained due to small subgroups and may represent substantial heterogeneity.

ao. Inconsistency: We did not downgrade. The point estimates are similar with overlapping confidence intervals; statistical heterogeneity is between 0% and 40%, which might not be important (i.e., I2 = 34%).

ap. Inconsistency: We did not downgrade. The point estimates are similar with overlapping confidence intervals; statistical heterogeneity is between 0% and 40%, which might not be important (i.e., I2 = 34%).

aq. Inconsistency: We downgraded once. The point estimates differ without overlapping confidence intervals, but are in the same direction. Statistical heterogeneity is between 75% and 100% (i.e., I2 = 93%); this could not be explained due to small subgroups and may represent considerable heterogeneity.

ar. An additional report of the same trial ( Shojaei 2017, Ref. ID 22030) also assessed self-efficacy at 6 months with another scale (The Behaviour Questionnaire). We reported the estimate obtained with the Multidisciplinary Work-related LBP Predictor Questionnaire (self-efficacy subscale), since it was also used to assess self-efficacy in the immediate term (closest to 2 weeks) (Shojaei 2017, Ref. ID 25009).

as. We downgraded twice because there was high statistical heterogeneity (I2 = 81%) which could not be explained due to small subgroups. Education was favoured in Kim 2022 (SMD = -0.59; 95% CI -1.26 to 0.10); no treatment was favoured in da Silva 2014 (SMD =1.03; 95% CI -0.15 to 2.21).

**References**

1.Ayanniyi O, Ige GO. Back care education on peasant farmers suffering from chronic mechanical low back pain. Journal of Experimental and Integrative Medicine; 2015.

2.Bodes Pardo G, Lluch Girbes E,Roussel NA,Gallego Izquierdo T,Jimenez Penick V,Pecos Martin D. Pain Neurophysiology Education and Therapeutic Exercise for Patients With Chronic Low Back Pain: A Single-Blind Randomized Controlled Trial. Arch Phys Med Rehabil; 2018.

3.da Silva TMJC, da Silva NN,de Souza Rocha SH,et al. Back school program for back pain: education or physical exercise?. ConScientiae Saúde; 2014.

4.Ibrahimi Ghavamabadi L, Mohammadi A,Behzadi A,Dehaghi BF. Effectiveness of the training program on the low back pain and functional disability in industrial workers. International Journal of Health Promotion and Education; 2022.

5.Kim KS, An J,Kim JO,Lee MY,Lee BH. Effects of Pain Neuroscience Education Combined with Lumbar Stabilization Exercise on Strength and Pain in Patients with Chronic Low Back Pain: Randomized Controlled Trial. J Pers Med; 2022.

6.Miyamoto GC, Fagundes FRC,de Melo do Espirito Santo C,et al. Education With Therapeutic Alliance Did Not Improve Symptoms in Patients With Chronic Low Back Pain and Low Risk of Poor Prognosis Compared to Education Without Therapeutic Alliance: A Randomized Controlled Trial. 2021.

7.Pires D, Cruz EB,Caeiro C. Aquatic exercise and pain neurophysiology education versus aquatic exercise alone for patients with chronic low back pain: a randomized controlled trial. Clin Rehabil; 2015.

8.Saracoglu I, Arik MI,Afsar E,Gokpinar HH. The effectiveness of pain neuroscience education combined with manual therapy and home exercise for chronic low back pain: A single-blind randomized controlled trial. Physiother Theory Pract; 2020.

9.Tellez-Garcia M, de-la-Llave-Rincon AI,Salom-Moreno J,Palacios-Cena M,Ortega-Santiago R,Fernandez-de-Las-Penas C. Neuroscience education in addition to trigger point dry needling for the management of patients with mechanical chronic low back pain: A preliminary clinical trial. J Bodyw Mov Ther; 2015.

10.Zhang Y, Wan L,Wang X. The effect of health education in patients with chronic low back pain. J Int Med Res; 2014.

11.Rantonen J, Karppinen J,Vehtari A,et al. Effectiveness of three interventions for secondary prevention of low back pain in the occupational health setting - a randomised controlled trial with a natural course control. 2018.

12.Shojaei S, Sadat Tavafian S,Reza Jamshidi A,Wagner J,Reza Sepahvandi M. Social Cognitive Theory-Based Intervention and Low Back Pain among Health Care Workers in Qom Hospitals of Iran. 2017.

GRADE evidence profile 2: ***What are the benefits and harms of education/advice in the management of community-dwelling adults (including older adults aged 60 years and over) with chronic primary low back pain (with or without leg pain) compared with sham?***

| **Certainty assessment** | | | | | | | **№ of patients** | | **Effect** | | **Certainty** | **Importance** |
| --- | --- | --- | --- | --- | --- | --- | --- | --- | --- | --- | --- | --- |
| **№ of studies** | **Study design** | **Risk of bias** | **Inconsistency** | **Indirectness** | **Imprecision** | **Other considerations** | **Education or advice** | **Sham** | **Relative (95% CI)** | **Absolute (95% CI)** |  |  |
| **ALL ADULTS** | | | | | | | | | | | | |
| **Pain (high-income country, unclassified presence of leg pain) (follow-up: closest to 3 months; assessed with: NRS; benefit indicated by lower values; Scale from: 0 to 10)** | | | | | | | | | | | | |
| 1^1^ | randomised trials | very serious^a^ | not serious^b^ | serious^c^ | very serious^d^ | none | 40 | 40 | - | MD **0.22 higher** (0.05 higher to 0.39 higher) | ⨁◯◯◯ Very low | CRITICAL |
| **Pain stratified by gender** | | | | | | | | | | | | |
| 0 |  |  |  |  |  |  |  |  |  |  |  | CRITICAL |
| **Pain in trials undertaken in low to lower-middle income countries** | | | | | | | | | | | | |
| 0 |  |  |  |  |  |  |  |  |  |  |  | CRITICAL |
| **Pain stratified by race/ethnicity** | | | | | | | | | | | | |
| 0 |  |  |  |  |  |  |  |  |  |  |  | CRITICAL |
| **Function (high-income country, unclassified presence of leg pain) (follow-up: closest to 3 months; assessed with: ODI; benefit indicated by lower values; Scale from: 0 to 50)** | | | | | | | | | | | | |
| 1^1^ | randomised trials | very serious^a^ | not serious^b^ | serious^c^ | very serious^d^ | none | 40 | 40 | - | MD **0.2 higher** (5.7 lower to 6.1 higher) | ⨁◯◯◯ Very low | CRITICAL |
| **Function stratified by gender** | | | | | | | | | | | | |
| 0 |  |  |  |  |  |  |  |  |  |  |  | CRITICAL |
| **Function in trials undertaken in low to lower-middle income countries** | | | | | | | | | | | | |
| 0 |  |  |  |  |  |  |  |  |  |  |  | CRITICAL |
| **Function stratified by race/ethnicity** | | | | | | | | | | | | |
| 0 |  |  |  |  |  |  |  |  |  |  |  | CRITICAL |
|  | | | | | | | | | | | | |
|  | | | | | | | | | | | | |
| **Fear avoidance (high-income country, unclassified presence of leg pain) (follow-up: closest to 3 months; assessed with: FABQ-PA; benefit indicated by lower values; Scale from: 0 to 24)** | | | | | | | | | | | | |
| 1^1^ | randomised trials | very serious^a^ | not serious^b^ | serious^c^ | very serious^d^ | none | 40 | 40 | - | MD **5.41 higher** (0.28 higher to 10.54 higher) | ⨁◯◯◯ Very low | CRITICAL |
| **Fear avoidance (high-income country, unclassified presence of leg pain) (follow-up: closest to 3 months; assessed with: FABQ-W; benefit indicated by lower values; Scale from: 0 to 42)** | | | | | | | | | | | | |
| 1^1^ | randomised trials | very serious^a^ | not serious^b^ | serious^c^ | very serious^d^ | none | 40 | 40 | - | MD **2.64 higher** (0.54 lower to 5.82 higher) | ⨁◯◯◯ Very low | CRITICAL |
| **Fear avoidance stratified by gender** | | | | | | | | | | | | |
| 0 |  |  |  |  |  |  |  |  |  |  |  | CRITICAL |
| **Fear avoidance in trials undertaken in low to lower-middle income countries** | | | | | | | | | | | | |
| 0 |  |  |  |  |  |  |  |  |  |  |  | CRITICAL |
| **Fear avoidance stratified by race/ethnicity** | | | | | | | | | | | | |
| 0 |  |  |  |  |  |  |  |  |  |  |  | CRITICAL |
| **Health-related quality of life** | | | | | | | | | | | | |
| 0 |  |  |  |  |  |  |  |  |  |  |  | CRITICAL |
| **Depression** | | | | | | | | | | | | |
| 0 |  |  |  |  |  |  |  |  |  |  |  | CRITICAL |
| **Catastrophizing** | | | | | | | | | | | | |
| 0 |  |  |  |  |  |  |  |  |  |  |  | CRITICAL |
| **Anxiety** | | | | | | | | | | | | |
| 0 |  |  |  |  |  |  |  |  |  |  |  | CRITICAL |
| **Self-efficacy** | | | | | | | | | | | | |
| 0 |  |  |  |  |  |  |  |  |  |  |  | CRITICAL |
|  | | | | | | | | | | | | |
| **Social participation** | | | | | | | | | | | | |
| 0 |  |  |  |  |  |  |  |  |  |  |  | CRITICAL |
| **Change in use of medications** | | | | | | | | | | | | |
| 0 |  |  |  |  |  |  |  |  |  |  |  | CRITICAL |
| **Adverse events/harms** | | | | | | | | | | | | |
| 0 |  |  |  |  |  |  |  |  |  |  |  | CRITICAL |
| **Health literacy** | | | | | | | | | | | | |
| 0 |  |  |  |  |  |  |  |  |  |  |  | CRITICAL |
| **OLDER ADULTS (aged 60 years or more)** | | | | | | | | | | | | |
| **Pain** | | | | | | | | | | | | |
| 0 |  |  |  |  |  |  |  |  |  |  |  | CRITICAL |
| **Function** | | | | | | | | | | | | |
| 0 |  |  |  |  |  |  |  |  |  |  |  | CRITICAL |
| **Health-related quality of life** | | | | | | | | | | | | |
| 0 |  |  |  |  |  |  |  |  |  |  |  | CRITICAL |
| **Psychological functioning (depression, fear avoidance, catastrophizing, anxiety, self-efficacy)** | | | | | | | | | | | | |
| 0 |  |  |  |  |  |  |  |  |  |  |  | CRITICAL |
| **Change in use of medications** | | | | | | | | | | | | |
| 0 |  |  |  |  |  |  |  |  |  |  |  | CRITICAL |
| **Falls** | | | | | | | | | | | | |
| 0 |  |  |  |  |  |  |  |  |  |  |  | CRITICAL |
| **Adverse events/harms** | | | | | | | | | | | | |
| 0 |  |  |  |  |  |  |  |  |  |  |  | CRITICAL |

**CI:** confidence interval; **FABQ-PA:** Fear Avoidance Beliefs Questionnaire-Physical Activity outcomes**; FABQ-W:** Fear Avoidance Beliefs Questionnaire-Work outcomes**; MD:** mean difference; **NRS:** numerical rating scale; **ODI:** Oswestry Disability Index; **OIS:** Optimal Information Size

The following was used to guide the ratings.

**Risk of bias:** *Not serious:* all or most of the weight (>50%) comes from overall low risk of bias trial(s). *Serious:* some of the weight (<50%) comes from overall low risk of bias trial(s). *Very serious:* all or most of the weight (>50%) comes from overall high or unclear risk of bias trial(s).

**Inconsistency:** *Not serious:* high extent of similarity of point estimates and overlap of confidence intervals; statistical heterogeneity (I^2^) is between 0% and 40%, which might not be important. *Serious:* some extent of similarity of point estimates and overlap of confidence intervals; statistical heterogeneity (I^2^) is between 30% and 60%, which could not be explained due to small subgroups and may represent moderate heterogeneity. *Very serious:* little or no similarity of point estimates and overlap of confidence intervals; statistical heterogeneity (I^2^) is between 50% and 90% or 75% and 100%, which could not be explained due to small subgroups and may represent substantial or considerable heterogeneity, respectively.

**Indirectness:** *Not serious:* trial(s) were conducted in different countries or settings. *Serious:* trial(s) were conducted from a single country/setting. *Very serious:* evidence is not directly related to PICO question.

**Imprecision:** *Not serious:* Optimal Information Size (OIS) was reached (i.e., sample sizes with at least 200 participants per group may provide prognostic balance); and the entire confidence interval lies on one side of the threshold that may be considered clinically important (≥10% scale range or SMD ≥0.2 for continuous variables, ≥10% for binary variables), such that the clinical course of action would not differ if the upper versus the lower boundary of the confidence interval represented the truth. *Serious:* OIS would not have been reached (sample sizes with less than 200 participants per group); if the OIS was reached, the clinical course of action might differ if the upper versus the lower boundary of the confidence interval represented the truth. *Very serious:* similar to ‘serious’ but to a greater extent (e.g., very small sample sizes and confidence intervals crossing appreciable benefit and harm).

**Other considerations:** *Not serious:* Publication bias is undetected. *Serious/very serious:* Publication bias is strongly suspected.

**Explanations**

a. We downgraded twice due to two risk of bias domains with high risk and greater than two domains with unclear risk.

b. Inconsistency: We did not downgrade; however, there are no additional studies with which to compare these findings.

c. Indirectness: We downgraded once. This is a single trial from a single country (high-income).

d. Imprecision: We downgraded twice due to small sample size (OIS would have not been reached).

**References**

1.Jassi FJ, Del Antonio TT,Azevedo BO,Moraes R,George SZ,Chaves TC. Star-Shape Kinesio Taping Is Not Better Than a Minimal Intervention or Sham Kinesio Taping for Pain Intensity and Postural Control in Chronic Low Back Pain: A Randomized Controlled Trial. Arch Phys Med Rehabil; 2021.

GRADE evidence profile 3: ***What are the benefits and harms of education/advice in the management of community-dwelling adults (including older adults aged 60 years and over) with chronic primary low back pain (with or without leg pain) compared with usual care?***

| **Certainty assessment** | | | | | | | **№ of patients** | | **Effect** | | **Certainty** | **Importance** |
| --- | --- | --- | --- | --- | --- | --- | --- | --- | --- | --- | --- | --- |
| **№ of studies** | **Study design** | **Risk of bias** | **Inconsistency** | **Indirectness** | **Imprecision** | **Other considerations** | **Education or advice** | **Usual care** | **Relative (95% CI)** | **Absolute (95% CI)** |  |  |
| **ALL ADULTS** | | | | | | | | | | | | |
| **Pain (high or upper-middle income country) (follow-up: closest to 3 months; assessed with: VAS; benefit indicated by lower values; Scale from: 0 to 10)** | | | | | | | | | | | | |
| 2^1,2^ | randomised trials | very serious^a^ | serious^b^ | not serious^c^ | very serious^d^ | none | 83 | 77 | - | MD **2.49 lower** (10.73 lower to 5.75 higher) | ⨁◯◯◯ Very low | CRITICAL |
| **Pain in people with and without radicular leg pain (follow-up: closest to 3 months; assessed with: VAS; benefit indicated by lower values; Scale from: 0 to 10)** | | | | | | | | | | | | |
| 1^1^ | randomised trials | very serious^a^ | not serious^e^ | serious^f^ | very serious^g^ | none | 42 | 48 | - | MD **1.8 lower** (3.03 lower to 0.57 lower) | ⨁◯◯◯ Very low | CRITICAL |
| **Pain in people with and without non-radicular leg pain (follow-up: closest to 3 months; assessed with: VAS; benefit indicated by lower values; Scale from: 0 to 10)** | | | | | | | | | | | | |
| 1^2^ | randomised trials | very serious^a^ | not serious^e^ | serious^f^ | very serious^g^ | none | 41 | 29 | - | MD **3.1 lower** (4.14 lower to 2.06 lower) | ⨁◯◯◯ Very low | CRITICAL |
| **Pain (high-income country, either with or without non-radicular leg pain) (follow-up: closest to 6 months; assessed with: VAS; benefit indicated by lower values; Scale from: 0 to 10)** | | | | | | | | | | | | |
| 1^2^ | randomised trials | very serious^a^ | not serious^e^ | serious^f^ | very serious^g^ | none | 41 | 29 | - | MD **2.1 lower** (3.13 lower to 1.07 lower) | ⨁◯◯◯ Very low | CRITICAL |
| **Pain stratified by gender** | | | | | | | | | | | | |
| 0 |  |  |  |  |  |  |  |  |  |  |  | CRITICAL |
| **Pain in trials undertaken in low to lower-middle income countries** | | | | | | | | | | | | |
| 0 |  |  |  |  |  |  |  |  |  |  |  | CRITICAL |
|  | | | | | | | | | | | | |
| **Pain stratified by race/ethnicity** | | | | | | | | | | | | |
| 0 |  |  |  |  |  |  |  |  |  |  |  | CRITICAL |
| **Function (high-income country, either with or without non-radicular leg pain) (follow-up: closest to 3 months; assessed with: ODI; benefit indicated by lower values; Scale from: 0 to 50)** | | | | | | | | | | | | |
| 1^2^ | randomised trials | very serious^a^ | not serious^e^ | serious^f^ | very serious^g^ | none | 41 | 29 | - | MD **7.8 lower** (14.28 lower to 1.32 lower) | ⨁◯◯◯ Very low | CRITICAL |
| **Function (high-income country, either with or without non-radicular leg pain) (follow-up: closest to 6 months; assessed with: ODI; benefit indicated by lower values; Scale from: 0 to 50)** | | | | | | | | | | | | |
| 1^2^ | randomised trials | very serious^a^ | not serious^e^ | serious^f^ | very serious^g^ | none | 41 | 29 | - | MD **9.2 lower** (16.5 lower to 1.9 lower) | ⨁◯◯◯ Very low | CRITICAL |
| **Function stratified by gender** | | | | | | | | | | | | |
| 0 |  |  |  |  |  |  |  |  |  |  |  | CRITICAL |
| **Function in trials undertaken in low to lower-middle income countries** | | | | | | | | | | | | |
| 0 |  |  |  |  |  |  |  |  |  |  |  | CRITICAL |
| **Function stratified by race/ethnicity** | | | | | | | | | | | | |
| 0 |  |  |  |  |  |  |  |  |  |  |  | CRITICAL |
| **Health-related quality of life (high-income country, either with or without non-radicular leg pain) (follow-up: closest to 3 months; assessed with: SF-36 (PCS); benefit indicated by higher values; Scale from: 0 to 100)** | | | | | | | | | | | | |
| 1^2^ | randomised trials | very serious^a^ | not serious^e^ | serious^f^ | very serious^g^ | none | 41 | 29 | - | MD **2.5 higher** (1.41 lower to 6.41 higher) | ⨁◯◯◯ Very low | CRITICAL |
| **Health-related quality of life (high-income country, either with or without non-radicular leg pain) (follow-up: closest to 3 months; assessed with: SF-36 (MCS); benefit indicated by higher values; Scale from: 0 to 100)** | | | | | | | | | | | | |
| 1^2^ | randomised trials | very serious^a^ | not serious^e^ | serious^f^ | very serious^g^ | none | 41 | 29 | - | MD **9.4 higher** (2.7 higher to 16.1 higher) | ⨁◯◯◯ Very low | CRITICAL |
|  | | | | | | | | | | | | |
| **Health-related quality of life (high-income country, either with or without non-radicular leg pain) (follow-up: closest to 6 months; assessed with: SF-36 (PCS); benefit indicated by higher values; Scale from: 0 to 100)** | | | | | | | | | | | | |
| 1^2^ | randomised trials | very serious^a^ | not serious^e^ | serious^f^ | very serious^g^ | none | 41 | 29 | - | MD **2.4 higher** (1.56 lower to 6.36 higher) | ⨁◯◯◯ Very low | CRITICAL |
| **Health-related quality of life (high-income country, either with or without non-radicular leg pain) (follow-up: closest to 6 months; assessed with: SF-36 (MCS); benefit indicated by higher values; Scale from: 0 to 100)** | | | | | | | | | | | | |
| 1^2^ | randomised trials | very serious^a^ | not serious^e^ | serious^f^ | very serious^g^ | none | 41 | 29 | - | MD **7.2 higher** (0.53 higher to 13.87 higher) | ⨁◯◯◯ Very low | CRITICAL |
| **Health-related quality of life stratified by gender** | | | | | | | | | | | | |
| 0 |  |  |  |  |  |  |  |  |  |  |  | CRITICAL |
| **Health-related quality of life in trials undertaken in low to lower-middle income countries** | | | | | | | | | | | | |
| 0 |  |  |  |  |  |  |  |  |  |  |  | CRITICAL |
| **Health-related quality of life stratified by race/ethnicity** | | | | | | | | | | | | |
| 0 |  |  |  |  |  |  |  |  |  |  |  | CRITICAL |
| **Psychological functioning (depression, fear avoidance, catastrophizing, anxiety, self-efficacy)** | | | | | | | | | | | | |
| 0 |  |  |  |  |  |  |  |  |  |  |  | CRITICAL |
| **Social participation** | | | | | | | | | | | | |
| 0 |  |  |  |  |  |  |  |  |  |  |  | CRITICAL |
| **Change in use of medications** | | | | | | | | | | | | |
| 0 |  |  |  |  |  |  |  |  |  |  |  | CRITICAL |
| **Health literacy** | | | | | | | | | | | | |
| 0 |  |  |  |  |  |  |  |  |  |  |  | CRITICAL |
| **Adverse events/harms** | | | | | | | | | | | | |
| 0 |  |  |  |  |  |  |  |  |  |  |  | CRITICAL |
|  | | | | | | | | | | | | |
| **OLDER ADULTS (aged 60 years or more)** | | | | | | | | | | | | |
| **Pain** | | | | | | | | | | | | |
| 0 |  |  |  |  |  |  |  |  |  |  |  | CRITICAL |
| **Function** | | | | | | | | | | | | |
| 0 |  |  |  |  |  |  |  |  |  |  |  | CRITICAL |
| **Health-related quality of life** | | | | | | | | | | | | |
| 0 |  |  |  |  |  |  |  |  |  |  |  | CRITICAL |
| **Psychological functioning (depression, fear avoidance, catastrophizing, anxiety, self-efficacy)** | | | | | | | | | | | | |
| 0 |  |  |  |  |  |  |  |  |  |  |  | CRITICAL |
| **Change in use of medications** | | | | | | | | | | | | |
| 0 |  |  |  |  |  |  |  |  |  |  |  | CRITICAL |
| **Falls** | | | | | | | | | | | | |
| 0 |  |  |  |  |  |  |  |  |  |  |  | CRITICAL |
| **Adverse events/harms** | | | | | | | | | | | | |
| 0 |  |  |  |  |  |  |  |  |  |  |  | CRITICAL |

**CI:** confidence interval; **MD:** mean difference; **MCS:** mental component summary**; ODI:** Oswestry Disability Index; **OIS:** Optimal Information Size; **PCS:** Physical Component Summary**; SF-36:** short form health survey; **VAS:** Visual Analogue Scale

The following was used to guide the ratings.

**Risk of bias:** *Not serious:* all or most of the weight (>50%) comes from overall low risk of bias trial(s). *Serious:* some of the weight (<50%) comes from overall low risk of bias trial(s). *Very serious:* all or most of the weight (>50%) comes from overall high or unclear risk of bias trial(s).

**Inconsistency:** *Not serious:* high extent of similarity of point estimates and overlap of confidence intervals; statistical heterogeneity (I^2^) is between 0% and 40%, which might not be important. *Serious:* some extent of similarity of point estimates and overlap of confidence intervals; statistical heterogeneity (I^2^) is between 30% and 60%, which could not be explained due to small subgroups and may represent moderate heterogeneity. *Very serious:* little or no similarity of point estimates and overlap of confidence intervals; statistical heterogeneity (I^2^) is between 50% and 90% or 75% and 100%, which could not be explained due to small subgroups and may represent substantial or considerable heterogeneity, respectively.

**Indirectness:** *Not serious:* trial(s) were conducted in different countries or settings. *Serious:* trial(s) were conducted from a single country/setting. *Very serious:* evidence is not directly related to PICO question.

**Imprecision:** *Not serious:* Optimal Information Size (OIS) was reached (i.e., sample sizes with at least 200 participants per group may provide prognostic balance); and the entire confidence interval lies on one side of the threshold that may be considered clinically important (≥10% scale range or SMD ≥0.2 for continuous variables, ≥10% for binary variables), such that the clinical course of action would not differ if the upper versus the lower boundary of the confidence interval represented the truth. *Serious:* OIS would not have been reached (sample sizes with less than 200 participants per group); if the OIS was reached, the clinical course of action might differ if the upper versus the lower boundary of the confidence interval represented the truth. *Very serious:* similar to ‘serious’ but to a greater extent (e.g., very small sample sizes and confidence intervals crossing appreciable benefit and harm).

**Other considerations:** *Not serious:* Publication bias is undetected. *Serious/very serious:* Publication bias is strongly suspected.

**Explanations**

a. Risk of bias: We downgraded twice. Trials were rated as overall high or unclear risk of bias.

b. Inconsistency: We downgraded once. There is similarity in the point estimates with overlapping confidence intervals. Statistical heterogeneity is between 30% and 60% (i.e., I2 = 60%); this could not be explained due to small subgroups and may represent moderate heterogeneity.

c. Indirectness: We did not downgrade because the trials were conducted in different countries (high or upper-middle income).

d. Imprecision: We downgraded twice due to small sample size (OIS would not have been reached). The point estimate reached the pre-specified threshold for what may be considered clinically important (MD ≥ 10% scale range or SMD ≥ 0.2). The confidence interval crosses the null.

e. Inconsistency: We did not downgrade; however, there are no additional studies with which to compare these findings.

f. Indirectness: We downgraded once. This is a single trial from a single centre (high-income country).

g. Imprecision: We downgraded twice due to small sample size (the OIS would not have been reached).

**References**

1.Akca NK, Aydin G,Gumus K. Effect of Body Mechanics Brief Education in the Clinical Setting on Pain Patients with Lumbar Disc Hernia: A Randomized Controlled Trial. International Journal of Caring Sciences; 2017.

2.Morone G, Paolucci T,Alcuri MR,et al. Quality of life improved by multidisciplinary back school program in patients with chronic non-specific low back pain: a single blind randomized controlled trial. 2011.

**Online Resource 6.** Summary of findings tables

Summary of findings table 1: ***What are the benefits and harms of education/advice in the management of community-dwelling adults (including older adults aged 60 years and over) with chronic primary low back pain (with or without leg pain) compared to no intervention or interventions where the effect of education/advice could be isolated?***

| Outcomes | **Anticipated absolute effects^*^** (95% CI) | | Relative effect (95% CI) | № of participants (studies) | Certainty of the evidence (GRADE) | Comments |
| --- | --- | --- | --- | --- | --- | --- |
|  | **Risk with no treatment** | **Risk with education or advice** |  |  |  |  |
| **ALL ADULTS** | | | | | | |
| **Pain** (0 to 10 points; 0 = no pain) Time point: closest to 3 months | The mean pain score ranged from **1.20 to 5.50** | MD **1.1 lower** (1.63 lower to 0.56 lower) | - | 858 (10 RCTs)^1,2,3,4,5,6,7,8,9,10^ | ⨁◯◯◯ Very low^a,b,c,d^ | Education/advice reduces pain more than no additional treatment in the short term. |
| **Pain** (0 to 10 points; 0 = no pain) Time point: closest to 6 months | The mean pain score was **4.86** | MD **0.55 lower** (1.49 lower to 0.39 higher) | - | 148 (1 RCT)^6^ | ⨁◯◯◯ Very low^a,i,j,k^ | Education/advice does not reduce pain compared to no additional treatment in the intermediate term. |
| **Pain**  (0 to 10 points; 0 = no pain) Time point: closest to 12 months | The mean pain score was **5.37** | MD **1.35 lower** (2.34 lower to 0.36 lower) | - | 148 (1 RCT)^6^ | ⨁◯◯◯ Very low^a,i,j,k^ | Education/advice reduces pain more than no additional treatment in the long term. |
| **Pain** (0 to 100 points; 0 = no pain) Time point: closest to 2 years | The mean pain score was **40.00** | MD **8 lower** (18.14 lower to 2.14 higher) | - | 90 (1 RCT)^11^ | ⨁◯◯◯ Very low^a,i,j,k^ | Education/advice does not reduce pain compared to no additional treatment in the extra-long term. |
| **Function** Time point: closest to 3 months | The mean function score ranged from **2.20** (RMDQ, 0 to 24 points) **to 32.36** (Chronic Pain Questionnaire, 0 to 100 points) | SMD **0.51 SD lower** (0.89 lower to 0.12 lower) | - | 858 (10 RCTs)^1,2,3,4,5,6,7,8,9,10^ | ⨁◯◯◯ Very low^a,c,d,p^ | Education/advice improves function more than no additional treatment in the short term. |
| **Function** (0 to 100 points; 0 = no disability) Time point: closest to 6 months | The mean function score was **23.91** | MD **2.86 lower** (7.51 lower to 1.79 higher) | - | 148 (1 RCT)^6^ | ⨁◯◯◯ Very low^a,i,j,k^ | Education/advice does not improve function compared to no additional treatment in the intermediate term. |
| **Function** (0 to 100 points; 0 = no disability) Time point: closest to 12 months | The mean function score was **22.96** | MD **4.66 lower** (9.68 lower to 0.36 higher) | - | 148 (1 RCT)^6^ | ⨁◯◯◯ Very low^a,i,j,k^ | Education/advice does not improve function compared to no additional treatment in the long term. |
| **Function** (0 to 24 points; 0 = no disability) Time point: closest to 2 years | The mean function score was **7.40** | MD **1.5 lower** (3.42 lower to 0.42 higher) | - | 90 (1 RCT)^11^ | ⨁◯◯◯ Very low^a,i,j,k^ | Education/advice does not improve function compared to no additional treatment in the extra-long term. |
| **Health-related quality of life** (QofL) (physical component) (0 to 100 points; 0 = poor QofL) Time point: closest to 3 months | The mean health-related quality of life (physical component) score ranged from **40.00 to 63.68** | MD **24.27 higher** (12.93 higher to 35.61 higher) | - | 299 (2 RCTs)^4,10^ | ⨁◯◯◯ Very low^a,ak,c,e^ | Education/advice improves health-related quality of life (physical component) more than no additional treatment in the short term. |
| **Health-related quality of life** (QofL) (mental component) (0 to 100 points; 0 = poor QofL) Time point: closest to 3 months | The mean health-related quality of life (mental component) score ranged from **49.50 to 82.35** | MD **13.99 higher** (62.04 lower to 90.03 higher) | - | 250 (2 RCTs)^4,10^ | ⨁◯◯◯ Very low^a,al,c,x^ | Education/advice does not improve health-related quality of life (mental component) compared to no additional treatment in the short term. |
| **Fear avoidance**  Time point: closest to 3 months | The mean fear avoidance score ranged from **24.10** (TSK-11, 11 to 44 points) **to 45.72** (TSK-11, 11 to 44 points) | SMD **1.4 SD lower** (2.51 lower to 0.29 lower) | - | 142 (5 RCTs)^2,5,7,8,9^ | ⨁◯◯◯ Very low^a,ag,am,o^ | Education/advice reduces fear avoidance more than no additional treatment in the short term. |
| **Fear avoidance** (13 to 78 points; lower score = lower fear avoidance behaviours) Time point: closest to 2 years | The mean fear avoidance score was **41.00** | MD **1 lower** (7.13 lower to 5.13 higher) | - | 90 (1 RCT)^11^ | ⨁◯◯◯ Very low^a,i,j,k^ | Education/advice does not reduce fear avoidance compared to no additional treatment in the extra-long term. |
| **Catastrophizing** (0 to 52 points; 0 = no catastrophizing) Time point: closest to 3 months | The mean catastrophizing score ranged from **18.94 to 32.10** | MD **10.19 lower** (55.46 lower to 35.07 higher) | - | 91 (2 RCTs)^2,5^ | ⨁◯◯◯ Very low^a,aq,k,o^ | Education/advice does not reduce catastrophizing compared to no additional treatment in the short term. |
| **Depression** (4 to 20 points; lower score = worse depression) Time point: closest to 2 weeks | The mean depression score was **14.70** | MD **2.1 higher** (1.05 higher to 3.15 higher) | - | 125 (1 RCT)^12^ | ⨁◯◯◯ Very low^a,ag,i,j^ | Education/advice improves depression more than no additional treatment in the immediate term. |
| **Depression** (4 to 20 points; lower score = worse depression) Time point: closest to 6 months | The mean depression score was **14.50** | MD **1.5 higher** (0.5 higher to 2.5 higher) | - | 125 (1 RCT)^12^ | ⨁◯◯◯ Very low^a,ag,i,j^ | Education/advice improves depression more than no additional treatment in the intermediate term (may not reach threshold for clinical importance). |
| Anxiety | The mean anxiety was **0** | **0**  (0 to 0 ) | - | (0 studies) | - |  |
| **Self-efficacy** (7 to 35 points; lower score = lower self-efficacy) Time point: closest to 2 weeks | The mean self-efficacy score was **25.80** | MD **4.4 higher** (2.77 higher to 6.03 higher) | - | 125 (1 RCT)^12^ | ⨁◯◯◯ Very low^a,ag,i,j^ | Education/advice improves self-efficacy more than no additional treatment in the immediate term. |
| **Self-efficacy** (7 to 35 points; lower score = lower self-efficacy) Time point: closest to 6 months | The mean self-efficacy score was **26.40** | MD **1.6 higher** (0.04 higher to 3.16 higher) | - | 125 (1 RCT)^12,ar^ | ⨁◯◯◯ Very low^a,ag,i,j^ | Education/advice improves self-efficacy more than no additional treatment in the intermediate term (may not reach threshold for clinical importance). |
| **Social participation** (number of sickness absence days) Time point: closest to 2 years | The mean number of sickness absence days was **not reported** | MD **11 lower** (44 lower to 22 higher) | - | 90 (1 RCT)^11^ | ⨁◯◯◯ Very low^a,i,j,k^ | Education/advice does not reduce number of sickness absence days compared to no additional treatment in the extra-long term. |
| **Change in use of medications** | -- | **--** | - | (0 studies) | - |  |
| **Health literacy** | -- | **--** | - | (0 studies) | - |  |
| **Adverse events/harms** Time point: 2 years | The trial author reported that no adverse events were reported by participants (n=90) during the interventions. | |  | (1 RCT)^11^ | ⨁◯◯◯ Very low^a,ag,i,j^ | Education/advice does not contribute to adverse events. |
| **OLDER ADULTS (aged 60 years or more)** | | | | | | |
| **Pain** (0 to 10 points; 0 = no pain) Time point: closest to 3 months | The mean pain score ranged from **3.47 to 5.50** | MD **0.5 lower** (5.42 lower to 4.41 higher) | - | 49 (2 RCTs)^3,5^ | ⨁◯◯◯ Very low^a,e,k,o^ | Education/advice does not reduce pain in older adults compared to no additional treatment in the short term. |
| **Function** (0 to 24 points; 0 = no disability) Time point: closest to 3 months | The mean function score ranged from **7.14 to 7.18** | SMD **0.02 SD lower** (9.79 lower to 9.76 higher) | - | 49 (2 RCTs)^3,5^ | ⨁◯◯◯ Very low^a,as,c,k^ | Education/advice does not improve function in older adults compared to no additional treatment in the short term. |
| **Fear avoidance** (11 to 44 points, 0 = no fear avoidance). Time point: closest to 3 months | The mean fear avoidance score was **45.72** | SMD **0.97 SD lower** (1.68 lower to 0.27 lower) | - | 35 (1 RCT)^5^ | ⨁◯◯◯ Very low^a,i,j,k^ | Education/advice reduces fear avoidance in older adults compared to no additional treatment in the short term. |
| **Health-related quality of life** | - | **-** | - | (0 studies) | - |  |
| **Depression** | - | **-** | - | (0 studies) | - |  |
| **Catastrophizing** | - | **-** | - | (0 studies) | - |  |
| **Anxiety** | - | **-** | - | (0 studies) | - |  |
| **Self-efficacy** | - | **-** | - | (0 studies) | - |  |
| **Change in use of medications** | - | **-** | - | (0 studies) | - |  |
| **Falls** | - | **-** | - | (0 studies) | - |  |
| **Adverse events/harms** | - | **-** | - | (0 studies) | - |  |
| ***The risk in the intervention group** (and its 95% confidence interval) is based on the assumed risk in the comparison group and the **relative effect** of the intervention (and its 95% CI).  **CI:** confidence interval; **MD:** mean difference; **SMD:** standardised mean difference | | | | | | |
| **GRADE Working Group grades of evidence** **High certainty:** we are very confident that the true effect lies close to that of the estimate of the effect. **Moderate certainty:** we are moderately confident in the effect estimate: the true effect is likely to be close to the estimate of the effect, but there is a possibility that it is substantially different. **Low certainty:** our confidence in the effect estimate is limited: the true effect may be substantially different from the estimate of the effect. **Very low certainty:** we have very little confidence in the effect estimate: the true effect is likely to be substantially different from the estimate of effect. | | | | | | |

**Explanations**

a. Risk of bias: We downgraded twice. All of the trials were rated as overall high or unclear risk of bias.

b. Inconsistency: We downgraded once. There is similarity in the point estimates with overlapping confidence intervals. Statistical heterogeneity is between 30% and 60% (i.e., I2 = 54%); this could not be explained due to small subgroups and may represent moderate heterogeneity.

c. Indirectness: We did not downgrade because the trials were conducted in different countries (high and low or lower-middle income).

d. Imprecision: We downgraded once (studies have small sample sizes ranging from 5 to 125 participants per group). The point estimate reached the pre-specified threshold for what may be considered clinically important (MD ≥ 10% scale range or SMD ≥ 0.2). The confidence interval does not cross null; however, one of the boundaries crosses the pre-specified threshold (≥ 10% scale range or SMD ≥ 0.2).

e. Inconsistency: We did not downgrade. The point estimates are similar with overlapping confidence intervals; statistical heterogeneity is between 0% and 40%, which might not be important (i.e., I2 = 0%).

f. Indirectness: We did not downgrade because the trials were conducted in different countries (low or lower-middle income).

g. Inconsistency: We downgraded once. The point estimates are mostly in the same direction with overlapping confidence intervals. Statistical heterogeneity is between 50% and 90% (i.e., I2 = 68%). This could not be explained due to small subgroups and may represent substantial heterogeneity.

h. Imprecision: We downgraded once due to small sample size (the OIS would not have been reached). The point estimate reached the pre-specified threshold for what may be considered clinically important (MD ≥ 10% scale range or SMD ≥ 0.2). The confidence interval does not cross the null; however, one of the boundaries crosses the pre-specified threshold (≥ 10% scale range or SMD ≥ 0.2).

i. Inconsistency: We did not downgrade; however, there are no additional studies with which to compare these findings.

j. Indirectness: We downgraded once. This is a single trial from a single centre (high or upper-middle income).

k. Imprecision: We downgraded twice due to small sample size (the OIS would not have been reached).

l. Inconsistency: We downgraded once. There is similarity in the point estimates with overlapping confidence intervals. Statistical heterogeneity is between 30% and 60% (i.e., I2 = 58%); this could not be explained due to small subgroups and may represent moderate heterogeneity.

m. Inconsistency: We downgraded once. There is some overlap in the confidence intervals. Statistical heterogeneity is between 50% and 90% (i.e., I2 = 79%). This could not be explained due to small subgroups and may represent substantial heterogeneity.

n. Indirectness: We downgraded once because the trials were conducted in the same country (high-income).

o. Indirectness: We did not downgrade because the trials were conducted in different countries (high or upper-middle income).

p. Inconsistency: We downgraded once. The point estimates are or are mostly in the same direction with overlapping confidence intervals. Statistical heterogeneity is between 50% and 90% (i.e., I2 = 64%). This could not be explained due to small subgroups and may represent substantial heterogeneity.

q. Inconsistency: We did not downgrade. The point estimates are mostly similar with overlapping confidence intervals; statistical heterogeneity is between 0% and 40%, which might not be important (i.e., I2 = 30%).

r. Imprecision: We downgraded once (studies have small sample sizes ranging from 5 to 125 participants per group). The point estimate did not reach the pre-specified threshold for what may be considered clinically important (MD ≥ 10% scale range or SMD ≥ 0.2). The confidence interval does not cross null; however, one of the boundaries crosses the pre-specified threshold (≥ 10% scale range or SMD ≥ 0.2).

s. Inconsistency: We downgraded once. There is similarity in most or all of the point estimates with overlapping confidence intervals. Statistical heterogeneity is between 30% and 60% (i.e., I2 = 52%); this could not be explained due to small subgroups and may represent moderate heterogeneity.

t. Imprecision: We downgraded once (studies have small sample sizes ranging from 6 to 125 participants per group). The point estimate reached the pre-specified threshold for what may be considered clinically important (MD ≥ 10% scale range or SMD ≥ 0.2). The confidence interval does not cross null; however, one of the boundaries crosses the pre-specified threshold (≥ 10% scale range or SMD ≥ 0.2).

u. Inconsistency: We downgraded once. There are overlapping confidence intervals. Statistical heterogeneity is between 30% and 60% (i.e., I2 = 57%); this could not be explained due to small subgroups and may represent moderate heterogeneity.

v. Imprecision: We downgraded twice. The sample size is small (OIS would not have been achieved). The point estimate did not reach the pre-specified threshold for what may be considered clinically important (MD ≥ 10% scale range or SMD ≥ 0.2). The confidence interval crossed the null with the boundaries crossing the thresholds for what may be considered appreciable benefit and harm (MD ≥ 10% scale range or SMD ≥ 0.2).

w. Inconsistency: We downgraded twice. The point estimates differ without overlapping confidence intervals. Statistical heterogeneity is between 75% and 100% (i.e., I2 = 94%); this could not be explained due to small subgroups and may represent considerable heterogeneity.

x. Imprecision: We downgraded twice. The sample size is small (OIS would not have been achieved). The point estimate reached the pre-specified threshold for what may be considered clinically important (MD ≥ 10% scale range or SMD ≥ 0.2). The confidence interval crossed the null with the boundaries crossing the thresholds for what may be considered appreciable benefit and harm (MD ≥ 10% scale range or SMD ≥ 0.2).

y. Imprecision: We downgraded once (studies have sample sizes ranging from 100 to 125 participants per group). The point estimate reached the pre-specified threshold for what may be considered clinically important (MD ≥ 10% scale range or SMD ≥ 0.2). The confidence interval crosses the null.

z. Inconsistency: We downgraded once. There similarity is some of the point estimates with some overlap in the confidence intervals. Statistical heterogeneity is between 50% and 90% (i.e., I2 = 76%). This could not be explained due to small subgroups and may represent substantial heterogeneity.

aa. Imprecision: We downgraded once . The sample size is small (OIS would not have been achieved). The point estimate reached the pre-specified threshold for what may be considered clinically important (MD ≥ 10% scale range or SMD ≥ 0.2). The confidence interval crosses the null.

ab. Inconsistency: We downgraded once. There is similarity in most or all of the point estimates with overlapping confidence intervals. Statistical heterogeneity is between 30% and 60% (i.e., I2 = 49%); this could not be explained due to small subgroups and may represent moderate heterogeneity.

ac. Inconsistency: We downgraded once. The point estimates are mostly in the same direction with overlapping confidence intervals. Statistical heterogeneity is between 50% and 90% (i.e., I2 = 72%). This could not be explained due to small subgroups and may represent substantial heterogeneity.

ad. Imprecision: We downgraded once (studies have sample sizes ranging from 5 to 74 participants per group). The point estimate reached the pre-specified threshold for what may be considered clinically important (MD ≥ 10% scale range or SMD ≥ 0.2). The confidence interval crosses the null.

ae. Inconsistency: We did not downgrade. There is similarity in most of the point estimates with overlapping confidence intervals. Statistical heterogeneity is between 30% and 60% (i.e., I2 = 43%); this could not be explained due to small subgroups and may represent moderate heterogeneity.

af. Inconsistency: We did not downgrade. There is similarity in the point estimates with overlapping confidence intervals. Statistical heterogeneity is between 30% and 60% (i.e., I2 = 50%); this could not be explained due to small subgroups and may represent moderate heterogeneity.

ag. Imprecision: We downgraded once due to small sample size (the OIS would not have been reached).

ah. Inconsistency: We downgraded once. There is similarity in the point estimates with overlapping confidence intervals. Statistical heterogeneity is between 30% and 60% (i.e., I2 = 60%); this could not be explained due to small subgroups and may represent moderate heterogeneity.

ai. Imprecision: We downgraded once (studies have small sample sizes ranging from 6 to 125 participants per group).

aj. Inconsistency: We downgraded once. There is similarity in most or all of the point estimates with overlapping confidence intervals. Statistical heterogeneity is between 30% and 60% (i.e., I2 = 59%); this could not be explained due to small subgroups and may represent moderate heterogeneity.

ak. Imprecision: We downgraded once (studies have sample sizes ranging from 24 to 125 participants per group). The point estimate reached the pre-specified threshold for what may be considered clinically important (MD ≥ 10% scale range or SMD ≥ 0.2). The confidence interval does not cross the null.

al. Inconsistency: We downgraded twice. The point estimates are in the same direction with no overlap of confidence intervals. Statistical heterogeneity is between 50% and 90% (i.e., I2 = 89%); this could not be explained due to small subgroups and may represent substantial heterogeneity.

am. Inconsistency: We downgraded once. There is similarity in most of the point estimates and overlap in the confidence intervals. Statistical heterogeneity is between 50% and 90% (i.e., I2 = 78%). This could not be explained due to small subgroups and may represent substantial heterogeneity.

an. Inconsistency: We downgraded once. There is similarity in some of the point estimates and some overlap in the confidence intervals. Statistical heterogeneity is between 50% and 90% (i.e., I2 = 83%). This could not be explained due to small subgroups and may represent substantial heterogeneity.

ao. Inconsistency: We did not downgrade. The point estimates are similar with overlapping confidence intervals; statistical heterogeneity is between 0% and 40%, which might not be important (i.e., I2 = 34%).

ap. Inconsistency: We did not downgrade. The point estimates are similar with overlapping confidence intervals; statistical heterogeneity is between 0% and 40%, which might not be important (i.e., I2 = 34%).

aq. Inconsistency: We downgraded once. The point estimates differ without overlapping confidence intervals, but are in the same direction. Statistical heterogeneity is between 75% and 100% (i.e., I2 = 93%); this could not be explained due to small subgroups and may represent considerable heterogeneity.

ar. An additional report of the same trial ( Shojaei 2017, Ref. ID 22030) also assessed self-efficacy at 6 months with another scale (The Behaviour Questionnaire). We reported the estimate obtained with the Multidisciplinary Work-related LBP Predictor Questionnaire (self-efficacy subscale), since it was also used to assess self-efficacy in the immediate term (closest to 2 weeks) (Shojaei 2017, Ref. ID 25009).

as. We downgraded twice because there was high statistical heterogeneity (I2 = 81%) which could not be explained due to small subgroups. Education was favoured in Kim 2022 (SMD = -0.59; 95% CI -1.26 to 0.10); no treatment was favoured in da Silva 2014 (SMD =1.03; 95% CI -0.15 to 2.21).

**References**

1.Ayanniyi O, Ige GO. Back care education on peasant farmers suffering from chronic mechanical low back pain. Journal of Experimental and Integrative Medicine; 2015.

2.Bodes Pardo G, Lluch Girbes E,Roussel NA,Gallego Izquierdo T,Jimenez Penick V,Pecos Martin D. Pain Neurophysiology Education and Therapeutic Exercise for Patients With Chronic Low Back Pain: A Single-Blind Randomized Controlled Trial. Arch Phys Med Rehabil; 2018.

3.da Silva TMJC, da Silva NN,de Souza Rocha SH,et al. Back school program for back pain: education or physical exercise?. ConScientiae Saúde; 2014.

4.Ibrahimi Ghavamabadi L, Mohammadi A,Behzadi A,Dehaghi BF. Effectiveness of the training program on the low back pain and functional disability in industrial workers. International Journal of Health Promotion and Education; 2022.

5.Kim KS, An J,Kim JO,Lee MY,Lee BH. Effects of Pain Neuroscience Education Combined with Lumbar Stabilization Exercise on Strength and Pain in Patients with Chronic Low Back Pain: Randomized Controlled Trial. J Pers Med; 2022.

6.Miyamoto GC, Fagundes FRC,de Melo do Espirito Santo C,et al. Education With Therapeutic Alliance Did Not Improve Symptoms in Patients With Chronic Low Back Pain and Low Risk of Poor Prognosis Compared to Education Without Therapeutic Alliance: A Randomized Controlled Trial. 2021.

7.Pires D, Cruz EB,Caeiro C. Aquatic exercise and pain neurophysiology education versus aquatic exercise alone for patients with chronic low back pain: a randomized controlled trial. Clin Rehabil; 2015.

8.Saracoglu I, Arik MI,Afsar E,Gokpinar HH. The effectiveness of pain neuroscience education combined with manual therapy and home exercise for chronic low back pain: A single-blind randomized controlled trial. Physiother Theory Pract; 2020.

9.Tellez-Garcia M, de-la-Llave-Rincon AI,Salom-Moreno J,Palacios-Cena M,Ortega-Santiago R,Fernandez-de-Las-Penas C. Neuroscience education in addition to trigger point dry needling for the management of patients with mechanical chronic low back pain: A preliminary clinical trial. J Bodyw Mov Ther; 2015.

10.Zhang Y, Wan L,Wang X. The effect of health education in patients with chronic low back pain. J Int Med Res; 2014.

11.Rantonen J, Karppinen J,Vehtari A,et al. Effectiveness of three interventions for secondary prevention of low back pain in the occupational health setting - a randomised controlled trial with a natural course control. 2018.

12.Shojaei S, Sadat Tavafian S,Reza Jamshidi A,Wagner J,Reza Sepahvandi M. Social Cognitive Theory-Based Intervention and Low Back Pain among Health Care Workers in Qom Hospitals of Iran. 2017.

Summary of findings table 2: ***What are the benefits and harms of education/advice in the management of community-dwelling adults (including older adults aged 60 years and over) with chronic primary low back pain (with or without leg pain) compared with sham?***

| Outcomes | **Anticipated absolute effects^*^** (95% CI) | | Relative effect (95% CI) | № of participants (studies) | Certainty of the evidence (GRADE) | Comments |
| --- | --- | --- | --- | --- | --- | --- |
|  | **Risk with sham** | **Risk with education or advice** |  |  |  |  |
| **ALL ADULTS** | | | | | | |
| **Pain** (0 to 10 points; 0 = no pain) Time point: closest to 3 months | The mean pain score was **3.13** | MD **0.22 higher** (0.05 higher to 0.39 higher) | - | 80 (1 RCT)^1^ | ⨁◯◯◯ Very low^a,b,c,d^ | Sham reduced pain compared to education/advice in the short term (may not reach clinically important threshold). |
| **Function** (0 to 100 points; 0 = no disability) Time point: closest to 3 months | The mean function score was **9.25** | MD **0.2 higher** (5.7 lower to 6.1 higher) | - | 80 (1 RCT)^1^ | ⨁◯◯◯ Very low^a,b,c,d^ | Education/advice does not improve function compared to sham in the short term. |
| **Fear avoidance** (physical activity) (0 to 24 points; lower score = lower fear avoidance behaviours) Time point: closest to 3 months | The mean fear avoidance score was **11.48** | MD **5.41 higher** (0.28 higher to 10.54 higher) | - | 80 (1 RCT)^1^ | ⨁◯◯◯ Very low^a,b,c,d^ | Sham reduces fear avoidance (related to physical activity) more than education/advice in the short term. |
| **Fear avoidance** (work) (0 to 42 points; lower score = lower fear avoidance behaviours) Time point: closest to 3 months | The mean fear avoidance score was **9.67** | MD **2.64 higher** (0.54 lower to 5.82 higher) | - | 80 (1 RCT)^1^ | ⨁◯◯◯ Very low^a,b,c,d^ | Education/advice does not reduce fear avoidance (related to work) compared to sham in the short term. |
| **Health-related quality of life** | - | **-** | - | (0 studies) | - |  |
| **Depression** | - | **-** | - | (0 studies) | - |  |
| **Catastrophizing** | - | **-** | - | (0 studies) | - |  |
| **Anxiety** | - | **-** | - | (0 studies) | - |  |
| **Self-efficacy** | - | **-** | - | (0 studies) | - |  |
| **Social participation** | - | **-** | - | (0 studies) | - |  |
| **Change in use of medications** | - | **-** | - | (0 studies) | - |  |
| **Adverse events/harms** | - | **-** | - | (0 studies) | - |  |
| **Health literacy** | - | **-** | - | (0 studies) | - |  |
| **OLDER ADULTS (aged 60 years or more)** | | | | | | |
| **Pain** | - | **-** | - | (0 studies) | - |  |
| **Function** | - | **-** | - | (0 studies) | - |  |
| **Health-related quality of life** | - | **-** | - | (0 studies) | - |  |
| **Psychological functioning** (depression, fear avoidance, catastrophizing, anxiety, self-efficacy) | - | **-** | - | (0 studies) | - |  |
| **Change in use of medications** | - | **-** | - | (0 studies) | - |  |
| **Falls** | - | **-** | - | (0 studies) | - |  |
| **Adverse events/harms** | - | **-** | - | (0 studies) | - |  |
| ***The risk in the intervention group** (and its 95% confidence interval) is based on the assumed risk in the comparison group and the **relative effect** of the intervention (and its 95% CI).  **CI:** confidence interval; **MD:** mean difference | | | | | | |
| **GRADE Working Group grades of evidence** **High certainty:** we are very confident that the true effect lies close to that of the estimate of the effect. **Moderate certainty:** we are moderately confident in the effect estimate: the true effect is likely to be close to the estimate of the effect, but there is a possibility that it is substantially different. **Low certainty:** our confidence in the effect estimate is limited: the true effect may be substantially different from the estimate of the effect. **Very low certainty:** we have very little confidence in the effect estimate: the true effect is likely to be substantially different from the estimate of effect. | | | | | | |

**Explanations**

a. We downgraded twice due to two risk of bias domains with high risk and greater than two domains with unclear risk.

b. Inconsistency: We did not downgrade; however, there are no additional studies with which to compare these findings.

c. Indirectness: We downgraded once. This is a single trial from a single country (high-income).

d. Imprecision: We downgraded twice due to small sample size (OIS would have not been reached).

**References**

1.Jassi FJ, Del Antonio TT,Azevedo BO,Moraes R,George SZ,Chaves TC. Star-Shape Kinesio Taping Is Not Better Than a Minimal Intervention or Sham Kinesio Taping for Pain Intensity and Postural Control in Chronic Low Back Pain: A Randomized Controlled Trial. Arch Phys Med Rehabil; 2021.

Summary of findings table 3: ***What are the benefits and harms of education/advice in the management of community-dwelling adults (including older adults aged 60 years and over) with chronic primary low back pain (with or without leg pain) compared with usual care?***

| Outcomes | **Anticipated absolute effects^*^** (95% CI) | | Relative effect (95% CI) | № of participants (studies) | Certainty of the evidence (GRADE) | Comments |
| --- | --- | --- | --- | --- | --- | --- |
|  | **Risk with usual care** | **Risk with education or advice** |  |  |  |  |
| **ALL ADULTS** | | | | | | |
| **Pain** (0 to 10 points; 0 = no pain) Time point: closest to 3 months | The mean pain score ranged from **6.30 to 7.60** | MD **2.49 lower** (10.73 lower to 5.75 higher) | - | 160 (2 RCTs)^1,2^ | ⨁◯◯◯ Very low^a,b,c,d^ | Education/advice does not reduce pain compared to usual care in the short term. |
| **Pain** (0 to 10 points; 0 = no pain) Time point: closest to 6 months | The mean pain score was **6.50** | MD **2.1 lower** (3.13 lower to 1.07 lower) | - | 70 (1 RCT)^2^ | ⨁◯◯◯ Very low^a,e,f,g^ | Education/advice reduces pain more than usual care in the intermediate term. |
| **Function** (0 to 50 points; 0 = no disability) Time point: closest to 3 months | The mean function score was **25.80** | MD **7.8 lower** (14.28 lower to 1.32 lower) | - | 70 (1 RCT)^2^ | ⨁◯◯◯ Very low^a,e,f,g^ | Education/advice improves function more than usual care in the short term. |
| **Function** (0 to 50 points; 0 = no disability) Time point: closest to 6 months | The mean function score was **26.00** | MD **9.2 lower** (16.5 lower to 1.9 lower) | - | 70 (1 RCT)^2^ | ⨁◯◯◯ Very low^a,e,f,g^ | Education/advice improves function more than usual care in the intermediate term. |
| **Health-related quality of life** (QofL) (physical component) (0 to 100 points; 0 = poor QofL) Time point: closest to 3 months | The mean health-related quality of life (physical component) score was **43.00** | MD **2.5 higher** (1.41 lower to 6.41 higher) | - | 70 (1 RCT)^2^ | ⨁◯◯◯ Very low^a,e,f,g^ | Education/advice does not improve health-related quality of life (physical component) compared to usual care in the short term. |
| **Health-related quality of life** (QofL) (mental component) (0 to 100 points; 0 = poor QofL) Time point: closest to 3 months | The mean health-related quality of life (mental component) score was **37.90** | MD **9.4 higher** (2.7 higher to 16.1 higher) | - | 70 (1 RCT)^2^ | ⨁◯◯◯ Very low^a,e,f,g^ | Education/advice improves health-related quality of life (mental component) compared to usual care in the short term (may not be a clinically important amount). |
| **Health-related quality of life** (QofL) (physical component) (0 to 100 points; 0 = poor QofL) Time point: closest to 6 months | The mean health-related quality of life (physical component) score was **42.60** | MD **2.4 higher** (1.56 lower to 6.36 higher) | - | 70 (1 RCT)^2^ | ⨁◯◯◯ Very low^a,e,f,g^ | Education/advice does not improve health-related quality of life (physical component) compared to usual care in the intermediate term. |
| **Health-related quality of life** (QofL) (mental component) (0 to 100 points; 0 = poor QofL) Time point: closest to 6 months | The mean health-related quality of life (mental component) score was **39.90** | MD **7.2 higher** (0.53 higher to 13.87 higher) | - | 70 (1 RCT)^2^ | ⨁◯◯◯ Very low^a,e,f,g^ | Education/advice improves health-related quality of life (mental component) compared to usual care in the intermediate term (may not be a clinically important amount). |
| **Psychological** functioning (depression, fear avoidance, catastrophizing, anxiety, self-efficacy) | - | **-** | - | (0 studies) | - |  |
| **Social participation** | - | **-** | - | (0 studies) | - |  |
| **Change in use of medications** | - | **-** | - | (0 studies) | - |  |
| **Health literacy** | - | **-** | - | (0 studies) | - |  |
| **Adverse events/harms** | - | **-** | - | (0 studies) | - |  |
| **OLDER ADULTS (aged 60 years or more)** | | | | | | |
| **Pain** | - | **-** | - | (0 studies) | - |  |
| **Function** | - | **-** | - | (0 studies) | - |  |
| **Health-related quality of life** | - | **-** | - | (0 studies) | - |  |
| **Psychological functioning** (depression, fear avoidance, catastrophizing, anxiety, self-efficacy) | - | **-** ) | - | (0 studies) | - |  |
| **Change in use of medications** | - | **-** | - | (0 studies) | - |  |
| **Falls** | - | **-** | - | (0 studies) | - |  |
| **Adverse events/harms** | - | **-** | - | (0 studies) | - |  |
| ***The risk in the intervention group** (and its 95% confidence interval) is based on the assumed risk in the comparison group and the **relative effect** of the intervention (and its 95% CI).  **CI:** confidence interval; **MD:** mean difference | | | | | | |
| **GRADE Working Group grades of evidence** **High certainty:** we are very confident that the true effect lies close to that of the estimate of the effect. **Moderate certainty:** we are moderately confident in the effect estimate: the true effect is likely to be close to the estimate of the effect, but there is a possibility that it is substantially different. **Low certainty:** our confidence in the effect estimate is limited: the true effect may be substantially different from the estimate of the effect. **Very low certainty:** we have very little confidence in the effect estimate: the true effect is likely to be substantially different from the estimate of effect. | | | | | | |

**Explanations**

a. Risk of bias: We downgraded twice. Trials were rated as overall high or unclear risk of bias.

b. Inconsistency: We downgraded once. There is similarity in the point estimates with overlapping confidence intervals. Statistical heterogeneity is between 30% and 60% (i.e., I2 = 60%); this could not be explained due to small subgroups and may represent moderate heterogeneity.

c. Indirectness: We did not downgrade because the trials were conducted in different countries (high or upper-middle income).

d. Imprecision: We downgraded twice due to small sample size (OIS would not have been reached). The point estimate reached the pre-specified threshold for what may be considered clinically important (MD ≥ 10% scale range or SMD ≥ 0.2). The confidence interval crosses the null.

e. Inconsistency: We did not downgrade; however, there are no additional studies with which to compare these findings.

f. Indirectness: We downgraded once. This is a single trial from a single centre (high-income country).

g. Imprecision: We downgraded twice due to small sample size (the OIS would not have been reached).

**References**

1.Akca NK, Aydin G,Gumus K. Effect of Body Mechanics Brief Education in the Clinical Setting on Pain Patients with Lumbar Disc Hernia: A Randomized Controlled Trial. International Journal of Caring Sciences; 2017.

2.Morone G, Paolucci T,Alcuri MR,et al. Quality of life improved by multidisciplinary back school program in patients with chronic non-specific low back pain: a single blind randomized controlled trial. 2011.

**Online Resource 7.** Meta-analyses (including subgroup analyses)

**References**

1. Akca NK, Aydin G, Gumus K. Effect of Body Mechanics Brief Education in the Clinical Setting on Pain Patients with Lumbar Disc Hernia: A Randomized Controlled Trial. *International Journal of Caring Sciences*. 2017;10(3):1498-1506.

2. Ayanniyi O, Ige GO. Back care education on peasant farmers suffering from chronic mechanical low back pain. *Journal of Experimental and Integrative Medicine*. 2015;5(4):215-221.

3. Bodes Pardo G, Lluch Girbes E, Roussel NA, Gallego Izquierdo T, Jimenez Penick V, Pecos Martin D. Pain Neurophysiology Education and Therapeutic Exercise for Patients With Chronic Low Back Pain: A Single-Blind Randomized Controlled Trial. *Arch Phys Med Rehabil*. Feb 2018;99(2):338-347. doi:10.1016/j.apmr.2017.10.016

4. da Silva TMJC, da Silva NN, de Souza Rocha SH, et al. Back school program for back pain: education or physical exercise? *ConScientiae Saúde*. 2014;13(4):506-515.

5. Ibrahimi Ghavamabadi L, Mohammadi A, Behzadi A, Dehaghi BF. Effectiveness of the training program on the low back pain and functional disability in industrial workers. *International Journal of Health Promotion and Education*. 2022;

6. Jassi FJ, Del Antonio TT, Azevedo BO, Moraes R, George SZ, Chaves TC. Star-Shape Kinesio Taping Is Not Better Than a Minimal Intervention or Sham Kinesio Taping for Pain Intensity and Postural Control in Chronic Low Back Pain: A Randomized Controlled Trial. *Arch Phys Med Rehabil*. Jul 2021;102(7):1352-1360 e3. doi:10.1016/j.apmr.2021.03.007

7. Kim KS, An J, Kim JO, Lee MY, Lee BH. Effects of Pain Neuroscience Education Combined with Lumbar Stabilization Exercise on Strength and Pain in Patients with Chronic Low Back Pain: Randomized Controlled Trial. *J Pers Med*. Feb 17 2022;12(2)doi:10.3390/jpm12020303

8. Miyamoto GC, Fagundes FRC, de Melo do Espirito Santo C, et al. Education With Therapeutic Alliance Did Not Improve Symptoms in Patients With Chronic Low Back Pain and Low Risk of Poor Prognosis Compared to Education Without Therapeutic Alliance: A Randomized Controlled Trial. *J Orthop Sports Phys Ther*. Aug 2021;51(8):392-400. doi:10.2519/jospt.2021.9636

9. Morone G, Paolucci T, Alcuri MR, et al. Quality of life improved by multidisciplinary back school program in patients with chronic non-specific low back pain: a single blind randomized controlled trial. *Eur J Phys Rehabil Med*. Dec 2011;47(4):533-41.

10. Pires D, Cruz EB, Caeiro C. Aquatic exercise and pain neurophysiology education versus aquatic exercise alone for patients with chronic low back pain: a randomized controlled trial. *Clin Rehabil*. Jun 2015;29(6):538-47. doi:10.1177/0269215514549033

11. Rantonen J, Karppinen J, Vehtari A, et al. Effectiveness of three interventions for secondary prevention of low back pain in the occupational health setting - a randomised controlled trial with a natural course control. *BMC Public Health*. May 8 2018;18(1):598. doi:10.1186/s12889-018-5476-8

12. Saracoglu I, Arik MI, Afsar E, Gokpinar HH. The effectiveness of pain neuroscience education combined with manual therapy and home exercise for chronic low back pain: A single-blind randomized controlled trial. *Physiother Theory Pract*. Aug 19 2020:1-11. doi:10.1080/09593985.2020.1809046

13. Shojaei S, Sadat Tavafian S, Reza Jamshidi A, Wagner J. A Multidisciplinary Workplace Intervention for Chronic Low Back Pain among Nursing Assistants in Iran. *Asian Spine Journal* 2017;11(3):419-426.

14. Shojaei S, Sadat Tavafian S, Reza Jamshidi A, Wagner J, Reza Sepahvandi M. Social Cognitive Theory-Based Intervention and Low Back Pain among Health Care Workers in Qom Hospitals of Iran. *International Journal of Musculoskeletal Pain Prevention*. 2017;2(1):193-201.

15. Tellez-Garcia M, de-la-Llave-Rincon AI, Salom-Moreno J, Palacios-Cena M, Ortega-Santiago R, Fernandez-de-Las-Penas C. Neuroscience education in addition to trigger point dry needling for the management of patients with mechanical chronic low back pain: A preliminary clinical trial. *J Bodyw Mov Ther*. Jul 2015;19(3):464-72. doi:10.1016/j.jbmt.2014.11.012

16. Zhang Y, Wan L, Wang X. The effect of health education in patients with chronic low back pain. *J Int Med Res*. Jun 2014;42(3):815-20. doi:10.1177/0300060514527059

17. Antunes M, Bertolini S. Comparison between the intervention with back school and postural reeducation in the posture of elderly people with low back pain. *Journal of Clinical Rheumatology*. 2021;27(Supplement 1):S113.

18. Ferreira GE, Lin CC, Stevens ML, et al. TOPS - a randomized controlled trial of exercise and education to prevent recurrence of low back pain: statistical analysis plan. *Braz J Phys Ther*. Jul - Aug 2020;24(4):373-380. doi:10.1016/j.bjpt.2020.05.001

19. Gul H, Erel S. Effect of therapeutic neuroscience education combined with physical therapy in individuals with chronic low back pain *Turkish journal of physiotherapy and rehabilitation*. 2019;30(2):S45-S46.

20. Effects of Patient Education Manual in Patient With Chronic Low Back Pain. Identifier: NCT04600843. National Library of Medicine. 2022. <https://clinicaltrials.gov/ct2/show/NCT04600843>

21. Attanayake AMP, Somarathna KIWK, Vyas GH, Dash SC. Clinical evaluation of selected Yogic procedures in individuals with low back pain. *AYU Journal*. 2010;31(2):245-50.

22. Ghadyani L, Tavafian SS, Kazemnejad A, Wagner J. Effectiveness of Multidisciplinary Group-Based Intervention versus Individual Physiotherapy for Improving Chronic Low Back Pain in Nursing Staff: A Clinical Trial with 3- and 6-Month Follow-Up Visits from Tehran, Iran. *Asian Spine J*. Jun 2017;11(3):396-404. doi:10.4184/asj.2017.11.3.396

23. Gul H, Erel S, Toraman NF. Physiotherapy combined with therapeutic neuroscience education versus physiotherapy alone for patients with chronic low back pain: A pilot, randomized-controlled trial. *Turk J Phys Med Rehabil*. Sep 2021;67(3):283-290. doi:10.5606/tftrd.2021.5556

24. Saxena P, Thiyagarajan S. Scapular and Pelvic PNF Pattern for Female Physical Education Students with Low Back Pain. *Indian Journal of Physiotherapy and Occupational Therapy*. 2020;14(4):127-33.

25. Sharafkhani N, Khorsandi M, Shamsi M, Ranjbaran M. The Effect of an Educational Intervention Program on the Adoption of Low Back Pain Preventive Behaviors in Nurses: An Application of the Health Belief Model. *Global Spine J*. Feb 2016;6(1):29-34. doi:10.1055/s-0035-1555658

26. Aghilinejad M, Bahrami-Ahmadi A, Kabir-Mokamelkhah E, Sarebanha S, Hosseini HR, Sadeghi Z. The effect of three ergonomics training programs on the prevalence of low-back pain among workers of an Iranian automobile factory: a randomized clinical trial. *Int J Occup Environ Med*. Apr 2014;5(2):65-71.

27. Anan T, Kajiki S, Oka H, et al. Effects of an Artificial Intelligence-Assisted Health Program on Workers With Neck/Shoulder Pain/Stiffness and Low Back Pain: Randomized Controlled Trial. *JMIR Mhealth Uhealth*. Sep 24 2021;9(9):e27535. doi:10.2196/27535

28. Baumeister H, Paganini S, Sander LB, et al. Effectiveness of a Guided Internet- and Mobile-Based Intervention for Patients with Chronic Back Pain and Depression (WARD-BP): A Multicenter, Pragmatic Randomized Controlled Trial. *Psychother Psychosom*. 2021;90(4):255-268. doi:10.1159/000511881

29. Buhrman M, Faltenhag S, Strom L, Andersson G. Controlled trial of Internet-based treatment with telephone support for chronic back pain. *Pain*. Oct 2004;111(3):368-377. doi:10.1016/j.pain.2004.07.021

30. Corsinovi L, Martinelli E, Fonte G, et al. Efficacy of oxycodone/acetaminophen and codeine/acetaminophen vs. conventional therapy in elderly women with persistent, moderate to severe osteoarthritis-related pain. *Arch Gerontol Geriatr*. Nov-Dec 2009;49(3):378-82. doi:10.1016/j.archger.2008.12.003

31. Dagenais S, Hayflinger DC, Mayer JM. Economic Evaluation of an Extended Telehealth Worksite Exercise Intervention to Reduce Lost Work Time from Low Back Pain in Career Firefighters. *J Occup Rehabil*. Jun 2021;31(2):431-443. doi:10.1007/s10926-020-09933-8

32. Delitto A, Patterson CG, Stevans JM, et al. Stratified care to prevent chronic low back pain in high-risk patients: The TARGET trial. A multi-site pragmatic cluster randomized trial. *EClinicalMedicine*. Apr 2021;34:100795. doi:10.1016/j.eclinm.2021.100795

33. Dwyer CP, MacNeela P, Durand H, et al. Effects of Biopsychosocial Education on the Clinical Judgments of Medical Students and GP Trainees Regarding Future Risk of Disability in Chronic Lower Back Pain: A Randomized Control Trial. *Pain Med*. May 1 2020;21(5):939-950. doi:10.1093/pm/pnz284

34. Foster NE, Konstantinou K, Lewis M, et al. Stratified versus usual care for the management of primary care patients with sciatica: the SCOPiC RCT. *Health Technol Assess*. Oct 2020;24(49):1-130. doi:10.3310/hta24490

35. Fu WJ, Ying IH. Application of nursing intervention guided by king’s interactive standard theory in patients with lumbar disc herniation undergoing conservative treatment. *Indian J Pharm Sci*. 2021;83(4 Spl issue):184-189.

36. Galan-Martin MA, Montero-Cuadrado F, Lluch-Girbes E, Coca-Lopez MC, Mayo-Iscar A, Cuesta-Vargas A. Pain Neuroscience Education and Physical Therapeutic Exercise for Patients with Chronic Spinal Pain in Spanish Physiotherapy Primary Care: A Pragmatic Randomized Controlled Trial. *J Clin Med*. Apr 22 2020;9(4)doi:10.3390/jcm9041201

37. George SZ, Teyhen DS, Wu SS, et al. Psychosocial education improves low back pain beliefs: results from a cluster randomized clinical trial (NCT00373009) in a primary prevention setting. *Eur Spine J*. Jul 2009;18(7):1050-8. doi:10.1007/s00586-009-1016-7

38. Hodges PW, Hall L, Setchell J, et al. Effect of a Consumer-Focused Website for Low Back Pain on Health Literacy, Treatment Choices, and Clinical Outcomes: Randomized Controlled Trial. *J Med Internet Res*. Jun 15 2021;23(6):e27860. doi:10.2196/27860

39. Johnsen TL, Eriksen HR, Baste V, Indahl A, Odeen M, Tveito TH. Effect of Reassuring Information About Musculoskeletal and Mental Health Complaints at the Workplace: A Cluster Randomized Trial of the atWork Intervention. *J Occup Rehabil*. Jun 2019;29(2):274-285. doi:10.1007/s10926-018-9786-6

40. Katri Maria T, Laura AM, Erja P, et al. Effects of a home-based rehabilitation program in community-dwelling older people after discharge from hospital: A subgroup analysis of a randomized controlled trial. *Clin Rehabil*. Sep 2021;35(9):1257-1265. doi:10.1177/02692155211001672

41. Kazemi SS, Tavafian SS, Hiller CE, Hidarnia A, Montazeri A. The effectiveness of social media and in-person interventions for low back pain conditions in nursing personnel (SMILE). *Nurs Open*. May 2021;8(3):1220-1231. doi:10.1002/nop2.738

42. Konstantinou K, Lewis M, Dunn KM, et al. Stratified care versus usual care for management of patients presenting with sciatica in primary care (SCOPiC): a randomised controlled trial. *Lancet Rheumatol*. Jul 2020;2(7):e401-e411. doi:10.1016/S2665-9913(20)30099-0

43. Lane E, Magel JS, Thackeray A, et al. Effectiveness of training physical therapists in pain neuroscience education for patients with chronic spine pain: a cluster-randomized trial. *Pain*. May 1 2022;163(5):852-860. doi:10.1097/j.pain.0000000000002436

44. Langagergaard V, Jensen OK, Nielsen CV, et al. The comparative effects of brief or multidisciplinary intervention on return to work at 1 year in employees on sick leave due to low back pain: A randomized controlled trial. *Clin Rehabil*. Sep 2021;35(9):1290-1304. doi:10.1177/02692155211005387

45. Lee JA, Ha IH, Choi TY, et al. Evaluating the clinical application of a leaflet for clinical practice guideline in patients with lumbar herniated intervertebral discs: Randomized controlled trial. *Medicine (Baltimore)*. Dec 2017;96(51):e9406. doi:10.1097/MD.0000000000009406

46. McCurry SM, Zhu W, Von Korff M, et al. Effect of Telephone Cognitive Behavioral Therapy for Insomnia in Older Adults With Osteoarthritis Pain: A Randomized Clinical Trial. *JAMA Intern Med*. Apr 1 2021;181(4):530-538. doi:10.1001/jamainternmed.2020.9049

47. Meisel ZF, Shofer F, Dolan A, et al. A Multicentered Randomized Controlled Trial Comparing the Effectiveness of Pain Treatment Communication Tools in Emergency Department Patients With Back or Kidney Stone Pain. *Am J Public Health*. Feb 2022;112(S1):S45-S55. doi:10.2105/AJPH.2021.306511

48. Moore JE, Von Korff M, Cherkin D, Saunders K, Lorig K. A randomized trial of a cognitive-behavioral program for enhancing back pain self care in a primary care setting. *Pain*. Nov 2000;88(2):145-153. doi:10.1016/S0304-3959(00)00314-6

49. Pereira OS. Efficacy of the Santhiflex™ method of psychomotor postural re-education in the treatment of chronic low back pain. *Fisioter Mov*. 2015;28(1):117-26.

50. Pourhaji F, Delshad MH, Tavafian SS, Niknami S, Pourhaji F. Effects of educational program based on Precede-Proceed model in promoting low back pain behaviors (EPPLBP) in health care workers Shahid Beheshti University of medical sciences: randomized trial. *Heliyon*. 2020;6:e05236.

51. Sandal LF, Bach K, Overas CK, et al. Effectiveness of App-Delivered, Tailored Self-management Support for Adults With Lower Back Pain-Related Disability: A selfBACK Randomized Clinical Trial. *JAMA Intern Med*. Oct 1 2021;181(10):1288-1296. doi:10.1001/jamainternmed.2021.4097

52. Schroder K, Oberg B, Enthoven P, Hedevik H, Abbott A. Improved adherence to clinical guidelines for low back pain after implementation of the BetterBack model of care: A stepped cluster randomized controlled trial within a hybrid type 2 trial. *Physiother Theory Pract*. Mar 1 2022:1-15. doi:10.1080/09593985.2022.2040669

53. Sharma S, Traeger AC, O'Keeffe M, et al. Effect of information format on intentions and beliefs regarding diagnostic imaging for non-specific low back pain: A randomised controlled trial in members of the public. *Patient Educ Couns*. Mar 2021;104(3):595-602. doi:10.1016/j.pec.2020.08.021

54. Shebib R, Bailey JF, Smittenaar P, Perez DA, Mecklenburg G, Hunter S. Randomized controlled trial of a 12-week digital care program in improving low back pain. *NPJ Digit Med*. 2019;2:1. doi:10.1038/s41746-018-0076-7

55. Simula AS, Jenkins HJ, Hancock MJ, Malmivaara A, Booth N, Karppinen J. Patient education booklet to support evidence-based low back pain care in primary care - a cluster randomized controlled trial. *BMC Fam Pract*. Sep 7 2021;22(1):178. doi:10.1186/s12875-021-01529-2

56. Slater H, Briggs AM, Watkins K, Chua J, Smith AJ. Translating evidence for low back pain management into a consumer-focussed resource for use in community pharmacies: a cluster-randomised controlled trial. *PLoS One*. 2013;8(8):e71918. doi:10.1371/journal.pone.0071918

57. Stuart Donaldson C, Stanger LM, Donaldson MW, Cram J, Skubick DL. A randomized crossover investigation of a back pain and disability prevention program: Possible mechanisms of change. *J Occup Rehabil*. Jun 1993;3(2):83-94. doi:10.1007/BF01078161

58. Suman A, Schaafsma FG, van Dongen JM, et al. Effectiveness and cost-utility of a multifaceted eHealth strategy to improve back pain beliefs of patients with non-specific low back pain: a cluster randomised trial. *BMJ Open*. Dec 5 2019;9(12):e030879. doi:10.1136/bmjopen-2019-030879

59. Rhon DI, Mayhew RJ, Greenlee TA, Fritz JM. The influence of a MOBile-based video Instruction for Low back pain (MOBIL) on initial care decisions made by primary care providers: a randomized controlled trial. *BMC Fam Pract*. Oct 9 2021;22(1):200. doi:10.1186/s12875-021-01549-y

60. Riva S, Camerini AL, Allam A, Schulz PJ. Interactive sections of an Internet-based intervention increase empowerment of chronic back pain patients: randomized controlled trial. *J Med Internet Res*. Aug 13 2014;16(8):e180. doi:10.2196/jmir.3474

61. Rosenberg NR, Petersen SB, Begtrup LM, et al. Early Occupational Intervention for People with Low Back Pain in Physically Demanding Jobs: 1-year Follow-up Results of the Randomized Controlled GOBACK Trial. *Spine (Phila Pa 1976)*. Mar 15 2021;46(6):347-355. doi:10.1097/BRS.0000000000003793

62. Ryynanen K, Oura P, Simula AS, et al. Effectiveness of training in guideline-oriented biopsychosocial management of low-back pain in occupational health services - a cluster randomized controlled trial. *Scand J Work Environ Health*. Jul 1 2021;47(5):367-376. doi:10.5271/sjweh.3959

63. Zarifsanaiey N, Yazdani Z, Karimian Z, Shahraki HR, Ghaffarinejad F. The Effect of Interactive Multimedia And Illustrated Booklet On Knowledge, Attitude, And Behavior In Preventing Lower Back Pain Among Working Nurses: A Randomized Controlled Trial. 2021;doi:<https://doi.org/10.21203/rs.3.rs-682719/v1>

64. Burns SA, Cleland JA, Rivett DA, et al. When Treating Coexisting Low Back Pain and Hip Impairments, Focus on the Back: Adding Specific Hip Treatment Does Not Yield Additional Benefits-A Randomized Controlled Trial. *J Orthop Sports Phys Ther*. Dec 2021;51(12):581-601. doi:10.2519/jospt.2021.10593

65. Carpenter KM, Stoner SA, Mundt JM, Stoelb B. An online self-help CBT intervention for chronic lower back pain. *Clin J Pain*. Jan 2012;28(1):14-22. doi:10.1097/AJP.0b013e31822363db

66. Garcia LM, Birckhead BJ, Krishnamurthy P, et al. An 8-Week Self-Administered At-Home Behavioral Skills-Based Virtual Reality Program for Chronic Low Back Pain: Double-Blind, Randomized, Placebo-Controlled Trial Conducted During COVID-19. *J Med Internet Res*. Feb 22 2021;23(2):e26292. doi:10.2196/26292

67. Garaud T, Gervais C, Szekely B, Michel-Cherqui M, Dreyfus JF, Fischler M. Randomized study of the impact of a therapeutic education program on patients suffering from chronic low-back pain who are treated with transcutaneous electrical nerve stimulation. *Medicine (Baltimore)*. Dec 2018;97(52):e13782. doi:10.1097/MD.0000000000013782

68. Garcia AN, Costa Lda C, da Silva TM, et al. Effectiveness of back school versus McKenzie exercises in patients with chronic nonspecific low back pain: a randomized controlled trial. *Phys Ther*. Jun 2013;93(6):729-47. doi:10.2522/ptj.20120414

69. Jinnouchi H, Matsudaira K, Kitamura A, et al. Effects of brief self-exercise education on the management of chronic low back pain: A community-based, randomized, parallel-group pragmatic trial. *Mod Rheumatol*. Jul 2020;31(4):890-898. doi:10.1080/14397595.2020.1823603

70. Maggi L, Celletti C, Mazzarini M, Blow D, Camerota F. Neuromuscular taping for chronic non-specific low back pain: a randomized single-blind controlled trial. *Aging Clin Exp Res*. May 2022;34(5):1171-1177. doi:10.1007/s40520-021-02029-0

71. Moraes EB, Martins Junior FF, Silva LBD, Garcia JBS, Mattos-Pimenta CA. Self-efficacy and fear of pain to movement in chronic low back pain: an intervention developed by nurses. *Rev Gaucha Enferm*. 2021;42:e20200180. doi:10.1590/1983-1447.2021.20200180

72. Rabiei P, Sheikhi B, Letafatkar A. Comparing Pain Neuroscience Education Followed by Motor Control Exercises With Group-Based Exercises for Chronic Low Back Pain: A Randomized Controlled Trial. *Pain Pract*. Mar 2021;21(3):333-342. doi:10.1111/papr.12963

73. Shimo K, Hasegawa M, Mizutani S, Hasegawa T, Ushida T. Effects of a 12-week workplace counseling program on physical activity and low back pain: A pilot randomized controlled study. *J Back Musculoskelet Rehabil*. 2021;34(5):845-852. doi:10.3233/BMR-200178

74. Walsh N, Jones L, Phillips S, et al. Facilitating Activity and Self-management for people with Arthritic knee, hip or lower back pain (FASA): A cluster randomised controlled trial. *Musculoskelet Sci Pract*. Dec 2020;50:102271. doi:10.1016/j.msksp.2020.102271

75. Williams A, Wiggers J, O'Brien KM, et al. Effectiveness of a healthy lifestyle intervention for chronic low back pain: a randomised controlled trial. *Pain*. Jun 2018;159(6):1137-1146. doi:10.1097/j.pain.0000000000001198

76. Amaral DDV, Miyamoto GC, Franco KFM, et al. Examination of a Subgroup of Patients With Chronic Low Back Pain Likely to Benefit More From Pilates-Based Exercises Compared to an Educational Booklet. *J Orthop Sports Phys Ther*. Apr 2020;50(4):189-197. doi:10.2519/jospt.2019.8839

77. Berlowitz J, Hall DL, Joyce C, et al. Changes in Perceived Stress After Yoga, Physical Therapy, and Education Interventions for Chronic Low Back Pain: A Secondary Analysis of a Randomized Controlled Trial. *Pain Med*. Oct 1 2020;21(10):2529-2537. doi:10.1093/pm/pnaa150

78. Caldas VVA, Maciel DG, Cerqueira MS, et al. Effect of Pain Education, Cryotherapy, and Transcutaneous Electrical Nerve Stimulation on the Pain, Functional Capacity, and Quality of Life in Patients With Nonspecific Chronic Low Back Pain: A Single-Blind Randomized Controlled Trial. *Am J Phys Med Rehabil*. Mar 1 2021;100(3):243-249. doi:10.1097/PHM.0000000000001552

79. Chiauzzi E, Pujol LA, Wood M, et al. painACTION-back pain: a self-management website for people with chronic back pain. *Pain Med*. Jul 2010;11(7):1044-58. doi:10.1111/j.1526-4637.2010.00879.x

80. Darnall BD, Roy A, Chen AL, et al. Comparison of a Single-Session Pain Management Skills Intervention With a Single-Session Health Education Intervention and 8 Sessions of Cognitive Behavioral Therapy in Adults With Chronic Low Back Pain: A Randomized Clinical Trial. *JAMA Netw Open*. Aug 2 2021;4(8):e2113401. doi:10.1001/jamanetworkopen.2021.13401

81. Ghadyani L, Tavafian SS, Kazemnejad A, Wagner J. Work-Related Low Back Pain Treatment: A Randomized Controlled Trial from Tehran, Iran, Comparing Multidisciplinary Educational Program versus Physiotherapy Education. *Asian Spine J*. Aug 2016;10(4):690-6. doi:10.4184/asj.2016.10.4.690

82. Ibrahim MI, Zubair IU, Shafei MN, Ahmad MI, Yaacob NM. Interactive Low Back Pain Intervention Module Based on the Back School Program: A Cluster-Randomized Experimental Study Evaluating Its Effectiveness among Nurses in Public Hospitals. *Int J Environ Res Public Health*. Aug 14 2020;17(16)doi:10.3390/ijerph17165916

83. Kim SK, Kim HS, Chung SS. Effects of an Individualized Educational Program for Korean Patients With Chronic Low Back Pain: A Randomized Controlled Trial. *J Nurs Res*. Dec 1 2021;29(6):e177. doi:10.1097/jnr.0000000000000455

84. Kohns DJ, Urbanik CP, Geisser ME, Schubiner H, Lumley MA. The Effects of a Pain Psychology and Neuroscience Self-Evaluation Internet Intervention: A Randomized Controlled Trial. *Clin J Pain*. Sep 2020;36(9):683-692. doi:10.1097/AJP.0000000000000857

85. Marshall A, Joyce CT, Tseng B, et al. Changes in Pain Self-Efficacy, Coping Skills, and Fear-Avoidance Beliefs in a Randomized Controlled Trial of Yoga, Physical Therapy, and Education for Chronic Low Back Pain. *Pain Med*. Apr 8 2022;23(4):834-843. doi:10.1093/pm/pnab318

86. Mendes Tozim B, Thomaz de Aquino Nava G, Zuliani Stroppa Marques AE, Tavella Navega M. Efficacy of the Pilates versus general exercises versus educational workshops on neuromuscular parameters: A randomized controlled trial. *J Bodyw Mov Ther*. Apr 2021;26:420-427. doi:10.1016/j.jbmt.2020.08.012

87. Orhan C, Lenoir D, Favoreel A, et al. Culture-sensitive and standard pain neuroscience education improves pain, disability, and pain cognitions in first-generation Turkish migrants with chronic low back pain: a pilot randomized controlled trial. *Physiother Theory Pract*. May 2021;37(5):633-645. doi:10.1080/09593985.2019.1639231

88. Rios JCS, Hua FY, Safons MP. Posture-focused self-management programme improves pain and function in older people with chronic low back pain: a randomised controlled trial. *International Journal of Therapy and Rehabilitation*. 2020;doi:10.12968/ijtr.2018.0082

89. Unal M, Evci KE, Kocaturk M, Algun ZC. Investigating the effects of myofascial induction therapy techniques on pain, function and quality of life in patients with chronic low back pain. *J Bodyw Mov Ther*. Oct 2020;24(4):188-195. doi:10.1016/j.jbmt.2020.07.014

90. Yang CY, Tsai YA, Wu PK, Ho SY, Chou CY, Huang SF. Pilates-based core exercise improves health-related quality of life in people living with chronic low back pain: A pilot study. *J Bodyw Mov Ther*. Jul 2021;27:294-299. doi:10.1016/j.jbmt.2021.03.006

91. Li T, Wang S, Cheng K, et al. Comparing the efficacy of two different temperature stimulation in warm acupuncture on acute low back pain: A randomized controlled trial. Journal: Article. *Integrative Medicine Research*. 2022;11(1)doi:10.1016/j.imr.2021.100748
